# Total synthesis of interleukin-2 *via* a tunable backbone modification strategy†

**DOI:** 10.1039/d2sc05660g

**Published:** 2023-01-06

**Authors:** Hongxiang Wu, Yi Tan, Wai Lok Ngai, Xuechen Li

**Affiliations:** a Department of Chemistry, State Key Laboratory of Synthetic Chemistry, The University of Hong Kong Hong Kong SAR P. R. China xuechenl@hku.hk; b Laboratory for Marine Drugs and Bioproducts, Qingdao National Laboratory for Marine Science and Technology Qingdao 266237 P. R. China

## Abstract

Chemical synthesis of hydrophobic proteins presents a formidable task as they are often difficultly achieved *via* peptide synthesis, purification, and peptide ligation. Thus, peptide solubilizing strategies are needed to integrate with peptide ligation to achieve protein total synthesis. Herein, we report a tunable backbone modification strategy, taking advantage of the tunable stability of the Cys/Pen ligation intermediate, which allows for readily introducing a solubilizing tag for both peptide purification and ligation processes. The effectiveness of this strategy was demonstrated by the chemical synthesis of interleukin-2.

## Abstract 2

The stability of Cys/Pen ligation intermediate can be tuned to equip with solubilizing tags. Such a strategy can effectively solve the problem of purifying hydrophobic peptides and perform peptide ligation, enabling the total synthesis of IL-2.

## Graphical abstract

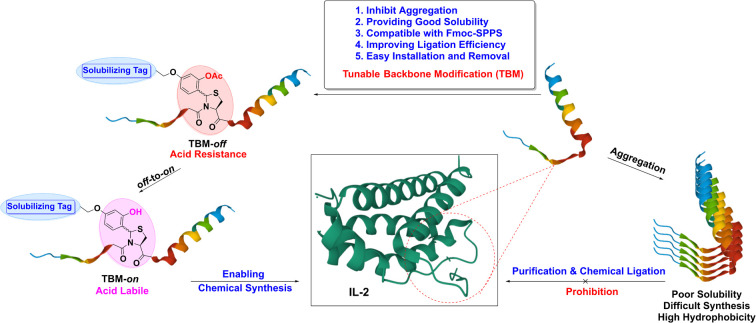

## 1 Introduction

The past two decades have witnessed the rapid development of innovative peptide ligation methodologies^1–9^ and strategies,^10–13^ which have led to dramatic changes in the scope of protein chemical synthesis. Many proteins of biological interests have been chemically synthesized and used to correlate the protein structure to function.^14–24^ In particular, protein chemical synthesis provides a unique means to generate proteins with tailor-made modifications, which are difficult or impossible for expression systems.^25–36^ Despite these achievements in protein chemical synthesis, the synthesis of proteins or peptides with aggregation-prone properties remains a challenging task and requires case by case analysis and study. The dilemma can be classified into two types: (a) the peptides aggregate on the resin beads during solid-phase peptide synthesis (SPPS), leading to peptide elongation failure or poor product quality;^37,38^ (b) the peptide products tend to form insoluble or colloidal solids after successful synthesis and cleavage from the resin, which makes reverse-phase high-performance liquid chromatography (RP-HPLC) purification or performing peptide ligation difficult.^39^ To address this difficulty, many efforts have been devoted, involving the employment of solubilizing tags,^40–51^*O*-acyl isopeptide,^52–56^ removable backbone modification,^57–59^ and special ligation solvent conditions.^60–64^

Interleukin-2 (IL-2) is a cytokine playing an important role in cancer immunotherapy. Indeed, in clinical treatment, the effect of IL-2 is a bit complex because of its dual functional roles in T cell.^65^ It can not only promote activation and proliferation of natural killer cells and cytotoxic T cells to destroy tumor cells^66^ but also enhance regulatory T cell activities to downregulate T cell cytotoxicity.^67^ Therefore, the chemical synthesis of IL-2 could offer an opportunity to construct site-specific modified IL-2 variants with more focused biofunctions, which may lead to better clinical outcomes in cancer or immunosuppression treatments.

IL-2 is a typical difficult protein for chemical synthesis. To achieve the goal of chemical synthesis of IL-2, the primary task is to overcome the obstacle of preparation of the extremely hydrophobic and highly aggregated C-terminal region. In 2015, after more than 15 years of efforts, Hojo's group achieved the first total synthesis of IL-2 using both *O*-acyl isopeptides and picolyl ester protecting groups.^54^ Later, Bode's group reported the second synthesis of IL-2 by performing KAHA ligation at several mutated sites and using *O*-acyl isopeptides for segment preparation.^55^ The two studies showcase the current state-of-the-art of protein synthesis. However, new NCL-compatible strategy to enable the robust synthesis of the IL-2 C-terminal region is appealing but remains to be explored, which will expand the NCL application scope to such type of highly hydrophobic and aggregated peptides.

**Fig. 1:**
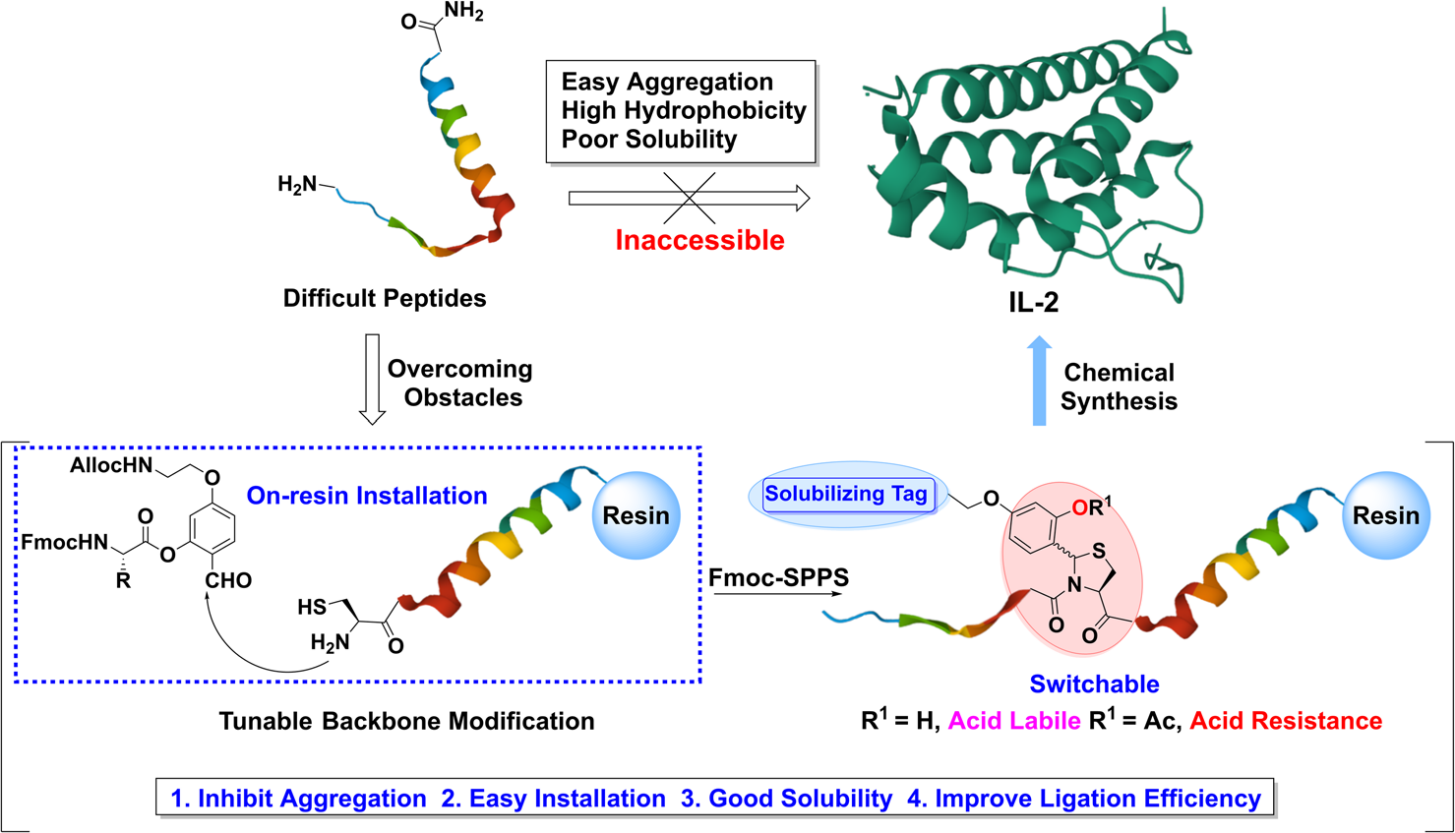
TBM strategy.

Herein, we report a tunable backbone modification (TBM) strategy (Fig. 1) to enable the robust and straightforward synthesis of IL-2. Notably, the development of TBM has not only allowed the synthesis and purification of difficult peptides but also contributed to the first successful application of NCL in highly aggregated IL-2 C-terminal part, demonstrating the power of TBM in solving problematic protein synthesis and poor ligation efficiency of insoluble or colloidal peptides. Moreover, the concise installation protocol, *in situ* on-resin formation, and high compatibility with well-established NCL techniques make TBM an easily adopted approach in difficult protein chemical synthesis.

## 2 Results and discussion

**Fig. 2:**
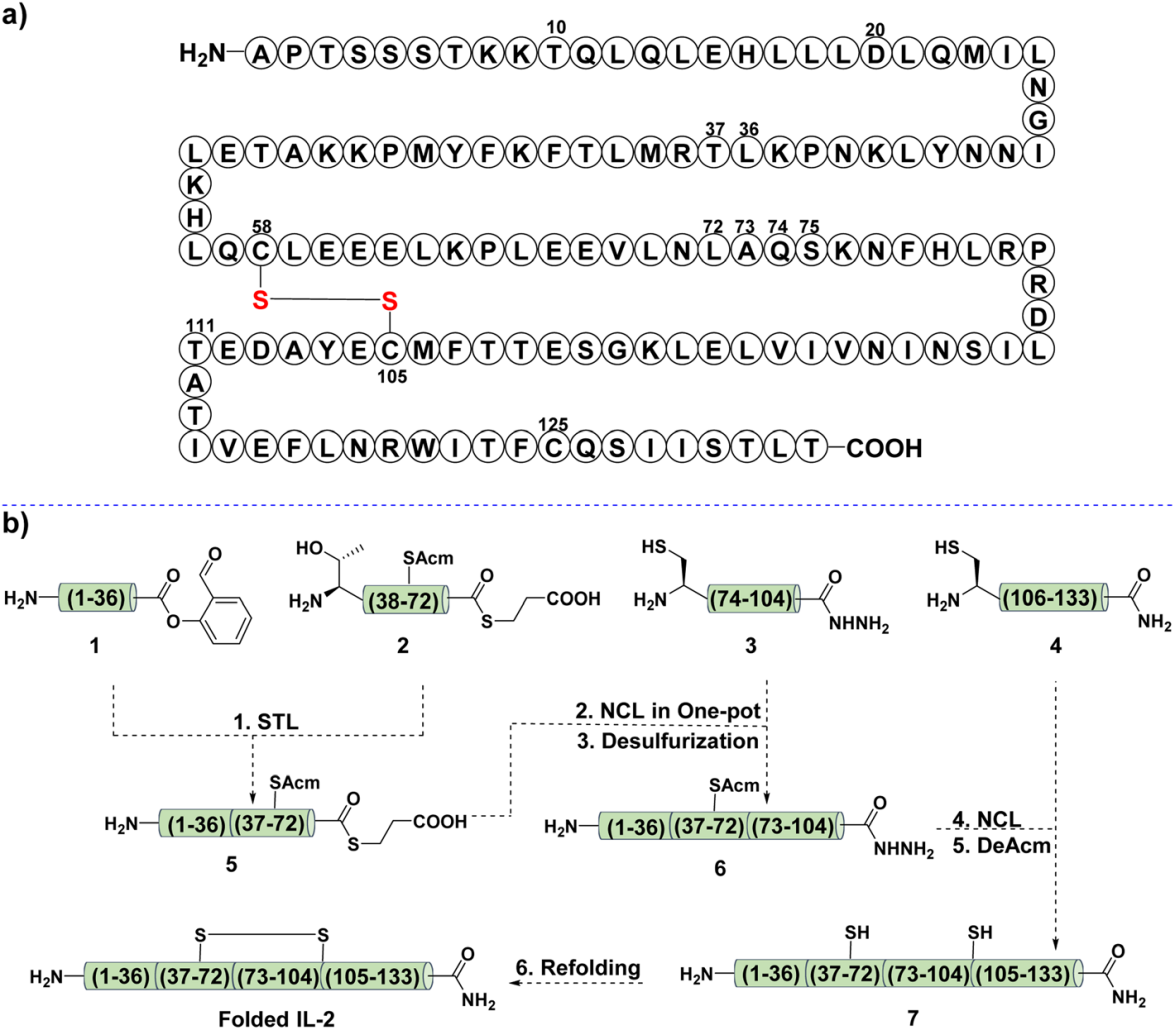
(a) Sequence of interleukin-2. (b) The first proposed synthetic route.

As shown in Fig. 2a, IL-2 consists of 133 amino acid residues, and two of the three Cys residues are involved in the disulfide bond formation (Cys58 and Cys105). Our initial attempt was to divide the whole sequence into four segments, 1, 2, 3, and 4. The planned synthetic scheme was sequential N-to-C ligation (Fig. 2b). Firstly, a Ser/Thr ligation (STL) between segments 1 and 2 would provide the ligated peptide thioester 5, then, it would be subjected to the NCL reaction with 3 to afford the product 6. Finally, another NCL would be performed between 6 and 4 to give 7, the desired linear IL-2.

**Fig. 3:**
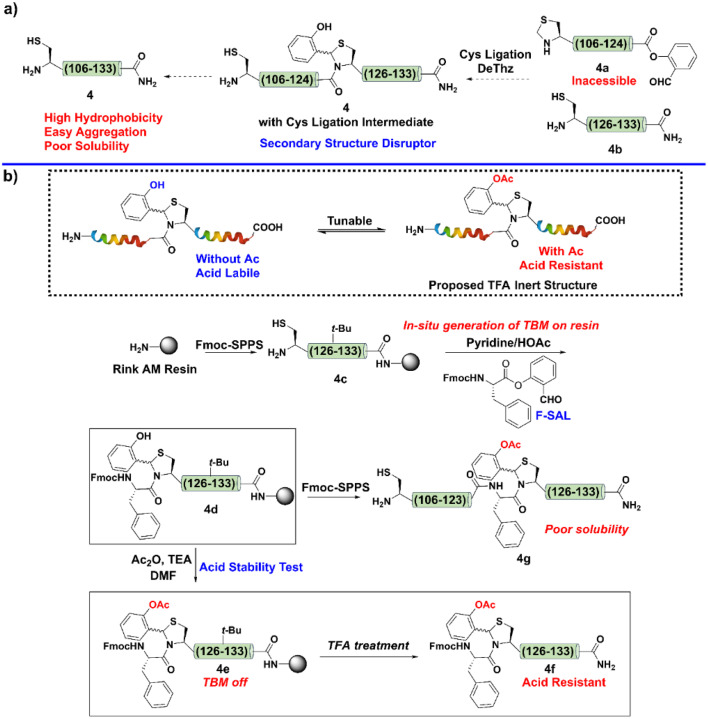
(a) Synthetic plan for 4 with Cys ligation intermediate. (b) Synthetic scheme of 4g.

Following this design (Fig. 2b), we first attempted to synthesize the C-terminal segment 4*via* SPPS, but failed (Fig. 3a). We noticed that this difficult fragment contained Cys125, a potential site to apply our Cys ligation, as the resultant *N*,*S*-benzylidene acetal, the ligation intermediate, has been proven to be powerful for interrupting peptide secondary structure and preventing aggregation.^51^ However, the preparation of the corresponding peptide salicylaldehyde ester 4a failed again due to the poor solubility.

Next, we aimed to install a Cys ligation intermediate during Fmoc-SPPS to inhibit the aggregation tendency and enable peptide elongation. To this end, after the peptide was elongated to Cys125, the sidechain protecting group of the Cys was removed to give a resin-bound peptide 4c (Fig. 3b). Next, a Fmoc-protected Phe salicylaldehyde ester (F-SAL) was dissolved by a pyridine/HOAc buffer (1 : 1) and subjected to react with the resin-bound Cys peptide 4c. After overnight reaction, the resin was simply washed by dichloromethane to afford the desired product 4d bearing a Cys ligation intermediate in quantitative yield. Unfortunately, the Cys ligation intermediate was TFA labile, and the *N*,*S*-benzylidene acetal was converted to the native Phe–Cys amide linkage during the global deprotection step to afford 4d as its native aggregation form.

To overcome this obstacle, we hypothesized that capping the phenolic hydroxyl group of the Cys ligation intermediate with acetyl group would make it acid-resistant (Fig. 3b). After obtaining the resin-bound peptide 4d with the Cys ligation intermediate, we performed Ac capping and TFA deprotection to test our hypothesis. Remarkably, as expected, the Cys ligation intermediate with Ac capping was very stable under TFA conditions (Fig. 3b). To be specific, the *N*,*S*-benzylidene acetal could be deemed to be a tunable switch, *i.e.*, once the phenolic hydroxyl group was masked, it turned into an “off” state to resist TFA deprotection; in contrast, after Ac removal, it turned into an “on” state and could be removed by TFA.

Moreover, TBM can be easily installed *via* a very simple on-resin *in situ* generation step, which avoids tedious synthesis. Encouraged by this result, after the installation of TBM, we performed a standard Fmoc-SPPS and final Ac capping step to reserve TBM on the sequence to finish the synthesis of 4g.

**Fig. 4:**
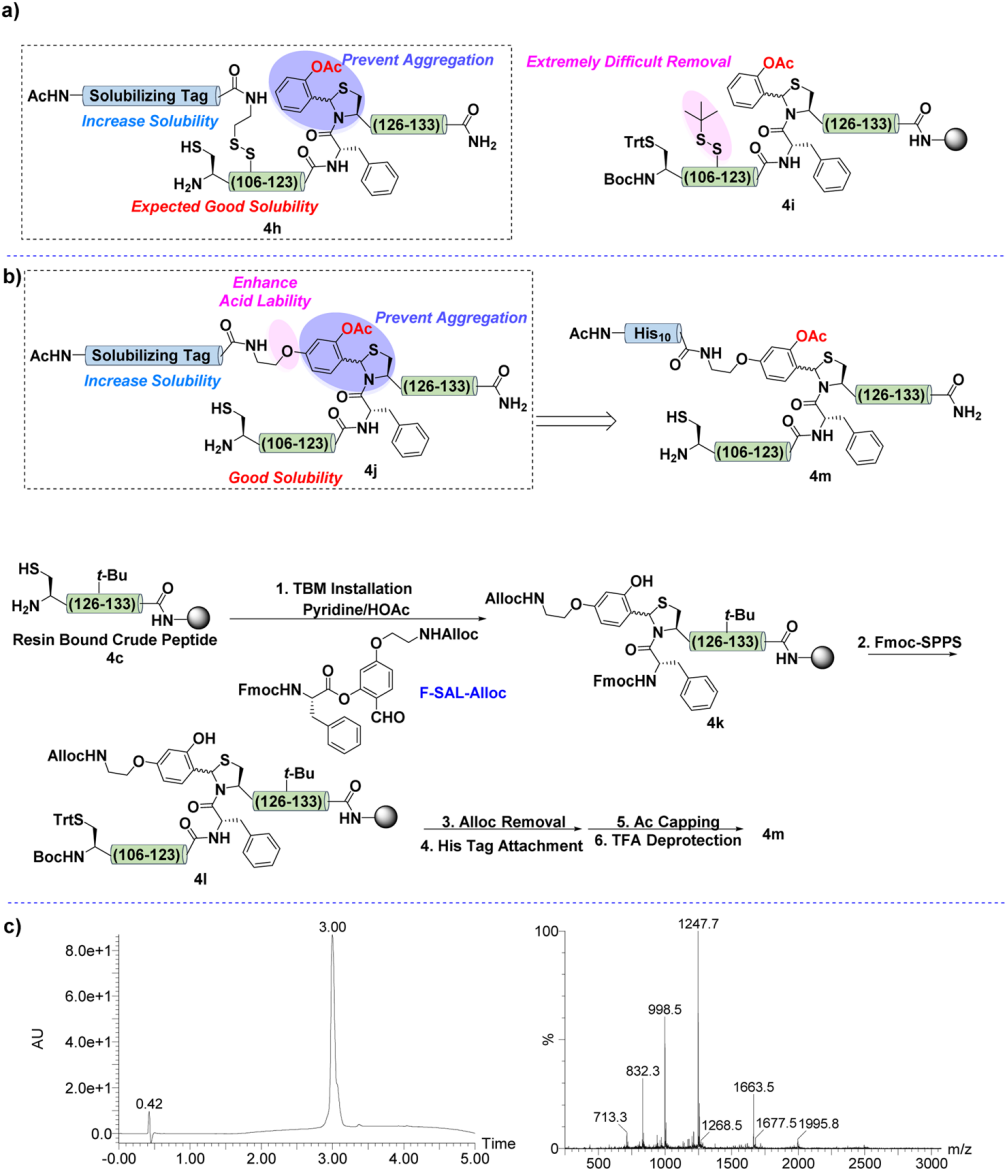
(a) Synthetic design of TBM with RST. (b) Synthetic scheme of 4m. (c) UV-trace of 4m (left) and mass spectrum of 4m (right).

Indeed, 4 could be successfully synthesized as its TBM form 4g (Fig. 3b), which supported that TBM is a useful strategy to overcome peptide aggregation during Fmoc-SPPS. However, the purification of fragment 4g was unsuccessful because of its high hydrophobicity in aqueous acetonitrile. This preliminary result indicated that disrupting the secondary structure of this IL-2 C-terminal region only prevented its aggregation behavior on the resin and permitted its synthesis, while its hydrophobic nature still hampered the preparation. To alleviate the hydrophobicity, the introduction of a solubilizing tag might be required. Thus, we planned to combine TBM with our reducible solubilizing tags (RSTs) strategy;^50^ in other words, the TBM would interrupt the aggregation to guarantee peptide elongation and RST would provide good solubility to enable the purification. With this in mind, Ala112 was mutated to Cys for installation of RST. As shown in Fig. 4a, the synthetic target was revised to 4h.

Naturally, the synthesis was performed by following the same synthetic route of 4g except for replacing Ala112 with Cys. After that, the deprotection of the Cys sidechain was carried out under strong reductive conditions to ensure the installation of the solubilizing tag. However, the *S-tert*-butylthiol protecting group was extremely difficult to remove (Fig. 4a). The reason might be the highly hydrophobic property of this sequence, and the sidechain of Cys was buried by surrounding hydrophobic residues.

As a consequence, we intended to develop an improved version of TBM, which contained both aggregation disruption and solubility improvement capabilities. The hypothesis was to introduce an alkoxy chain at the para position of the aldehyde group of the salicylaldehyde amino acid ester, as shown in Fig. 4b. The alkoxy group would not only render the TBM more acid labile for removal but also provide an opportunity for solubilizing tag installation after finishing Fmoc-SPPS. Moreover, this design would enable the attachment of the solubilizing tag on the benzene ring of the TBM, which is located at the exterior part of the twisted structure of the peptide main-chain and is less possible to be buried by other residues.

Following the above analysis, SAL ester F-SAL-Alloc was synthesized and subjected to the installation of TBM. After the main-chain elongation, the Alloc group was deprotected to allow the attachment of the solubilizing tag (Fig. 4b). Notably, this time, the IL-2 C-terminal region was successfully constructed and purified as 4m in 12% yield, the single, sharp and symmetric peak of this fragment in HPLC suggesting the effectiveness of this TBM strategy in breaking the secondary structure and solving the hydrophobicity issue (Fig. 4c) as hydrophobic peptides normally resulted in broad chromatographic peaks during HPLC and encountered incomplete elution.^68^ Moreover, the lack of suitable installation sites of the reported solubilizing tag strategies supports that TBM is a high valuable strategy for the synthesis of the IL-2 C-terminus.

With this success in hand, the other three fragments, 1, 2, and 3, were synthesized in 31%, 55%, and 9% yield, respectively (Fig. 2b). One thing to note was the poor solubility of fragment 3, which required HMB installation at Gly98 for the synthesis and DMSO assistance for HPLC purification. After that, STL was performed between 1 and 2 to give the desired ligation product 5, which was subjected to the typical NCL with 3 in a one-pot manner (Fig. 2b). Unfortunately, no reaction occurred after several optimizations had been tried, including using HPLC purified 5, high reaction concentration, saturated guanidine aqueous solution (8 M), and heating to 37 °C. This was attributed to the aggregation tendency of 3 during the reaction.

**Fig. 5:**
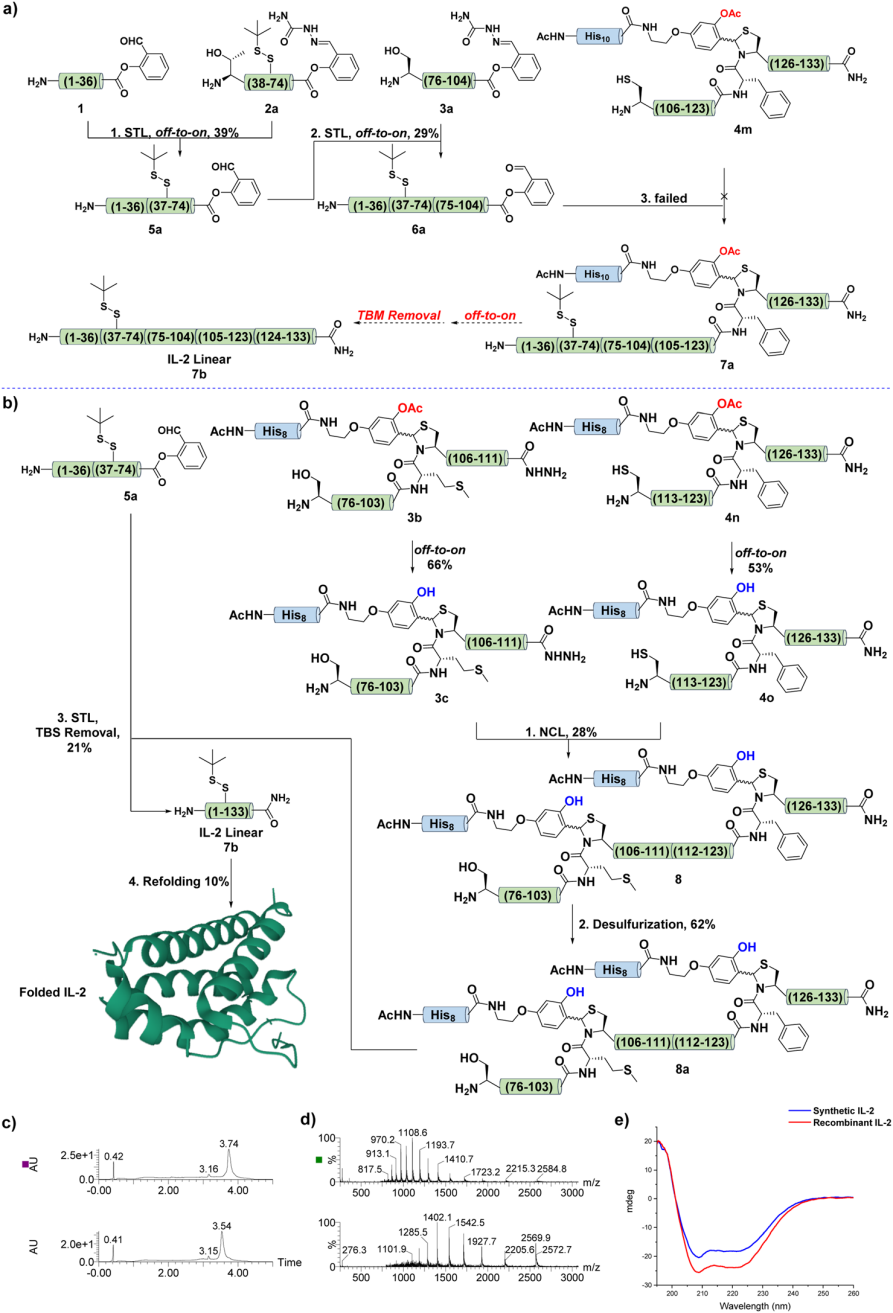
(a) The second synthetic scheme for IL-2. (b) The third synthetic scheme with two TBMs version. (c) UV traces of IL-2 before and after refolding. (d) MS spectra of IL-2 before and after refolding. (e) CD spectra of the folded synthetic and recombinant IL-2.

The failure in the synthesis of 6 prompted us to revise the synthetic scheme. As shown in Fig. 5a, we intended to perform an N-to-C sequential STL between 1, 2a, and 3a to afford 6a, then the Cys ligation between 6a and 4m would provide the desired product 7a, which underwent off-to-on deprotection and acidolysis to give the linear IL-2 7b. Next, fragments 2a and 3a were prepared, and 3a showed similar poor solubility as 3, as expected. With these fragments in hand, the first STL was performed between 1 and 2a to afford the desired product 5a in 39% yield. After that, 5a was ligated with 3a under typical STL conditions to generate the corresponding ligation intermediate, which was later treated with TFA and pyruvic acid to provide the desired SAL ester 6a in 29% yield.

One thing to be noted was the slow formation of the gel in the reaction mixture during the ligation, suggesting the aggregation tendency of 3a in the ligation process. Fortunately, after ligation, the N-terminal part (1–74) of the IL-2 seemed provide 6a with good solubility to permit further operation. Undoubtedly, 6a was subjected to ligation with 4m according to the synthetic scheme. However, no desired product was observed, even after changing the reaction solution to pH = 3.0 aqueous buffer containing 8 M guanidine, which normally worked in handling difficult-to-react peptides for Cys ligation.

To address this issue, we hypothesized that although the N-terminal part of 6a provided it with good solubility, the C-terminal still contained the secondary structure, which buried the reactive site and inhibited the ligation. Therefore, to tackle this problematic synthesis and test our hypothesis, we further revised the synthetic scheme (Fig. 5b). The synthesis of the C-terminal region (75–133) was redesigned, and two TBMs were introduced in the construction of fragments 3b and 4n. It was expected that the installation of TBM at Met104-Cys105 would provide 3b with good reactivity and solubility.

Next, 3b and 4n were successfully synthesized by following the protocol established above, and were converted to the corresponding 3c and 4o (Fig. 5b). Remarkably, the TBM strategy exhibited compatibility with the peptide hydrazide chemistry, and permitted hydrophobic and aggregated IL-2 fragments to be synthesized in the hundreds of milligram scale. With these two fragments in hand, an NCL was performed between 3c and 4o to afford the desired product 8. This successful application of TBM in NCL for handling remarkably difficult sequence, particularly the NCL that was performed at a highly steric hindered Thr site, indicated that TBM could totally break the aggregation tendency of such type of difficult peptide and provide good solubility and reactivity.

After that, 8 was subjected to a desulfurization protocol to give the corresponding product 8a in 62% yield. This promising result also supported the feasibility of TBM in promoting the solubility and desulfurization of difficult peptides, as it has been proven that poorly solubilized peptides or proteins suffered from sluggish and incomplete desulfurization.^39,48^ Finally, the STL between fragments 8a and 5a provided the desired ligation intermediate, which was subjected to TFA treatment for TBM removal and afforded the final linear IL-2 7 in 21% yield (Fig. 5b). Next, the linear IL-2 sequence was refolded following the reported procedure^54,55^ and subjected to HPLC purification to afford folded the IL-2 in 10% yield. The UPLC-MS trace (Fig. 5c and d) and circular dichroism (CD) spectra (Fig. 5e) supported the folding result of IL-2.^54,55^

## 3 Conclusion

In conclusion, a TBM strategy has been developed and employed in the synthesis of highly aggregated and hydrophobic C-terminal region of IL-2, and it has led to the successful total synthesis of IL-2. The deprotection or preservation of TBM can be easily adjusted *via* a simple Ac capping or decapping step, which enables it to fulfill the requirement of various kinds of situations during complex protein chemical synthesis. It not only enabled the first successful application of NCL in the synthesis of such type of difficult peptides but also permitted the desulfurization step. In addition, the concise and efficient installation protocol, *in situ* on-resin TBM formation, and compatibility with peptide hydrazide preparation approach may promote it as an easily adopted strategy for the chemical synthesis of proteins bearing difficult sequences, such as IL-2. Moreover, the combination of this TBM strategy and thiol-derived amino acids, such as penicillamine, may further expand the application scope of TBM to enable a wide range of aggregative and hydrophobic peptides or proteins to be chemically synthesized.

## Data availability

Experimental data is available in the accompanying ESI.†

## Author contributions

X. L. conceived the idea and supervised the project; H. W. conducted the majority of the experimental work; Y. T. performed the preliminary attempt in the 1^st^ trial of chemical synthesis of IL-2; W. L. N. helped to prepare some peptide fragments.

## Conflicts of interest

There are no conflicts to declare.

## Supplementary Material

SC-014-D2SC05660G-s001
