# Supplementary material for: Total synthesis of interleukin-2 via a tunable backbone modification strategy
Source: Chem Sci. 2023 Jan 6;14(6):1582–9. doi: 10.1039/d2sc05660g (PMC9906654; doi:10.1039/d2sc05660g)
Supplement: SC-014-D2SC05660G-s001 [file SC-014-D2SC05660G-s001.pdf]

Electronic Supplementary Material (ESI) for Chemical Science.
This journal is © The Royal Society of Chemistry 2023

Total Synthesis of Interleukin-2 via a Tunable Backbone Modification Strategy

Hongxiang Wu,[1] Yi Tan,[1] Wai Lok Ngai[1] and Xuechen Li*[1, 2]

[1]Department of Chemistry, State Key Laboratory of Synthetic Chemistry, The University of Hong Kong, Pokfulam Road, Hong Kong SAR, P. R. China.

[2]Laboratory for Marine Drugs and Bioproducts, Qingdao National Laboratory for Marine Science and Technology, Qingdao, R. R. China, 266237.

E-mail: xuechenl@hku.hk

Supplementary Information

1. General Information

2. General Experimental Procedures

3. Synthesis of Interleukin-2 (IL-2) C-terminus

4. The First Synthetic Attempt of Interleukin-2 (IL-2)

5. The Second Synthetic Attempt of Interleukin-2 (IL-2)

6. The Third Synthetic Attempt of Interleukin-2 (IL-2)

7. Synthesis of Salicylaldehyde Ester

8. ^{1}H and ^{13}C NMR spectra

1. General Information

1.1 Materials and methods and abbreviations

All commercial materials (purchased from Aldrich, ChemImpex, Fluka and GL Biochem) were used without further purification. All solvents were reagent grade or HPLC grade (RCI or DUKSAN). Dry dichloromethane (CH_2Cl_2) was distilled from calcium hydride (CaH_2). All reversed-phase (RP) high-performance liquid chromatography (HPLC) separations involved a mobile phase of 0.1% trifluoroacetic acid (TFA) (v/v) in acetonitrile (CH_3CN)/0.1% TFA (v/v) in water (H_2O) were performed with a Waters HPLC system equipped with a photodiode array detector (Waters 2996) using a Vydac 214TPTM C4 column (5 µm, 300 Å, 4.6 x 250 mm) at a flow rate of 0.6 mL/min for analytical HPLC and Vydac 214TPTM C4 column (10 µm, 300 Å, 22 x 250 mm) or Vydac 218TPTM C18 column (10 µm, 300 Å, 22 x 250 mm) at a flow rate of 10 mL/min for preparative HPLC. Low-resolution mass spectral (MS) analyses were performed with a Waters 3100 mass spectrometer using electrospray ionization (ESI, in positive mode unless otherwise specified). The results were analyzed with Waters Empower software. Calculated masses were based upon the most abundant isotope of a given ion. Analytical TLC was performed on E. Merck silica gel 60 F254 plates and visualized under UV light (254 nm) or by staining with ninhydrin or 5 % sulfuric acid in methanol. Silica flash column chromatography was performed on E. Merck 230-400 mesh silica gel 60. ^{1}H and ^{13}C nuclear magnetic resonance (NMR) spectra were recorded at 298 K on Bruker Avance DRX 300 FT-NMR Spectrometer at 75 MHz for ^{13}C NMR or Bruker Avance DRX 400 FT-NMR spectrometer at 400 MHz for ^{1}H NMR and 100 MHz for ^{13}C NMR or Bruker Avance DRX 600 FT-NMR spectrometer at 150 MHz for ^{13}C NMR. Chemical shifts are reported in parts per million (ppm) and are referenced to solvent residual signals: $CDCl_3$ (δ 7.26 [^{1}H]). ^{1}H NMR data is reported as chemical shift (δ), relative integral, multiplicity (s = singlet, d = doublet,

t = triplet, dd = doublet of doublet, td = triplet of doublet), coupling constant (*J* Hz). LCMS = Liquid chromatography mass-spectrometry; PG = protecting groups; SAL = salicylaldehyde; DMF = dimethylformamide; TIPS = triisopropylsilane.

2. General Experimental Procedures

2.1 Standard Protocol of Solid-phase Peptide Synthesis (SPPS)

The commercially available 2-chlorotrityl resin (CS Biochem, loading: ~0.5 mmol/g) was employed in the solid phase peptide synthesis of peptides. FmocHN-Xaa-COOH (4.0 equiv) and diisopropylethylamine (8.0 equiv) were dissolved in dichloromethane, then this solution was poured into a reaction vial containing 2-chlorotrityl resin, and the mixture was shaken at room temperature (r. t.) for 2 h. After that, this amino acid bound resin was washed with dimethylformamide and dichloromethane. The resin was subjected to Fmoc-SPPS to give the corresponding peptide product.

The commercially available Rink amide AM resin (Chemimpex, loading: ~0.5 mmol/g) was employed in the solid phase peptide synthesis of peptides. The resin was swelled in DCM for 15 min and subjected to Fmoc deprotection by 20% piperidine DMF solution. After that, this resin was washed with dimethylformamide and dichloromethane. The resin was subjected to Fmoc-SPPS to give the corresponding peptide product.

The obtained resin bound peptide was treated with TFA cocktail for 1.5 h. After that, the peptide TFA solution was poured into 50 mL diethyl ether, and the resulting suspension was centrifuged to give a white pellet. After decanting diethyl ether, the remaining solid was ready for HPLC purification.

2.2 Using Cocktail A to release a C-terminal free and side-chain protected crude peptide acid from peptide bound resin.

After the standard Fmoc-SPPS, the peptide bound resin was washed with dichloromethane for three times. Subsequently, the resin was treated with cocktail A (CH_2Cl_2/AcOH/trifluoroethanol = 8/1/1) for 1 h. After that, the mixture was filtrated to give a clear solution bearing the desired peptidyl acid. This solution was co-evaporated with hexane for 6 times to give a white powder.

2.3 Synthesis of C-terminus '1' L-Amino acid salicylaldehyde semicarbazone ester hydrochloride (HCl·H2N-Xaa-CO-SALoff)

BocHN-Xaa(PG)-COOH, (1.0 equiv) was dissolved in DMF, followed by the addition of HATU (1.0 equiv) and DIPEA (2.0 equiv). The solution was stirred for 2 min, then salicylaldehyde semicarbazone (1.0 equiv) was added. The reaction mixture was stirred for overnight, after that, it was extracted with EtOAc and washed with brine. The organic layer was removed by reduce pressure evaporation, and the residue was purified by silica gel chromatography (CH_2Cl_2/EtOAc, 2:1) to give the desired BocHN-Xaa(PG)-CO-SAL^{off} product. This product was treated with a solution of HCl/dioxane (4 M) for 30 min, and the solvent was removed by co-evaporation under reduce pressure with toluene. Without purification, this salt was directly used in the n+1 reaction.

2.4 Synthesis of C-terminal Peptide SAL esters using n+1 strategy

The fully protected peptidyl acid (50 μmol, 1.0 equiv), obtained as described in the previous section **2.2**, was dissolved in $CHCl_3$ (15 mM,), then *N*-(3-dimethylaminopropyl)-*N*'-ethylcarbodiimide (EDC) (3.0 equiv) and hydroxy-3,4-dihydro-4-oxo-1,2,3-benzotriazine (HOOBt) (3.0 equiv) were added. After 5 min, the corresponding amino L-Amino acid salicylaldehyde semicarbazone ester hydrochloride

($HCl \cdot H_2N$-Xaa-CO-SAL^{off}) (3.0 equiv), obtained as described in the previous section, was added, and the reaction mixture was stirred for 3 h to form the crude protected C-terminal peptide SAL^{off} ester. This reaction mixture was subjected to TFA cocktail B (TFA/H_2O = 95:5, pyruvic acid = 100 equiv) deprotection, then the TFA solution was poured into a cold diethyl ether and followed by centrifugation to afford a white solid. After that, the diethyl ether layer was decanted, and the crude product was subjected to HPLC purification to afford the desired peptide SAL ester.

2.5 Preparation of hydrazine 2-chlorotrityl chloride resin

2-chlorotrityl chloride resin (1 g, loading = ~0.5 mmol/g) was swelled in 10 mL CH_2Cl_2/DMF (1/1, v/v). Then 10 mL $NH_2NH_2 \cdot H_2O$/DMF (1/20, v/v) was added. The reaction was conducted for 30 min. 10 mL of methanol/DMF (1/20, v/v) was added to quench the remaining 2-chlorotrityl chloride resin. After 30 min, the resin was washed with DMF and CH_2Cl_2 and ready for iterative peptide assembly (Fmoc-SPPS).

3. Synthesis of Interleukin-2 (IL-2) C-terminus

3.1 Synthesis of 4f

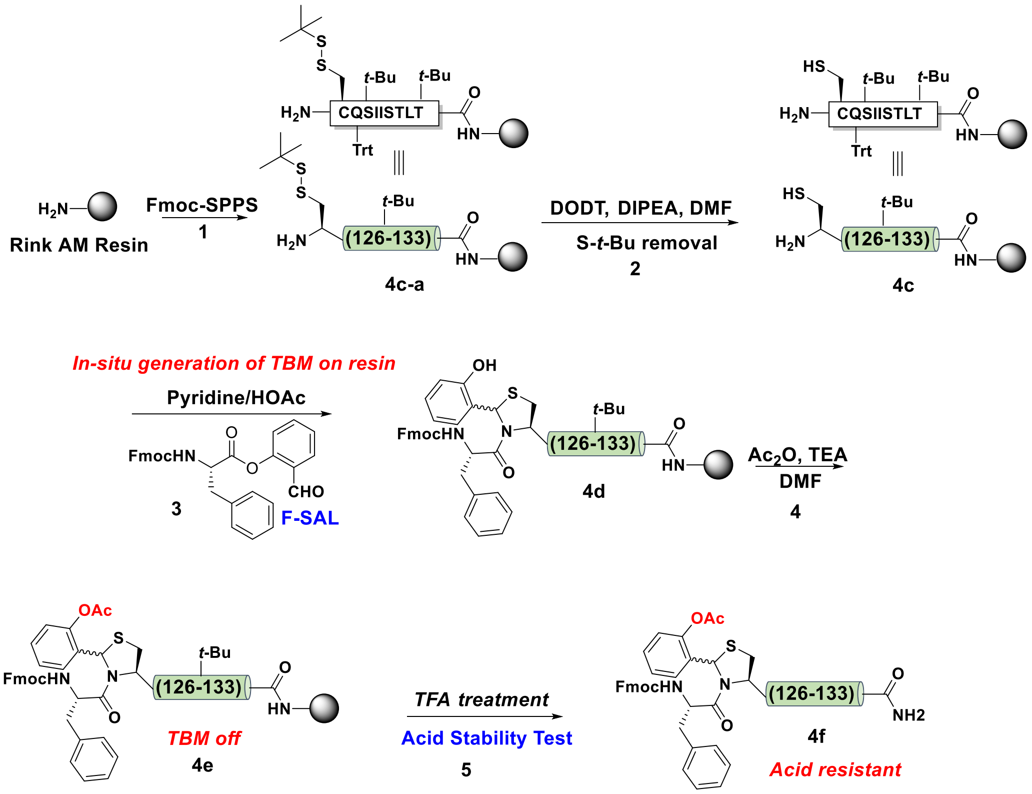

Figure 3.1-1 Synthesis of **4f**

Resin bound crude peptide **4c-a** was obtained by performing a general Fmoc-SPPS on 100 mg Rink amide resin (Chem-impex, loading: ~0.5 mmol/g, **Ser130-Thr131 pseudo-proline dipeptide**). Then, the resin was swelled in a deS-*t*-bu reagent (10.0 mL DMF, 1.0 mL DODT, 0.1 mL DIPEA) for overnight to provide the desired intermediate **4c**. Subsequently, the resin was washed by DMF and DCM. After that, 15 mL pyridine/HOAc buffer (1:1), containing SAL ester "**F-SAL**" (15 mM), was added to the above resin. The resulting mixture was reacted at r.t. for overnight to enable the on resin *in-situ* generation of TBM.

The resin bound protected peptide **4d** was subjected to Ac capping (10 mL DMF, 0.2 mL Ac_2O, 0.4 mL TEA, 40 min) to generate the desired product **4e** in its *TBM off* form. Subsequently, the resin was washed by DMF and DCM.

Then, an analytic TFA global deprotection (TFA/H_2O/TIPS = 95/2.5/2.5) was

performed to estimate the acid stability of *TBM off*. After TFA deprotection for 1 h, the desired product **4f** was observed, which indicated that *TBM off* could resist TFA treatment.

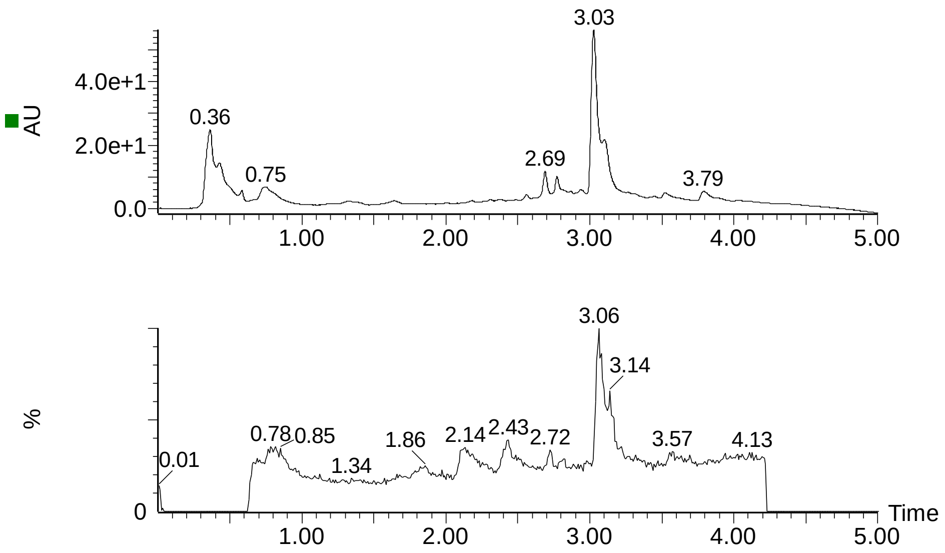

Figure 3.1-2 UV (190-400 nm) and MS (250-3000 m/z) trace from UPLC-MS analysis of **4f** gradient 40-95% CH_3CN/H_2O containing 0.1% TFA over 5 min at a flow rate of 0.4 mL/min.

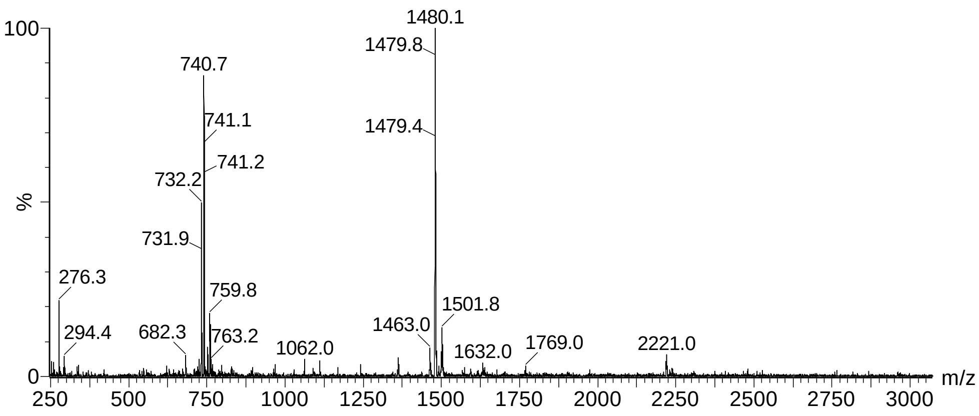

Figure 3.1-3 ESI-MS calcd. for $C_{73}H_{98}N_{12}O_{19}S$ $[M+1H]^{1+}$ m/z = 1480.7, found 1480.1; $[M+2H]^{2+}$ m/z = 740.8, found 740.7.

3.2 Synthesis of 4g

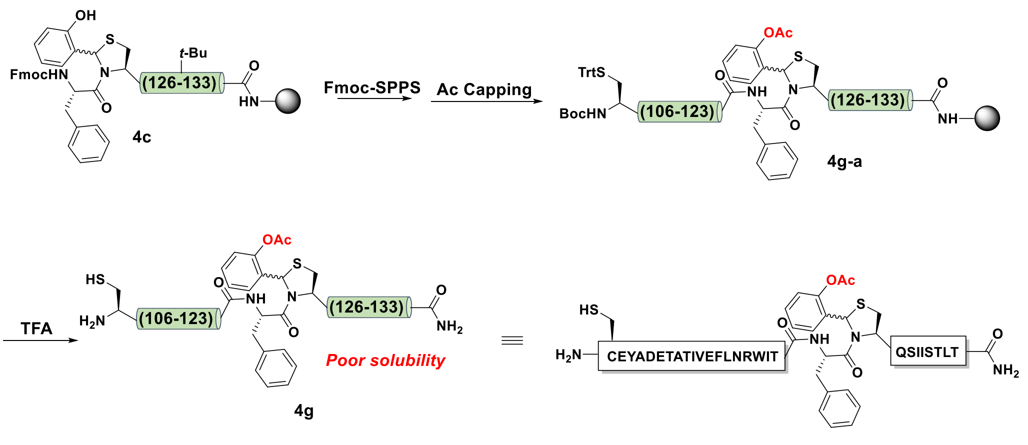

Figure 3.2-1 Synthesis of **4g**

Resin bound crude peptide **4c** was obtained from procedure **3.1**, then a general Fmoc-SPPS was performed to enable peptide elongation. After that, the resin was treated with 10 mL DMF containing 5% hydrazine monohydrate for 30 min, then, it was washed by DMF and DCM. The resin was treated with Ac capping reagent (10 mL DMF, 0.2 mL Ac_2O, 0.4 mL TEA, 40 min) to enable the synthesis of **4g**. Subsequently, the resin was subjected to TFA global deprotection (6 mL, TFA/TIPS/H_2O = 95:2.5:2.5) and cold diethyl ether precipitation, the resulting solid exhibited poor solubility in 50% ACN/H_2O and 8M GnHCl. (For analytic cleavage, after TFA deprotection, the peptide TFA solution was blow-off by N_2, then, it was dissolved by 50% ACN/H_2O and injected to UPLC-MS, immediately.)

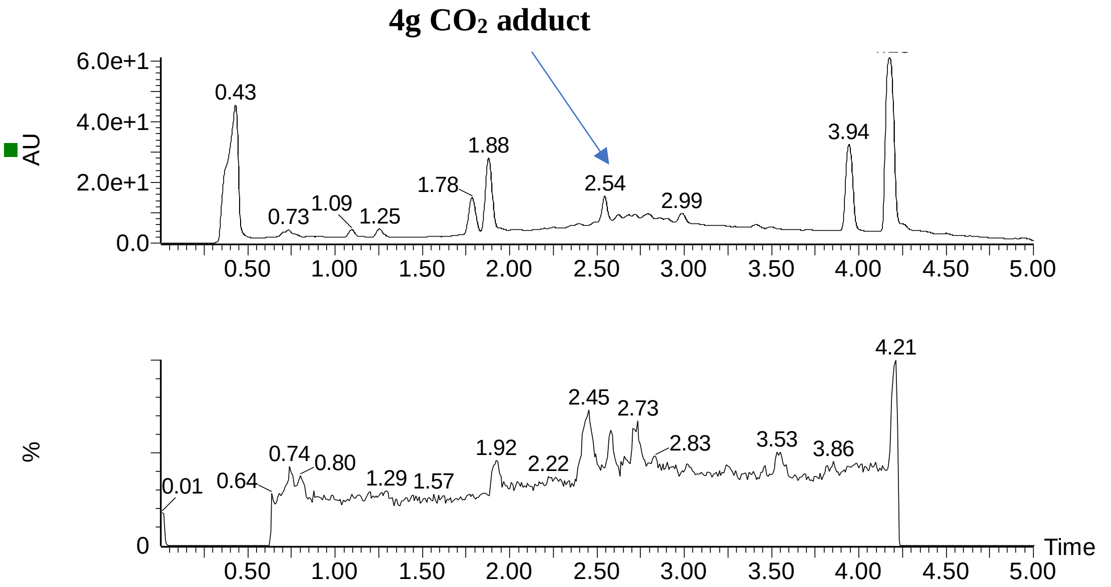

Figure 3.2-2 UV (190-400 nm) and MS (250-3000 m/z) trace from UPLC-MS analysis of **4g** gradient 40-95% CH_3CN/H_2O containing 0.1% TFA over 5 min at a flow rate of 0.4 mL/min.

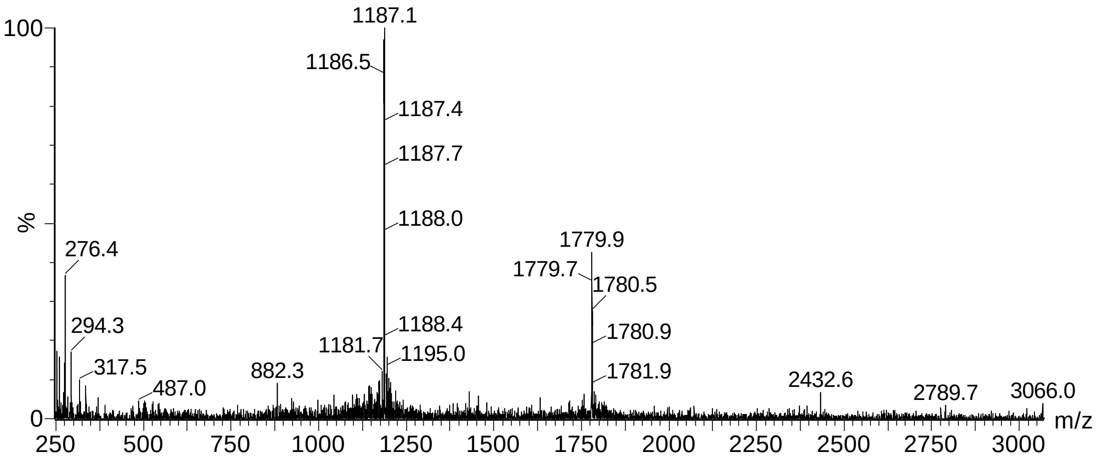

Figure 3.2-3 ESI-MS calcd. for $C_{161}H_{238}N_{36}O_{51}S_2$ $[M+2H]^{2+}$ m/z = 1780.0, found 1779.9; $[M+3H]^{3+}$ m/z = 1187.0, found 1187.1.

3.3 Synthesis of 4i

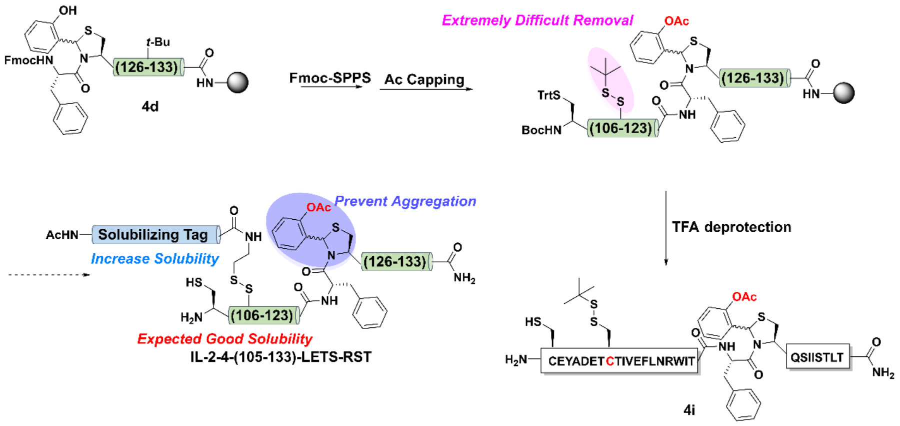

Figure 3.3-1 Synthesis of **4i**

Resin bound crude peptide **4d** was obtained from procedure **3.1**, then a general Fmoc-SPPS was performed to enable peptide elongation. After that, the resin was treated with 10 mL DMF containing 5% hydrazine monohydrate for 30 min, then, it was washed by DMF and DCM. The resin was treated with Ac capping reagent (10 mL DMF, 0.2 mL Ac_2O, 0.4 mL TEA, 40 min) to enable the synthesis of **4i**. Subsequently, the resin was subjected to deS-*t*-bu reagent (10.0 mL DMF, 1.0 mL DODT, 0.1 mL DIPEA). (For analytic cleavage, after TFA deprotection, the peptide TFA solution was blow-off by N_2, then, it was dissolved by 50% ACN/H_2O and injected to UPLC-MS, immediately.)

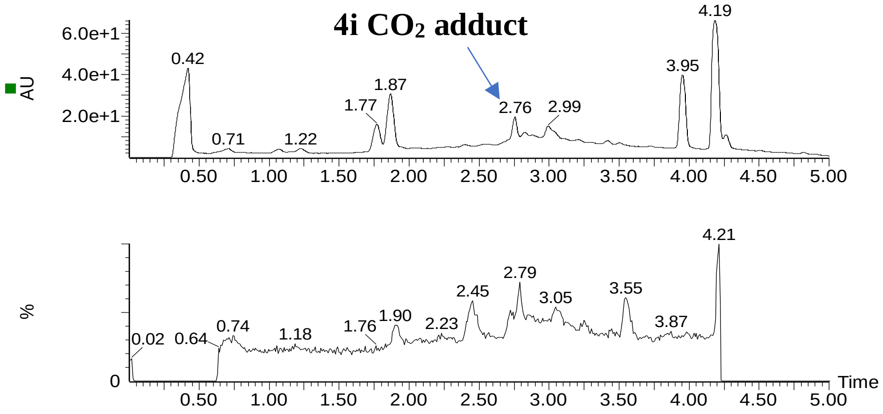

Figure 3.3-2 UV (190-400 nm) and MS (250-3000 m/z) trace from UPLC-MS analysis of **4i** gradient 40-95% CH_3CN/H_2O containing 0.1% TFA over 5 min at a flow rate of 0.4 mL/min.

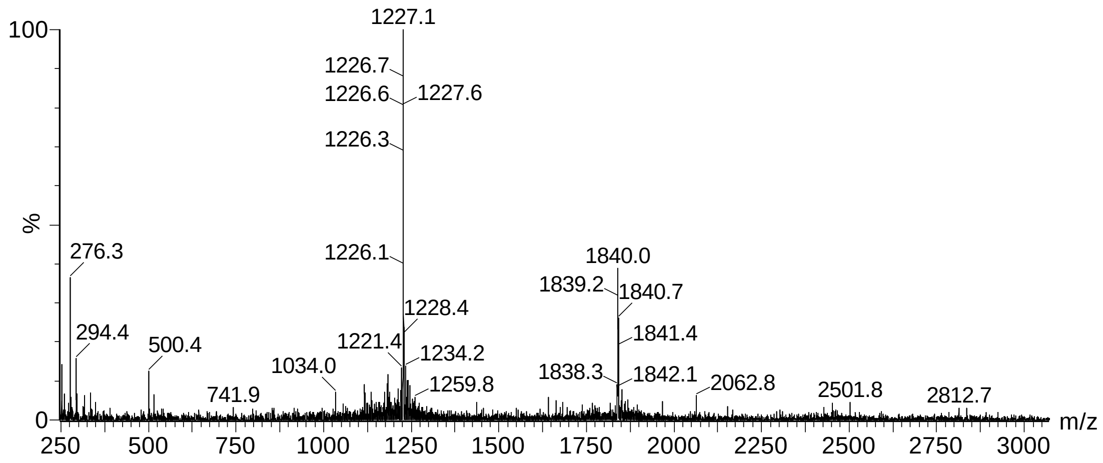

Figure 3.3-3 ESI-MS calcd. for $C_{165}H_{246}N_{36}O_{51}S_4$ $[M+2H]^{2+}$ m/z = 1840.1, found 1840.0; $[M+3H]^{3+}$ m/z = 1227.1, found 1227.1.

3.4 Synthesis of 4m

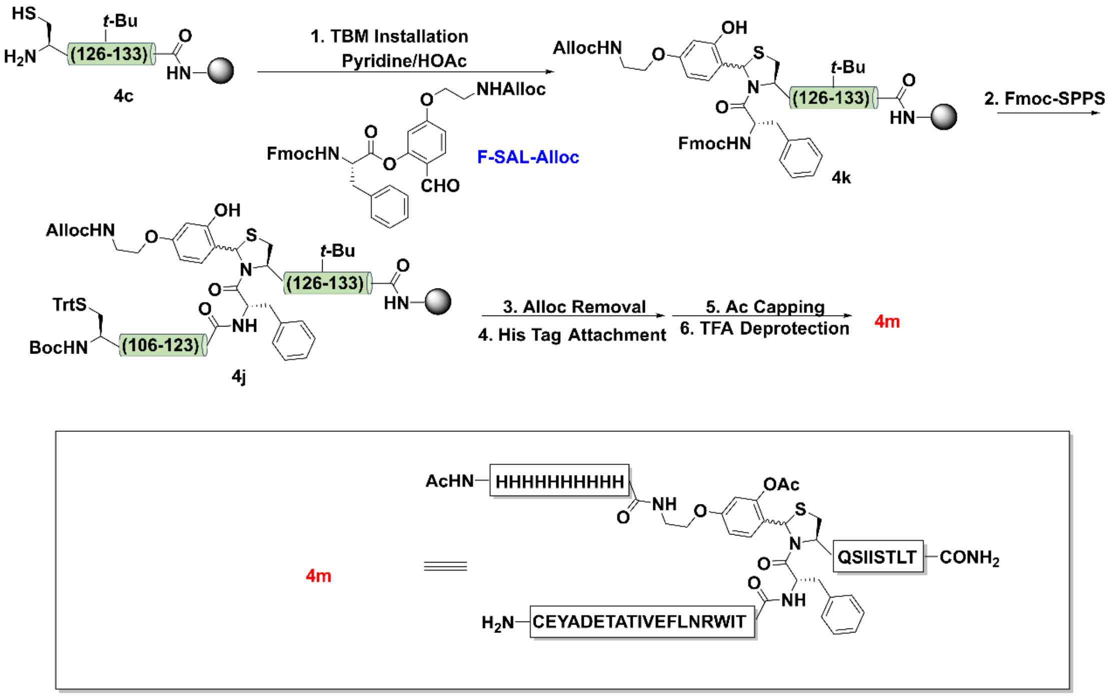

Figure 3.4-1 Synthesis of **IL-2-4-(105-133)-TBM-H$_{10}$-*off***

100 mg Rink Amide AM resin was employed in the synthesis of resin bound crude peptide **4c** by following synthetic procedure **3.1**. Subsequently, the resin was washed by DMF and DCM. After that, 15 mL pyridine/HOAc buffer (1:1), containing SAL ester "**F-SAL-Alloc**" (15 mM), was added to the above resin. The resulting mixture

was shaken at r.t. for overnight to enable the on resin *in-situ* generation of TBM.

Then a general Fmoc-SPPS was performed to enable peptide elongation. After that, the resin was treated with DeAlloc reagent (5 mL DMF, 50 mg $Pd(PPh_3)_4$, 100 µL $PhSiH_3$) for 1h to remove the Alloc protecting group. Next, a general Fmoc-SPPS was performed to enable the installation of His tag on the TBM.

After the peptide elongation, the resin was treated with 10 mL DMF containing 5% hydrazine monohydrate for 30 min, then, it was washed by DMF and DCM. The resin was treated with Ac capping reagent (10 mL DMF, 0.2 mL Ac_2O, 0.4 mL TEA, 40 min) to enable the synthesis of **4m**. Subsequently, the resin was subjected to DCM washing, TFA global deprotection (6 mL, TFA/TIPS/H_2O = 95:2.5:2.5) and cold diethyl ether precipitation, the resulting solid was subjected to HPLC purification (15-60% CH_3CN/H_2O over 30 min) and lyophilization, the desired peptide **4m** was obtained as a white powder (29.8 mg, 12 %).

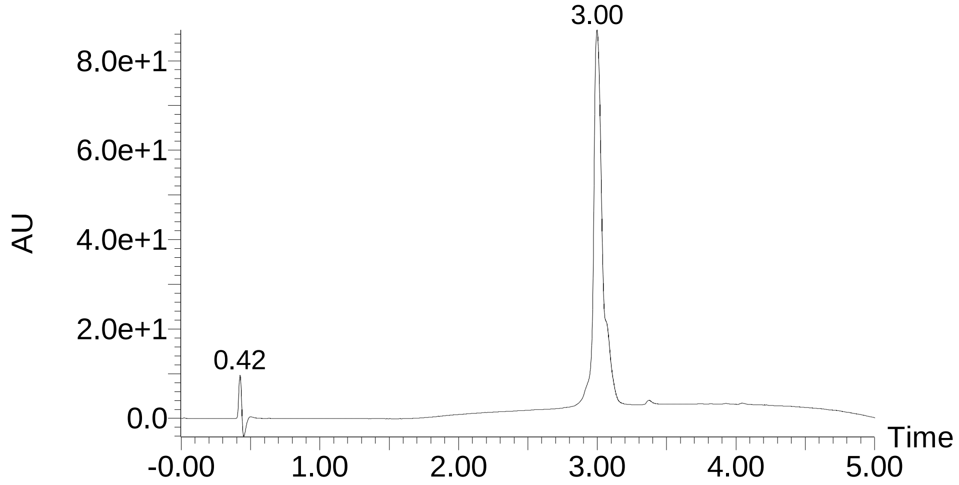

Figure 3.4-2 UV (190-400 nm) and MS (250-3000 m/z) trace from UPLC-MS analysis of **4m** gradient 5-95% CH_3CN/H_2O containing 0.1% TFA over 5 min at a flow rate of 0.4 mL/min.

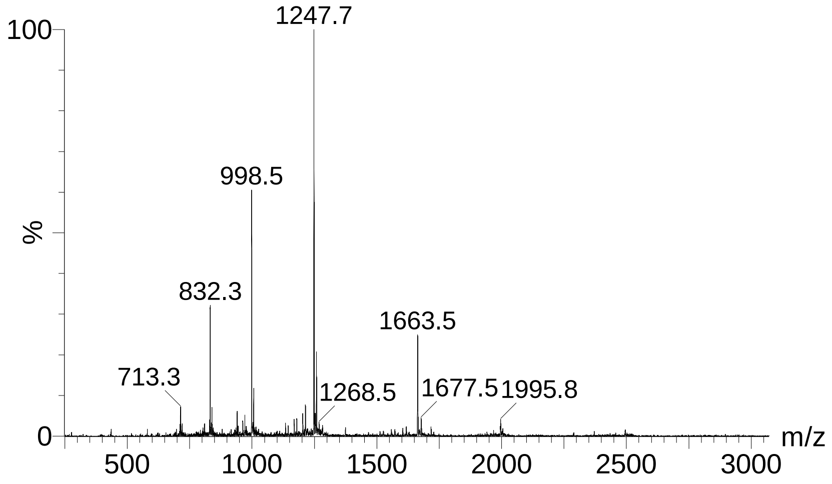

Figure 3.4-3 ESI-MS calcd. for $C_{224}H_{315}N_{67}O_{61}S_2$ $[2M+5H]^{5+}$ m/z = 1995.6, found 1995.8; $[M+3H]^{3+}$ m/z = 1663.2, found 1663.5; $[M+4H]^{4+}$ m/z = 1247.6, found 1247.7; $[M+5H]^{5+}$ m/z = 998.3, found 998.5; $[M+6H]^{6+}$ m/z = 832.1, found 832.3; $[M+7H]^{7+}$ m/z = 713.4, found 713.3.

4. The First Synthetic Attempt of Interleukin-2 (IL-2)

4.1 Synthesis of peptide fragment 1

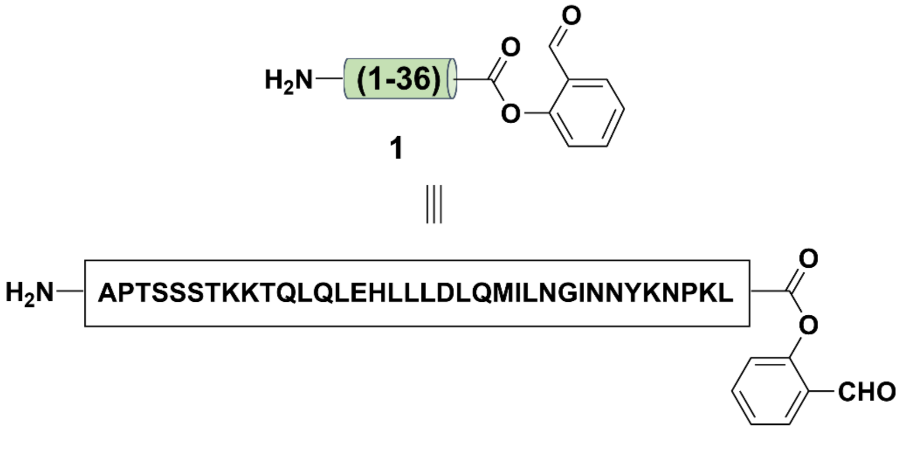

Figure 4.1-1 Peptide fragment **1**

IL-2-1-(1-35) was synthesized by general procedure **2.1** using 100 mg 2-choloride trityl resin (loading 0.5 mmol/g). Subsequently, following procedure **2.2**, **2.4**, TFA global deprotection (10 mL, TFA/H_2O = 95:5, 100 equiv pyruvic acid), cold diethyl

ether precipitation, HPLC purification (15-60% CH_3CN/H_2O over 30 min) and lyophilization, the desired peptide SAL ester **1** was obtained as a white powder (64.6 mg, 31 %).

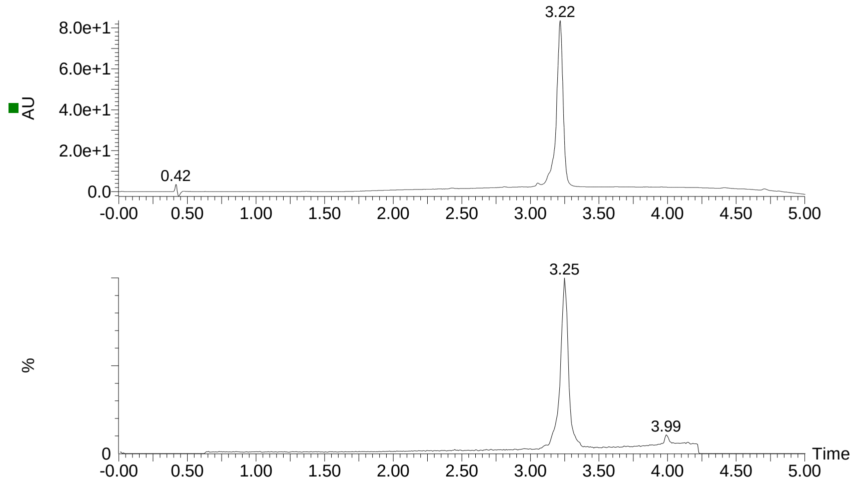

Figure 4.1-2 UV (190-400 nm) and MS (250-3000 m/z) trace from UPLC-MS analysis of **1** gradient 5-95% CH_3CN/H_2O containing 0.1% TFA over 5 min at a flow rate of 0.4 mL/min.

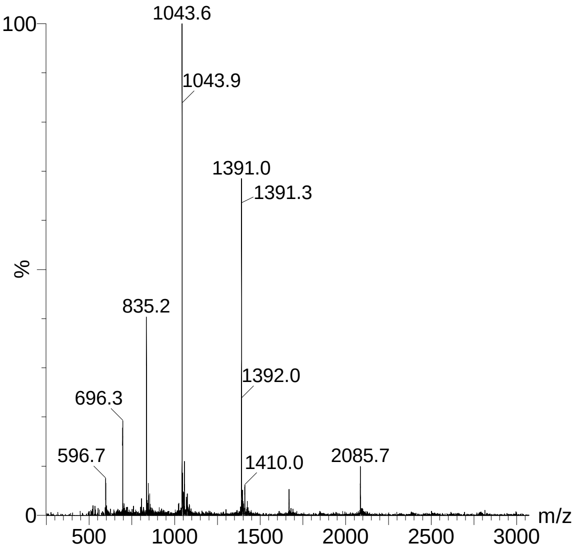

Figure 4.1-3 ESI-MS calcd. for $C_{187}H_{307}N_{49}O_{56}S$ $[M+2H]^{2+}$ m/z = 2085.9, found 2085.7; $[M+3H]^{3+}$ m/z = 1391.0, found 1391.0; $[M+4H]^{4+}$ m/z = 1043.5, found 1043.6; $[M+5H]^{5+}$ m/z = 835.0, found 835.2; $[M+6H]^{6+}$ m/z = 696.0, found 696.3; $[M+7H]^{7+}$ m/z = 596.7, found 596.7.

4.2 Synthesis of peptide fragment 2

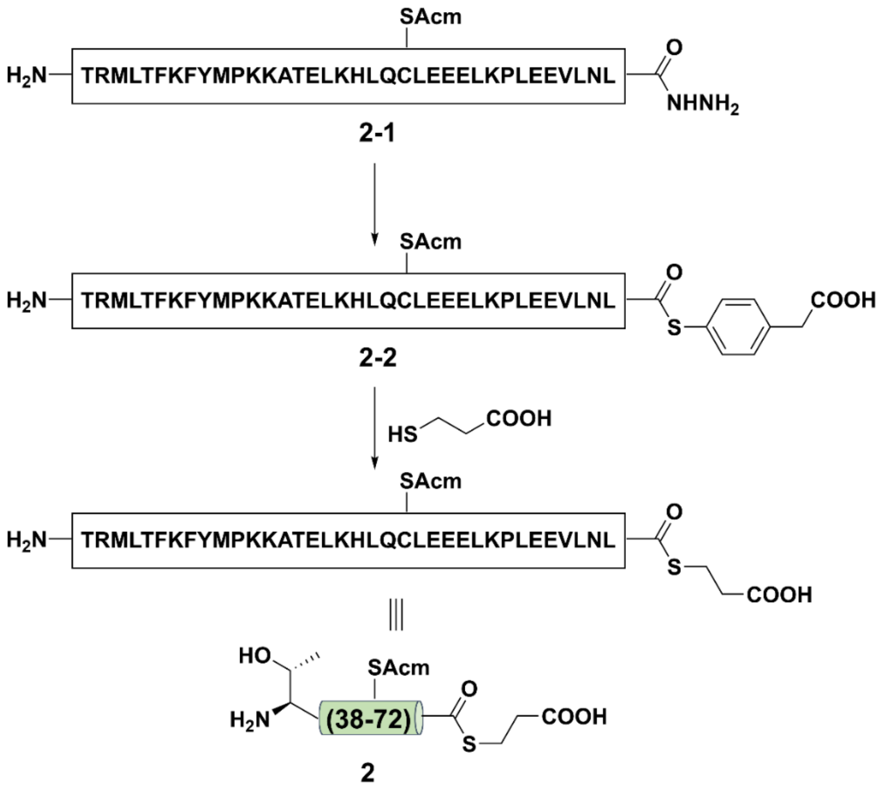

Figure 4.2-1 Synthesis of Peptide fragment **2**

2-1 was synthesized by general procedure **2.1** using 100 mg hydrazine 2-chlorotrityl chloride resin (loading 0.5 mmol/g). Subsequently, TFA global deprotection (6 mL, TFA/TIPS/H_2O = 95:2.5:2.5) and cold diethyl ether precipitation, the resulting solid was dissolved in 10 mL 50% ACN/H_2O. This peptide containing solution was subjected to HPLC purification (15-60% CH_3CN/H_2O over 30 min) and lyophilization, and the desired **2-1** was obtained as a white powder (77.6 mg, 35%).

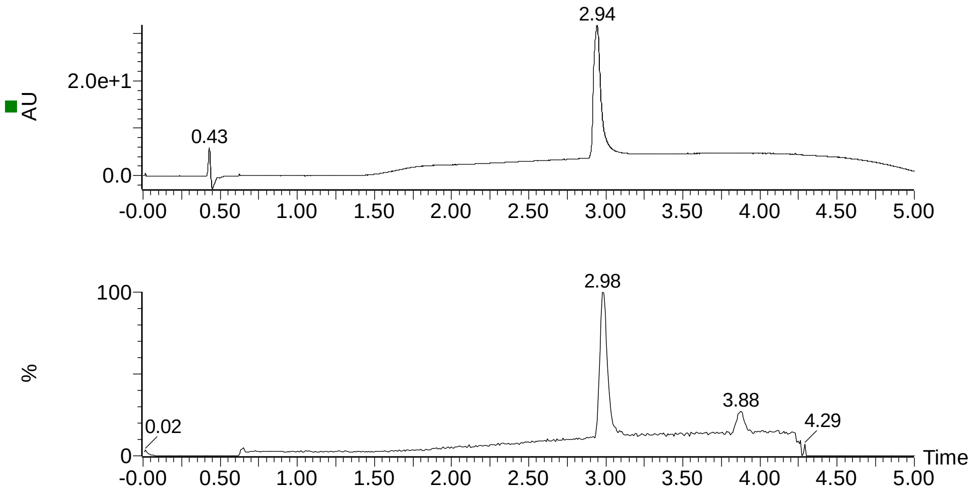

Figure 4.2-2 UV (190-400 nm) and MS (250-3000 m/z) trace from UPLC-MS analysis of **2-1** gradient 5-95% CH_3CN/H_2O containing 0.1% TFA over 5 min at a flow rate of 0.4 mL/min.

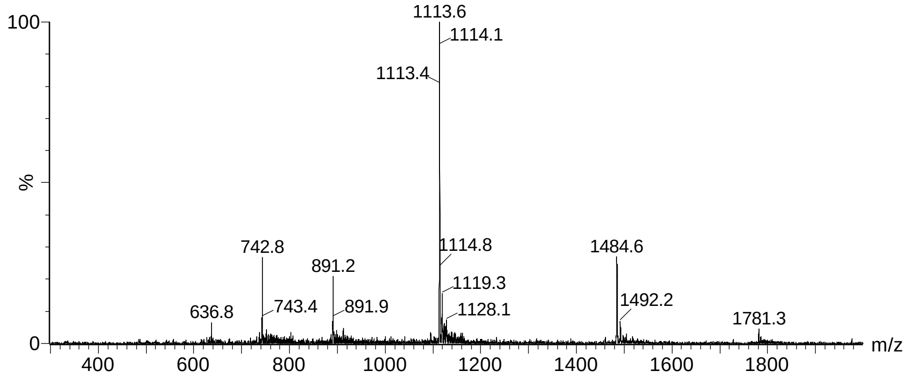

Figure 4.2-3 ESI-MS calcd. for $C_{202}H_{331}N_{51}O_{55}S_3$ $[2M+5H]^{5+}$ m/z = 1781.1, found 1781.3; $[M+3H]^{3+}$ m/z = 1484.5, found 1484.6; $[M+4H]^{4+}$ m/z = 1113.6, found 1113.6; $[M+5H]^{5+}$ m/z = 891.1, found 891.2; $[M+6H]^{6+}$ m/z = 742.7, found 742.8; $[M+7H]^{7+}$ m/z = 636.8, found 636.8.

Peptide **2-1** (22.3 mg, 5.0 μmol, 1.0 equiv) was dissolved in 2.0 mL aqueous buffer containing 6M GnHCl and 0.2 M NaH_2PO_4 (pH 3.0), and cooled to -15 °C in an ice-salt bath. 30 μL of 0.5 M $NaNO_2$ was added to activate the peptide hydrazide. After stirred at -15 °C for 15 min, 4-mercaptophenylacetic acid (MPAA, 25.2 mg, 150.0 μmol, 30.0 equiv) was dissolved in 1.0 mL 0.2 M NaH_2PO_4 solution containing 6 M GnHCl (pH 7.0), and the solution was added into the above mixture. The reaction mixture was stirred for 30 min at r.t., then 3-mercaptopropanoic acid (26.5 mg, 250.0 μmol, 50.0 equiv) was added. The pH value of reaction mixture was adjusted to 6.5 slowly with aqueous NaOH solution (1 M) to enable the formation of **2**. After overnight reaction, the mixture was treated with TCEP, then extracted with ethyl acetate to remove the MPAA. The resulting mixture was purified by preparative HPLC (15-60% CH_3CN/H_2O over 30 min), followed by lyophilization to afford product **2** as a white solid (12.5 mg, 55%).

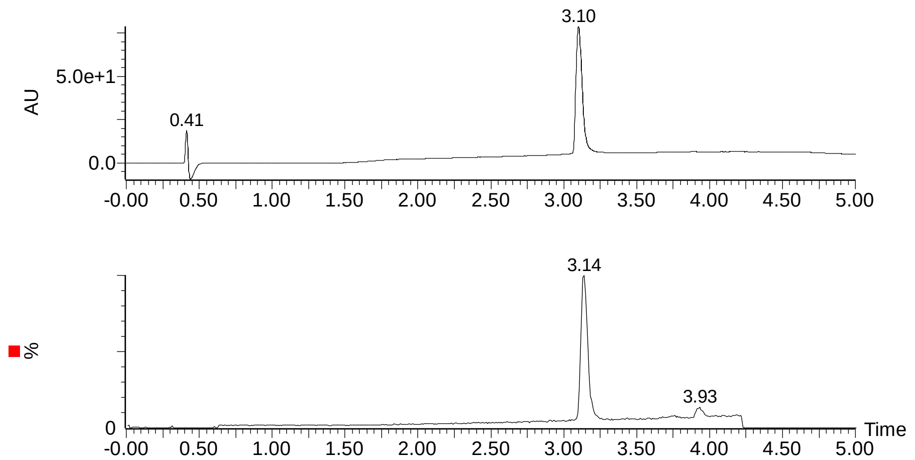

Figure 4.2-4 UV (190-400 nm) and MS (250-3000 m/z) trace from UPLC-MS analysis of **2** gradient 5-95% CH_3CN/H_2O containing 0.1% TFA over 5 min at a flow rate of 0.4 mL/min.

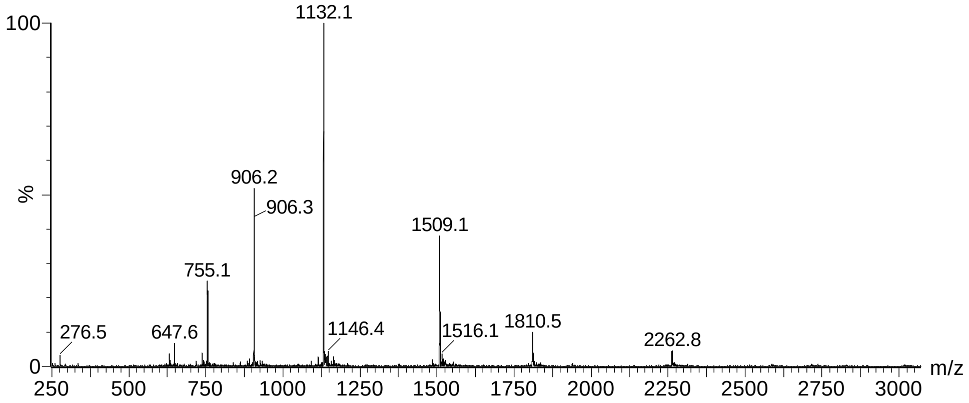

Figure 4.2-5 ESI-MS calcd. for $C_{205}H_{333}N_{49}O_{57}S_4$ $[M+2H]^{2+}$ m/z = 2263.2, found 2262.8; $[2M+5H]^{5+}$ m/z = 1810.8, found 1810.5; $[M+3H]^{3+}$ m/z = 1509.1, found 1509.1; $[M+4H]^{4+}$ m/z = 1132.1, found 1132.1; $[M+5H]^{5+}$ m/z = 905.9, found 906.2; $[M+6H]^{6+}$ m/z = 755.1, found 755.1; $[M+7H]^{7+}$ m/z = 647.3, found 647.6.

4.3 Synthesis of peptide fragment 3

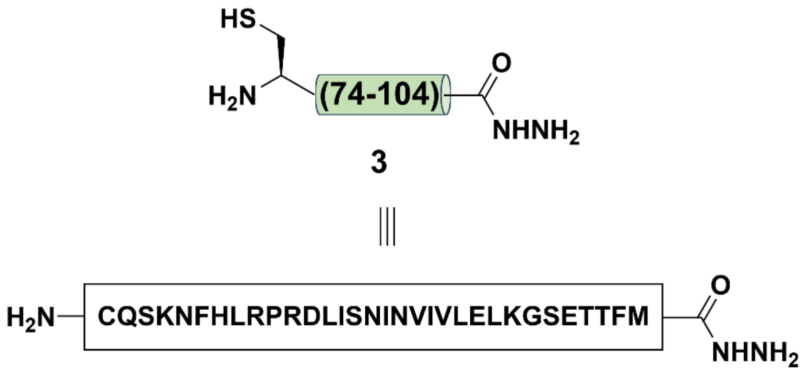

3

Figure 4.3-1 Peptide Fragment 3

3 was synthesized by general procedure **2.1** using 100 mg hydrazine 2-chlorotrityl chloride resin (loading 0.5 mmol/g), and Gly-HMB was employed in position 98 to enable the synthesis. Subsequently, TFA global deprotection (6 mL, TFA/TIPS/H_2O = 95:2.5:2.5) and cold diethyl ether precipitation. The resulting solid was dissolved in 1 mL DMSO, which was diluted by 5 mL 50% ACN/H_2O and subjected to HPLC purification, immediately (20-60% CH_3CN/H_2O over 30 min). After lyophilization, the desired **3** was obtained as a white powder (16.7 mg, 9%).

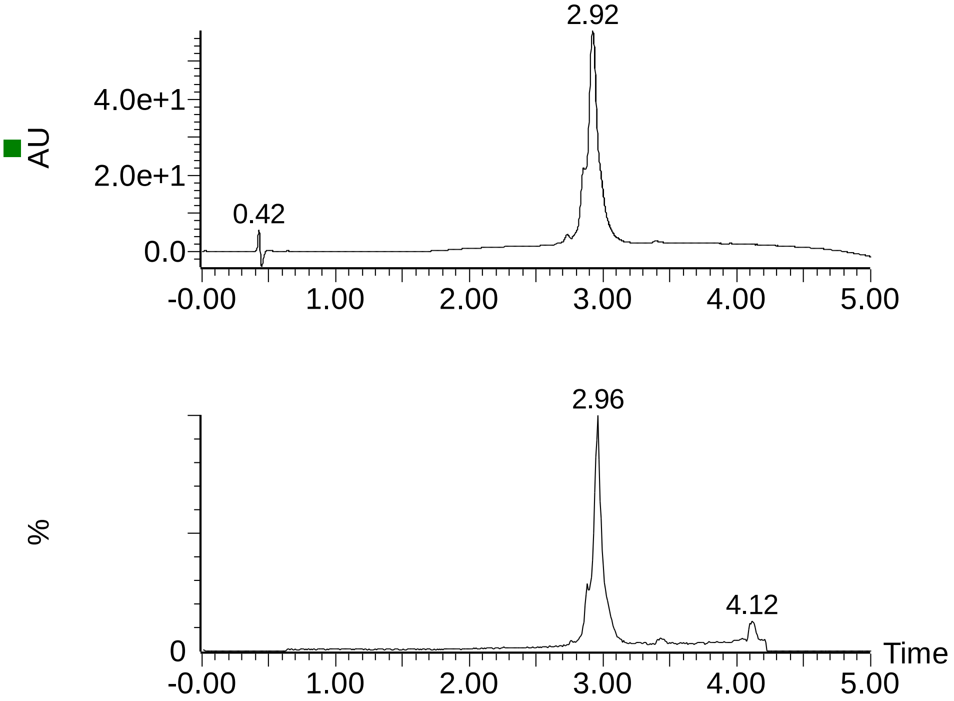

Figure 4.3-2 UV (190-400 nm) and MS (250-3000 m/z) trace from UPLC-MS analysis of **3** gradient 5-95% CH_3CN/H_2O containing 0.1% TFA over 5 min at a flow rate of 0.4 mL/min.

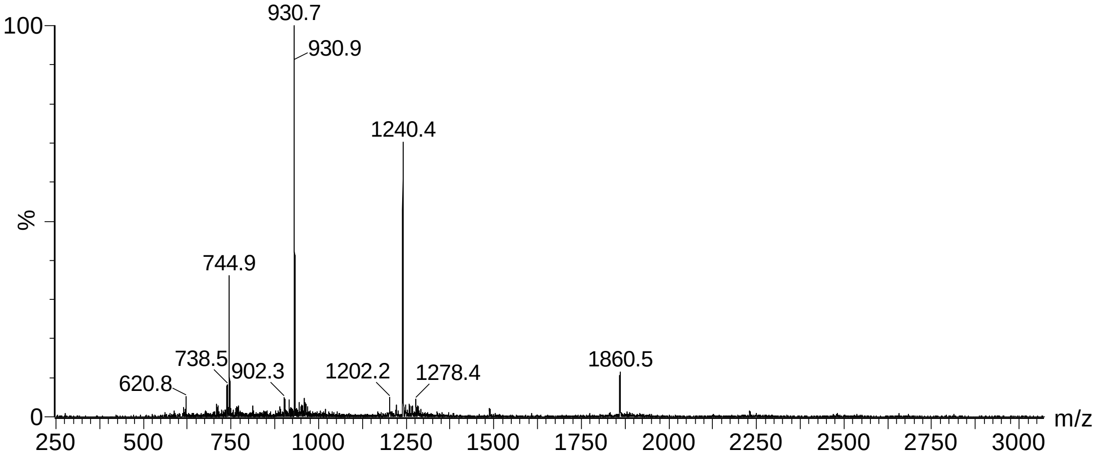

Figure 4.3-3 ESI-MS calcd. for $C_{163}H_{270}N_{48}O_{47}S_2$ $[M+2H]^{2+}$ m/z = 1860.2, found 1860.5; $[M+3H]^{3+}$ m/z = 1240.5, found 1240.4; $[M+4H]^{4+}$ m/z = 930.6, found 930.7; $[M+5H]^{5+}$ m/z = 744.7, found 744.9; $[M+6H]^{6+}$ m/z = 620.7, found 620.8.

4.4 Synthesis of peptide fragment 5

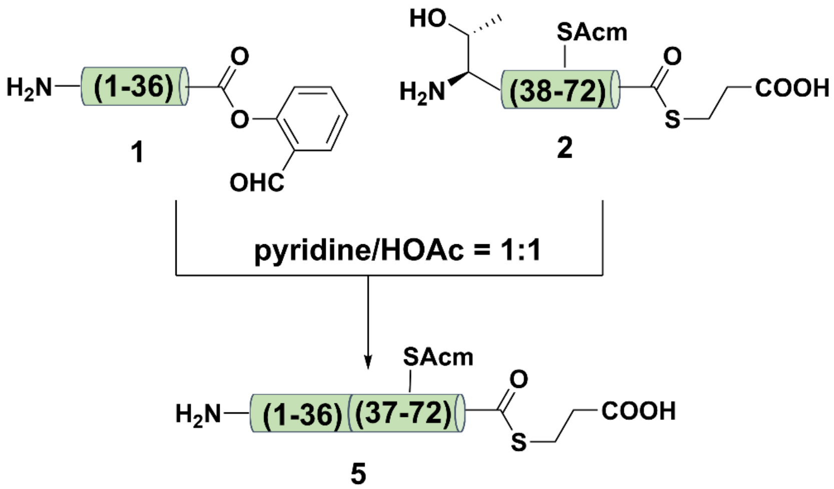

Figure 4.4-1 Synthesis of **IL-2-12-(1-72)**

1 (10.0 mg, 2.4 µmol) and **2** (13.1 mg, 2.9 µmol) were dissolved in pyridine/acetic acid (1/1, mol/mol) buffer at a concentration of 10 mM under room temperature. The reaction mixture was stirred at room temperature for 6 h. After completion of the reaction, the solvent was blown off under a stream of condensed N_2. The residue was treated with 3.0 mL of TFA/H_2O (95/5, v/v) for 30 min. After that, the TFA was blown off under a stream of condensed N_2 and treated with cold diethyl ether to give a white suspension for centrifugation.

After decanting diethyl ether, the remaining solid was dissolved in 50% ACN/H_2O, then it was filtrated by Syringe Filters (PTFE 0.22µm) and subjected to preparative HPLC purification (15-60% CH_3CN/H_2O over 30 min) and lyophilization to give 7.6 mg (37 % yield) of peptide **5** as a white powder.

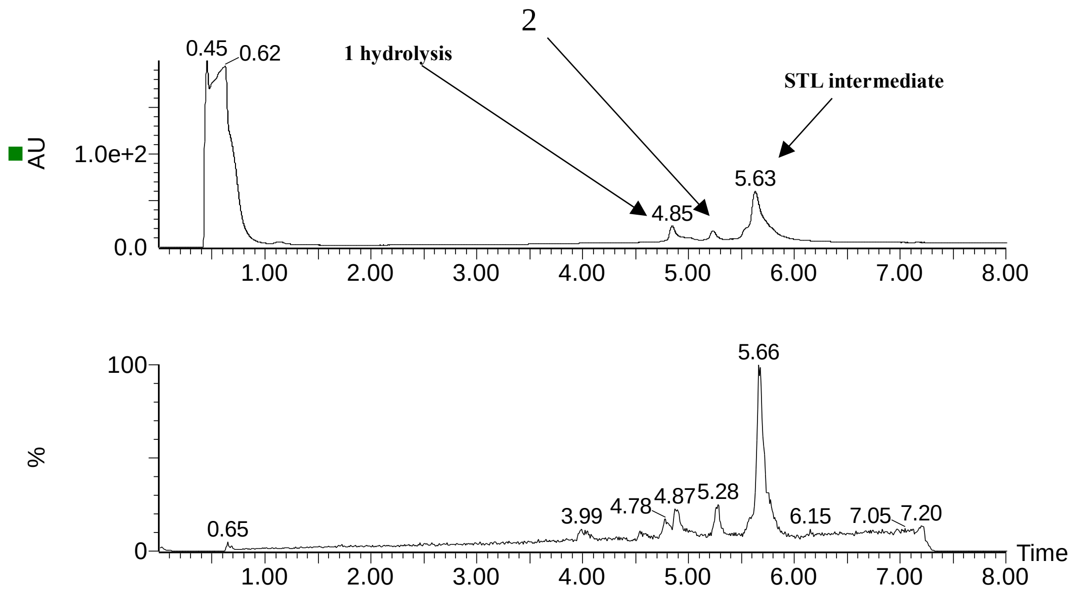

Figure 4.4-2 UV (190-400 nm) and MS (250-3000 m/z) trace from UPLC-MS analysis of **STL** between **1** and **2** gradient 15-60% CH_3CN/H_2O containing 0.1% TFA over 8 min at a flow rate of 0.4 mL/min.

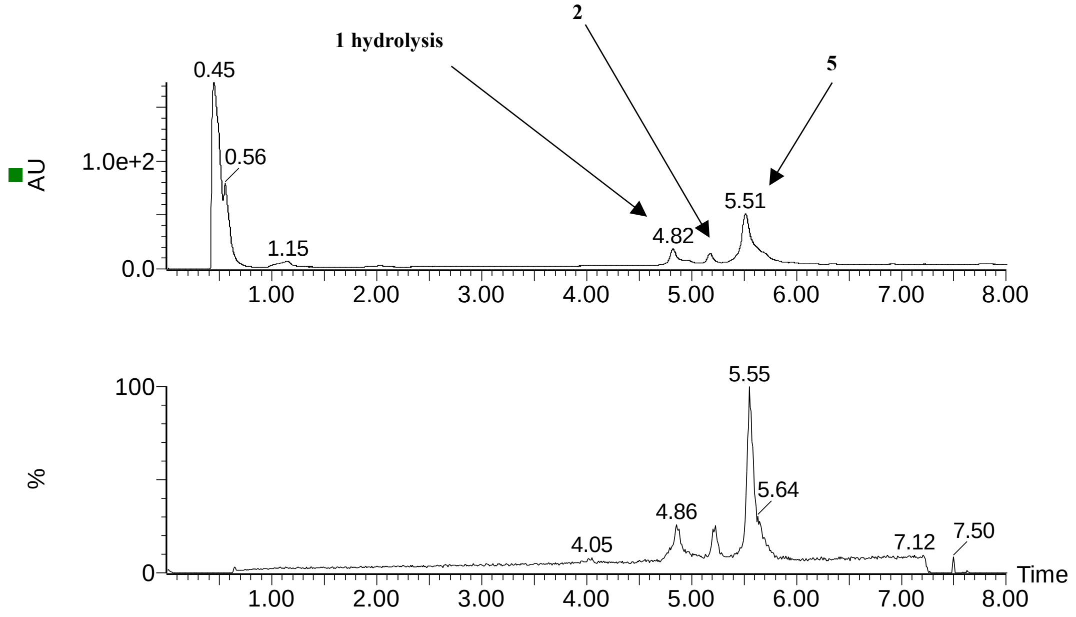

Figure 4.4-3 UV (190-400 nm) and MS (250-3000 m/z) trace from UPLC-MS analysis of **STL Acidolysis** between **1** and **2** gradient 15-60% CH_3CN/H_2O containing 0.1% TFA over 8 min at a flow rate of 0.4 mL/min.

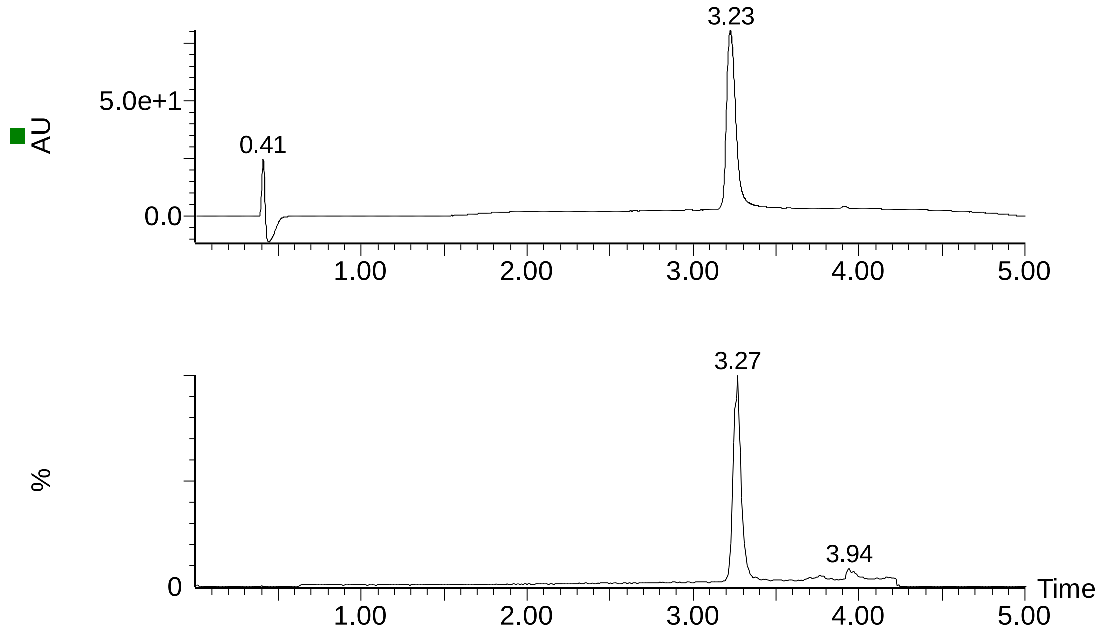

Figure 4.4-4 UV (190-400 nm) and MS (250-3000 m/z) trace from UPLC-MS analysis of **purified 5** gradient 5-95% CH_3CN/H_2O containing 0.1% TFA over 5 min at a flow rate of 0.4 mL/min.

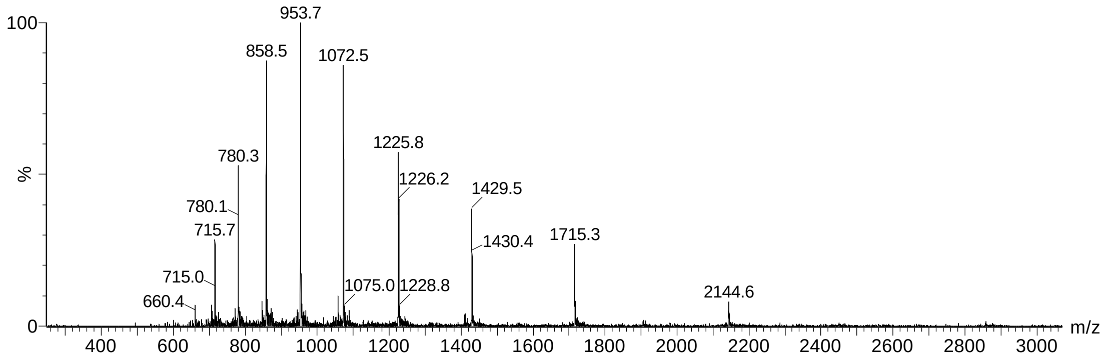

Figure 4.4-5 ESI-MS calcd. for $C_{385}H_{634}N_{98}O_{111}S_5$ $[M+4H]^{4+}$ m/z = 2144.1, found 2144.6; $[M+5H]^{5+}$ m/z = 1715.4, found 1715.3; $[M+6H]^{6+}$ m/z = 1429.7, found 1429.5; $[M+7H]^{7+}$ m/z = 1225.6, found 1225.8; $[M+8H]^{8+}$ m/z = 1072.5, found 1072.5; $[M+9H]^{9+}$ m/z = 953.5, found 953.7; $[M+10H]^{10+}$ m/z = 858.2, found 858.5; $[M+11H]^{11+}$ m/z = 780.3, found 780.3; $[M+12H]^{12+}$ m/z = 715.4, found 715.7; $[M+13H]^{13+}$ m/z = 660.4, found 660.4.

5. The Second Synthetic Attempt of Interleukin-2 (IL-2)

5.1 Synthesis of peptide fragment 2a

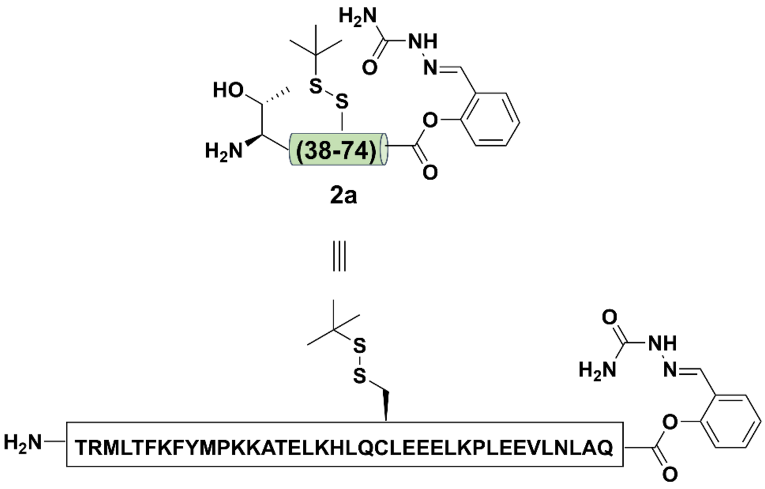

Figure 5.1-1 Peptide fragment **2a**

IL-2-2-(37-73) was synthesized by general procedure **2.1** using 100 mg 2-choloride trityl resin (loading 0.5 mmol/g). Subsequently, following procedure **2.2**, **2.4**, TFA global deprotection (10 mL, TFA/H_2O = 95:5) and cold diethyl ether precipitation, the resulting solid was dissolved in 10 mL 50% ACN/H_2O. After that, semicarbazide hydrochloride (5 equiv) was added and reacted for 30 min. This reaction mixture was subjected to HPLC purification (15-60% CH_3CN/H_2O over 30 min) and lyophilization, and the desired peptide SAL ester **2a** was obtained as a white powder (79.4 mg, 33%).

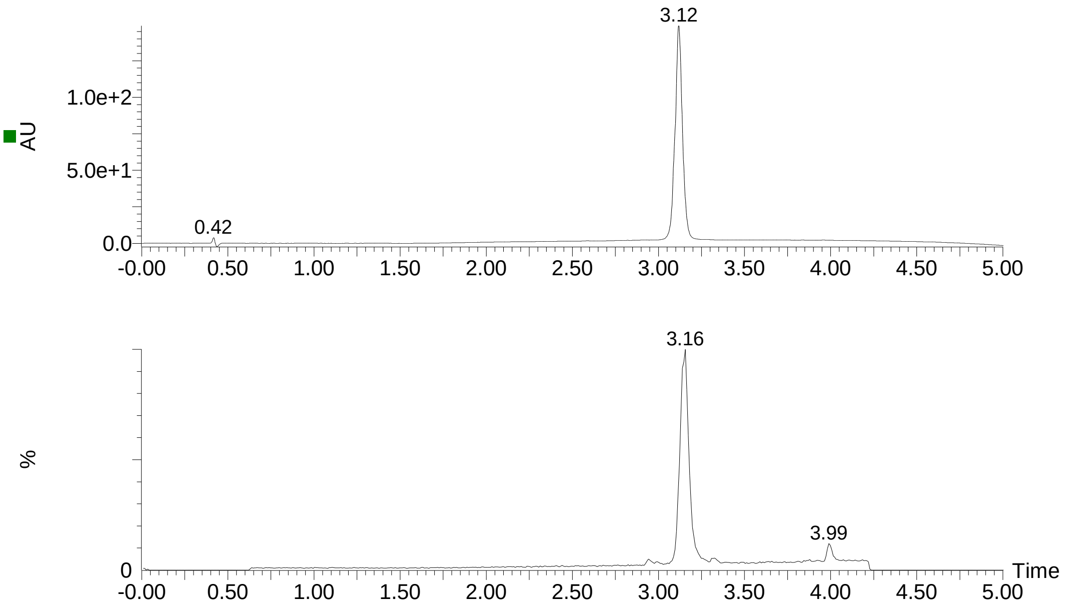

Figure 5.1-2 UV (190-400 nm) and MS (250-3000 m/z) trace from UPLC-MS analysis of **2a** gradient 5-95% CH_3CN/H_2O containing 0.1% TFA over 5 min at a flow rate of 0.4 mL/min.

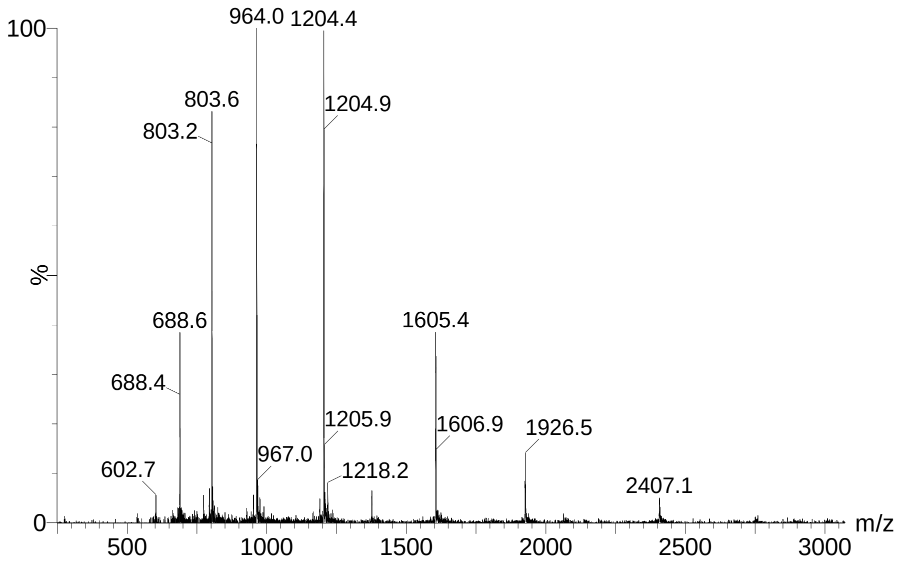

Figure 5.1-3 ESI-MS calcd. for $C_{219}H_{352}N_{54}O_{59}S_4$ $[M+2H]^{2+}$ m/z = 2407.9, found 2407.1; $[2M+5H]^{5+}$ m/z = 1926.5, found 1926.5; $[M+3H]^{3+}$ m/z = 1605.6, found 1605.4; $[M+4H]^{4+}$ m/z = 1204.5, found 1204.4; $[M+5H]^{5+}$ m/z = 963.8, found 964.0; $[M+6H]^{6+}$ m/z = 803.3, found 803.6; $[M+7H]^{7+}$ m/z = 688.7, found 688.6; $[M+8H]^{8+}$ m/z = 602.7, found 602.7.

5.2 Synthesis of peptide fragment 3a

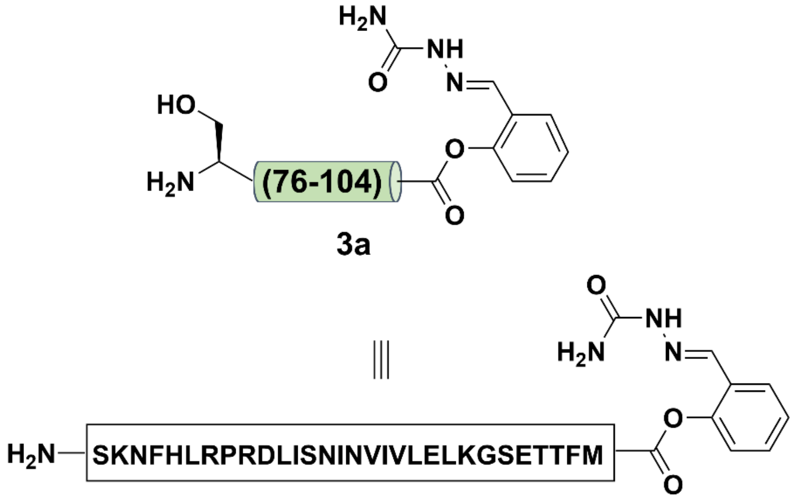

Figure 5.2-1 Peptide fragment **3a**

IL-2-3-(75-103) was synthesized by general procedure **2.1** using 100 mg 2-choloride trityl resin (loading 0.5 mmol/g) (HMB was employed in Gly98). Subsequently, following procedure **2.2**, **2.4**, TFA global deprotection (10 mL, TFA/H_2O = 95:5) and cold diethyl ether precipitation, the resulting solid was dissolved in 10 mL 50% ACN/H_2O. After that, semicarbazide hydrochloride (5 equiv) was added and reacted for 30 min. This reaction mixture was subjected to HPLC purification (15-60% CH_3CN/H_2O over 30 min) and lyophilization, and the desired peptide SAL ester **3a** was obtained as a white powder (12.7 mg, 7%).

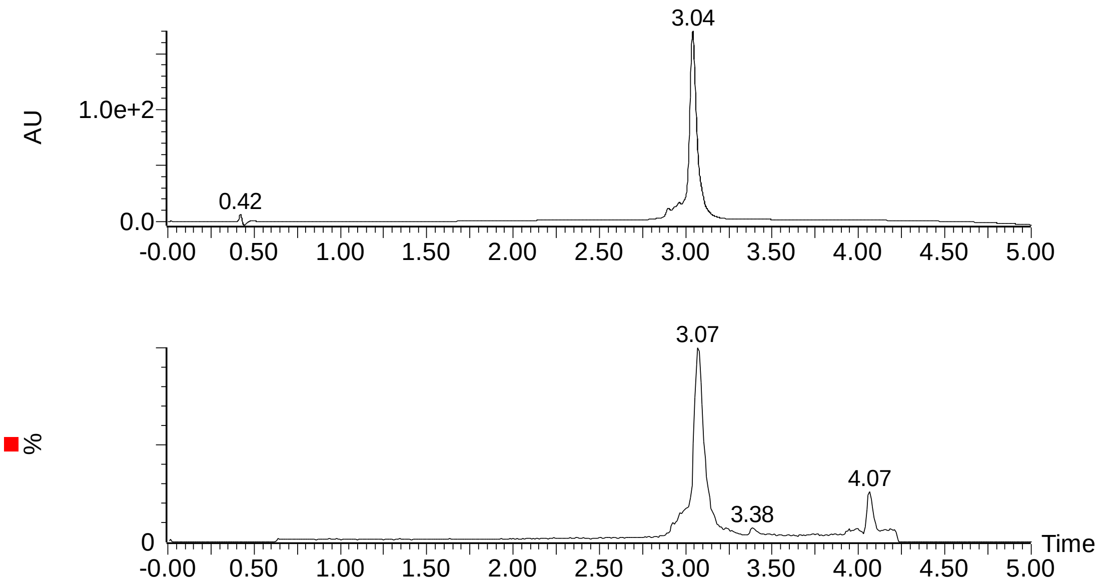

Figure 5.2-2 UV (190-400 nm) and MS (250-3000 m/z) trace from UPLC-MS analysis of **3a** gradient 5-95% CH_3CN/H_2O containing 0.1% TFA over 5 min at a flow rate of 0.4 mL/min.

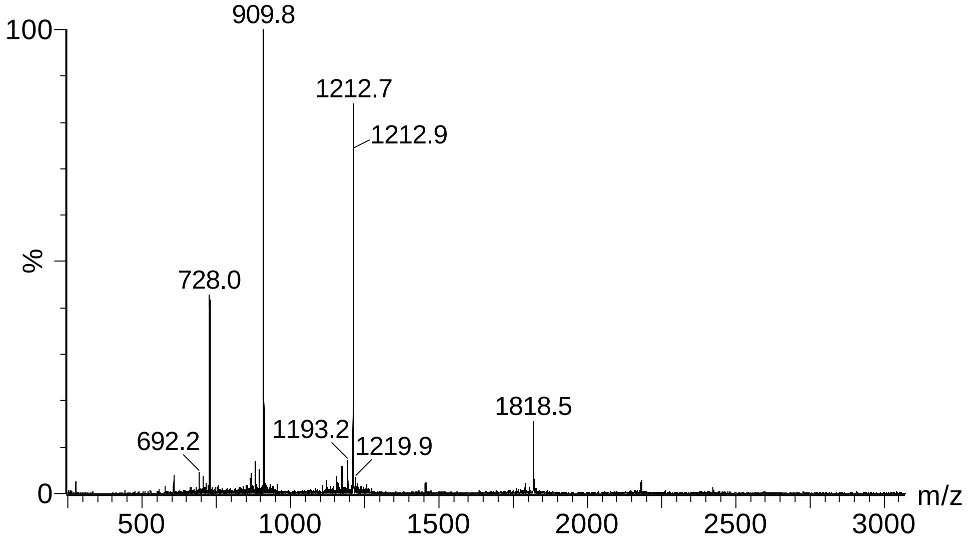

Figure 5.2-3 ESI-MS calcd. for $C_{163}H_{262}N_{46}O_{46}S$ $[M+2H]^{2+}$ m/z = 1818.1, found 1818.5; $[M+3H]^{3+}$ m/z = 1212.4, found 1212.7; $[M+4H]^{4+}$ m/z = 909.6, found 909.8; $[M+5H]^{5+}$ m/z = 727.8, found 728.0.

5.3 Synthesis of peptide fragment 5a

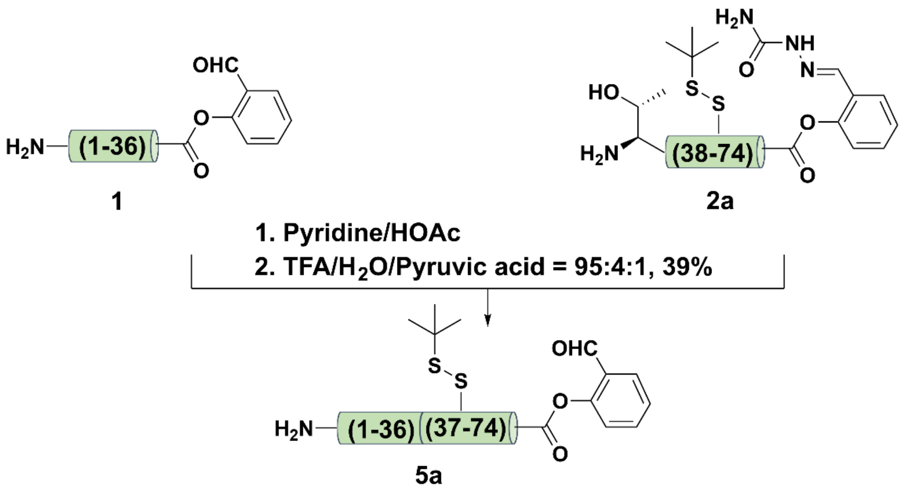

Figure 5.3-1 Synthetic scheme of **5a**

1 (20.0 mg, 4.8 μmol) and **2a** (27.9 mg, 5.8 μmol) were dissolved in pyridine/acetic acid (1/1, mol/mol) buffer at a concentration of 10 mM under room temperature. The reaction mixture was stirred at room temperature for 6 h. After completion of the reaction, the solvent was blown off under a stream of condensed N_2. The residue was treated with 3.0 mL of TFA/H_2O/Pyruvic acid (95/4/1, v/v/v) for 30 min. After that, the TFA was blown off under a stream of condensed N_2 and treated with cold diethyl ether to give a white suspension for centrifugation.

After decanting diethyl ether, the remaining solid was dissolved in 50% ACN/H_2O, then it was filtrated by Syringe Filters (PTFE 0.22μm) and subjected to preparative HPLC purification (15-60% CH_3CN/H_2O over 30 min) and lyophilization to give 16.6 mg (39 % yield) of peptide **5a** as a white powder.

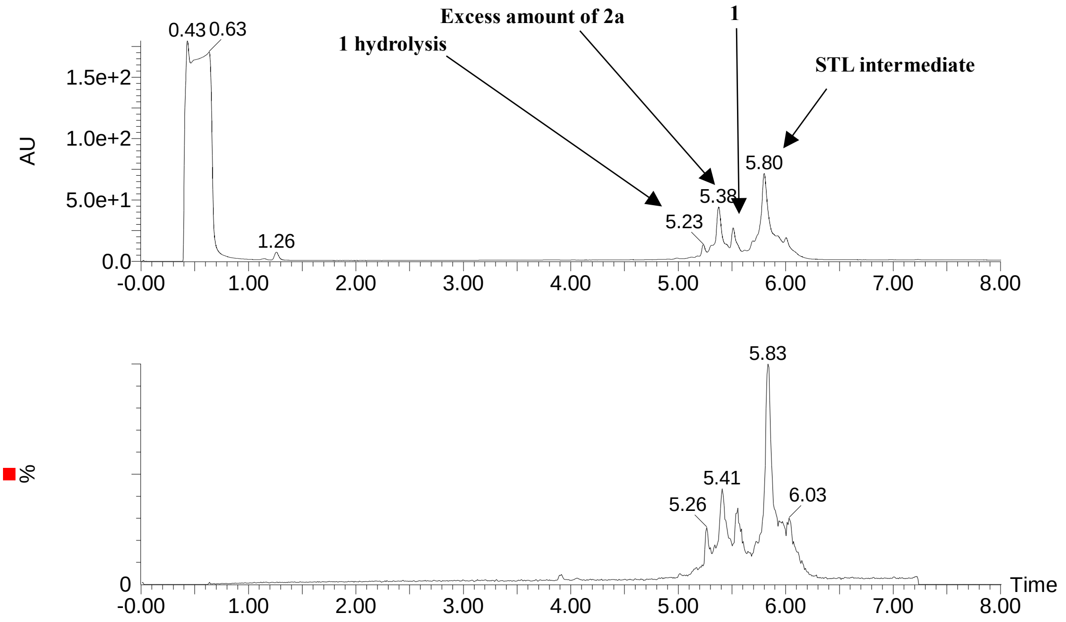

Figure 5.3-2 UV (190-400 nm) and MS (250-3000 m/z) trace from UPLC-MS analysis of **STL** between **2a** and **1** gradient 15-60% CH_3CN/H_2O containing 0.1% TFA over 8 min at a flow rate of 0.4 mL/min.

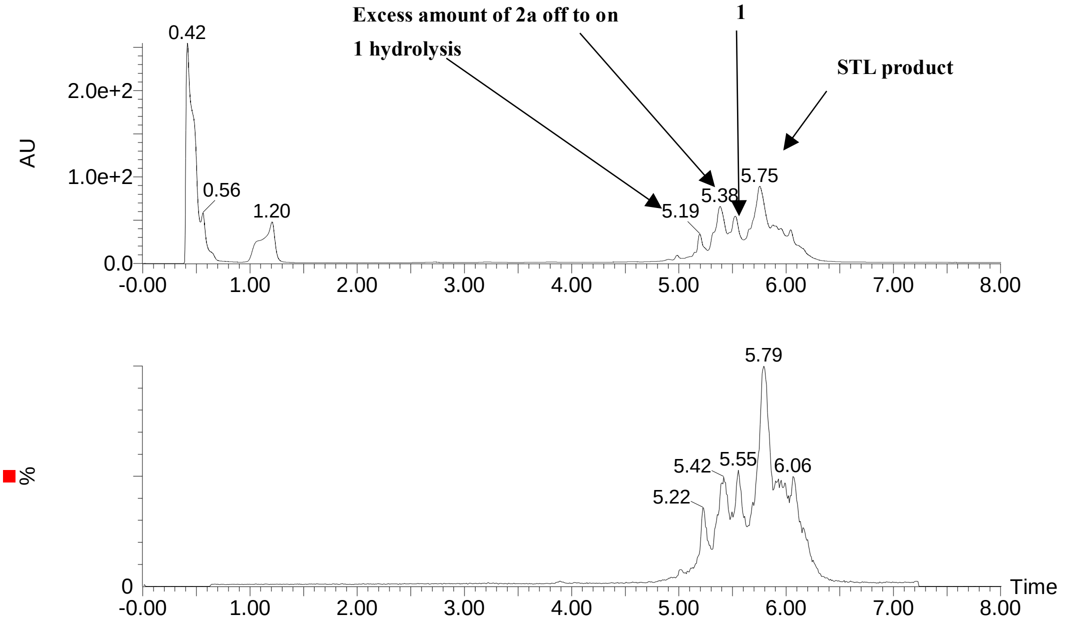

Figure 5.3-3 UV (190-400 nm) and MS (250-3000 m/z) trace from UPLC-MS analysis of **STL Acidolysis off-to-on** between **2a** and **1** gradient 15-60% CH_3CN/H_2O containing 0.1% TFA over 8 min at a flow rate of 0.4 mL/min.

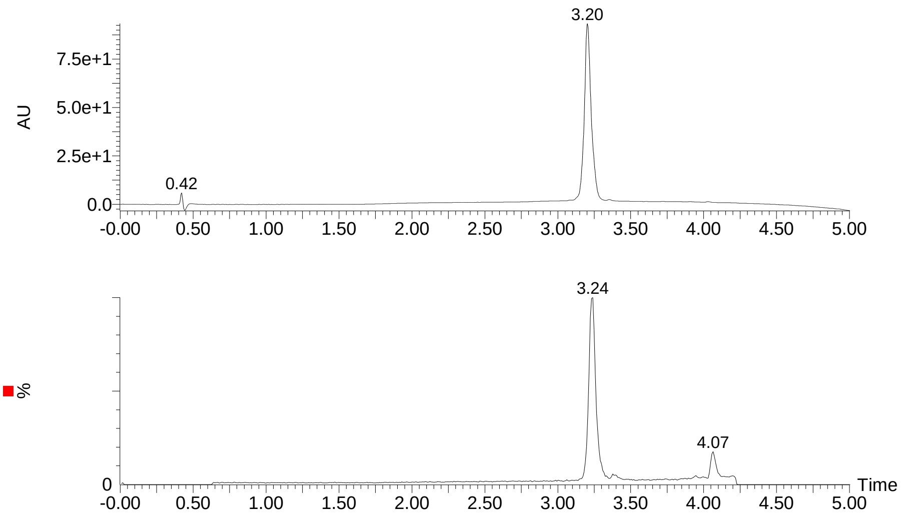

Figure 5.3-4 UV (190-400 nm) and MS (250-3000 m/z) trace from UPLC-MS analysis of **purified 5a** gradient 5-95% CH_3CN/H_2O containing 0.1% TFA over 5 min at a flow rate of 0.4 mL/min.

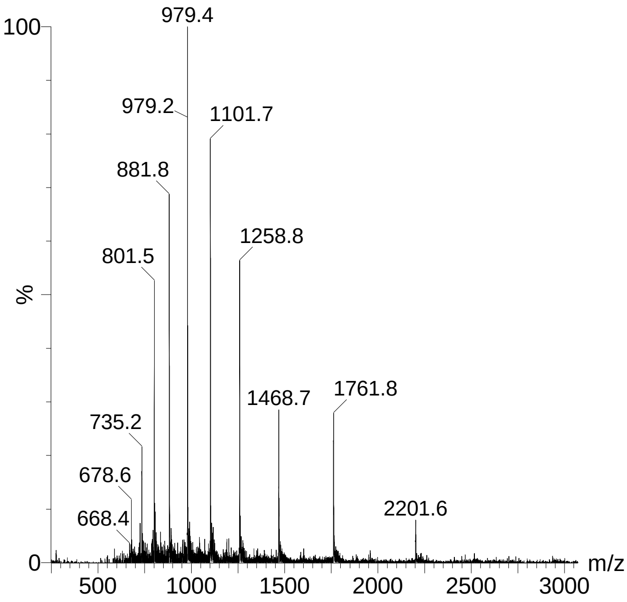

Figure 5.3-5 ESI-MS calcd. for $C_{398}H_{650}N_{100}O_{113}S_5$ $[M+4H]^{4+}$ m/z = 2202.1, found 2201.6; $[M+5H]^{5+}$ m/z = 1761.9, found 1761.8; $[M+6H]^{6+}$ m/z = 1468.4, found 1468.7; $[M+7H]^{7+}$ m/z = 1258.8, found 1258.8; $[M+8H]^{8+}$ m/z = 1101.6, found 1101.7; $[M+9H]^{9+}$ m/z = 979.3, found 979.4; $[M+10H]^{10+}$ m/z = 881.4, found 881.8; $[M+11H]^{11+}$ m/z = 801.4, found 801.5; $[M+12H]^{12+}$ m/z = 734.7, found 735.2; $[M+13H]^{13+}$ m/z = 678.3, found 678.6.

5.4 Synthesis of peptide fragment 6a

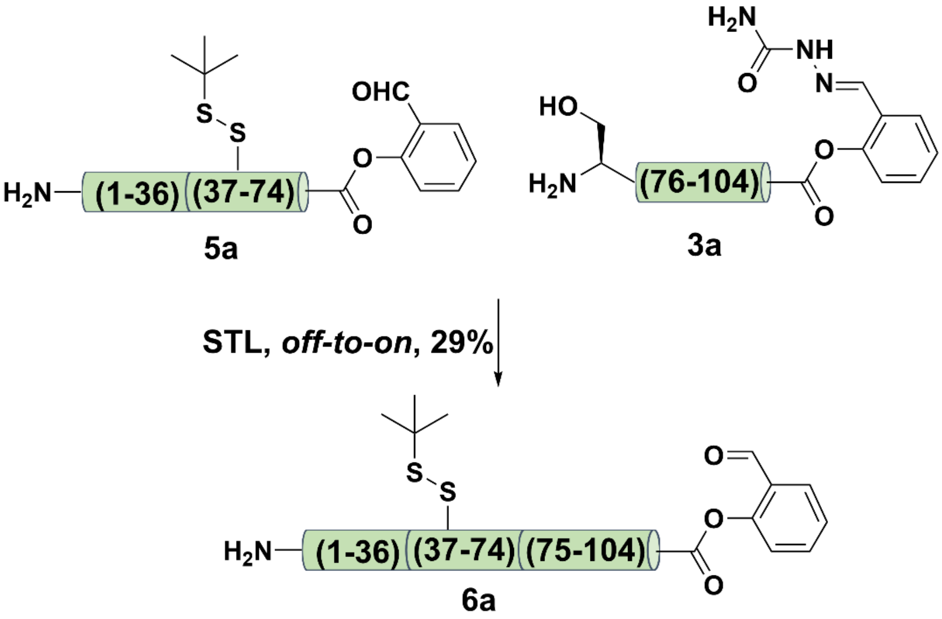

Figure 5.4-1 Synthetic scheme of **6a**

5a (10.1 mg, 1.1 µmol) and **5a** (4.8 mg, 1.3 µmol) were dissolved in pyridine/acetic acid (1/1, mol/mol) buffer at a concentration of 10 mM under room temperature. The reaction mixture was stirred at room temperature for 6 h. After completion of the reaction, the solvent was blown off under a stream of condensed N_2. The residue was treated with 3.0 mL of TFA/H_2O/Pyruvic acid (95/4/1, v/v/v) for 30 min. After that, the TFA was blown off under a stream of condensed N_2 and treated with cold diethyl ether to give a white suspension for centrifugation.

After decanting diethyl ether, the remaining solid was dissolved in 50% ACN/H_2O, then it was filtrated by Syringe Filters (PTFE 0.22µm) and subjected to preparative HPLC purification (15-60% CH_3CN/H_2O over 30 min) and lyophilization to give 4.1 mg (29 % yield) of peptide **6a** as a white powder.

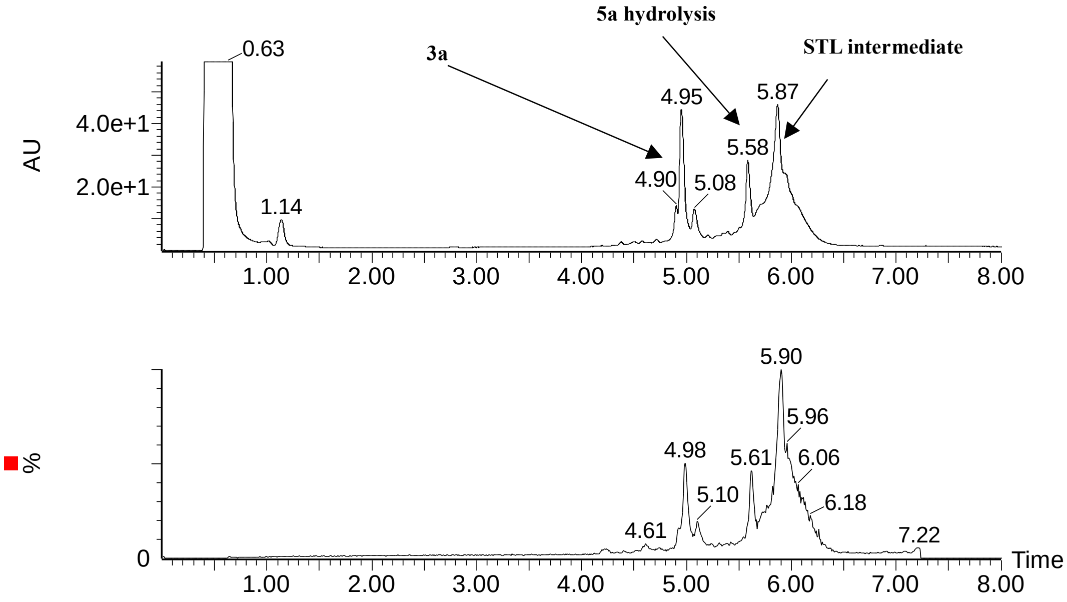

Figure 5.4-2 UV (190-400 nm) and MS (250-3000 m/z) trace from UPLC-MS analysis of **STL** between **3a** and **5a** gradient 15-60% CH_3CN/H_2O containing 0.1% TFA over 8 min at a flow rate of 0.4 mL/min.

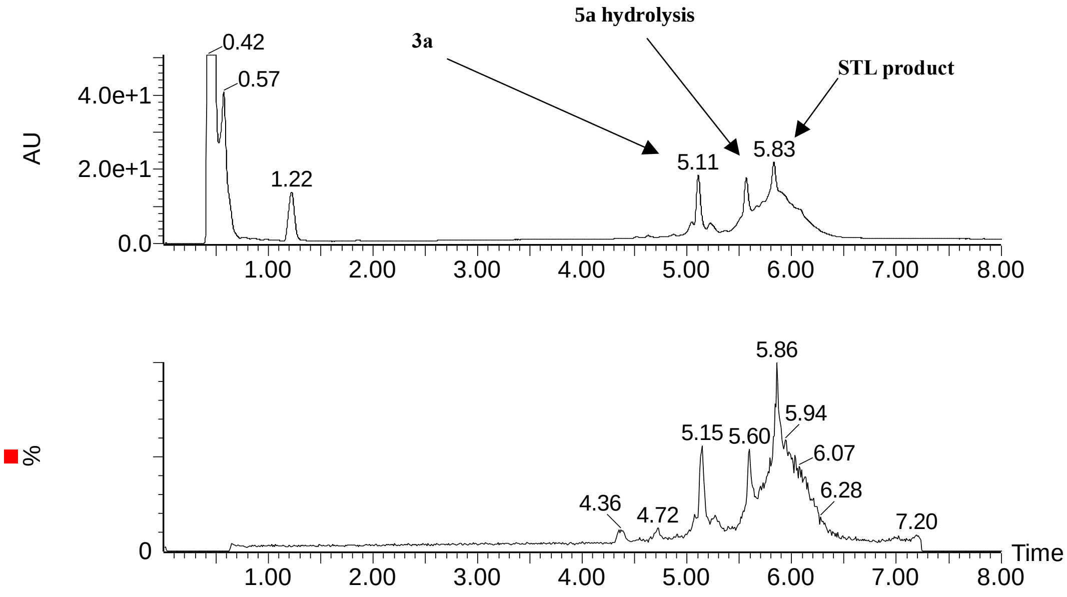

Figure 5.4-3 UV (190-400 nm) and MS (250-3000 m/z) trace from UPLC-MS analysis of **STL Acidolysis off-to-on** between **5a** and **3a** gradient 15-60% CH_3CN/H_2O containing 0.1% TFA over 8 min at a flow rate of 0.4 mL/min.

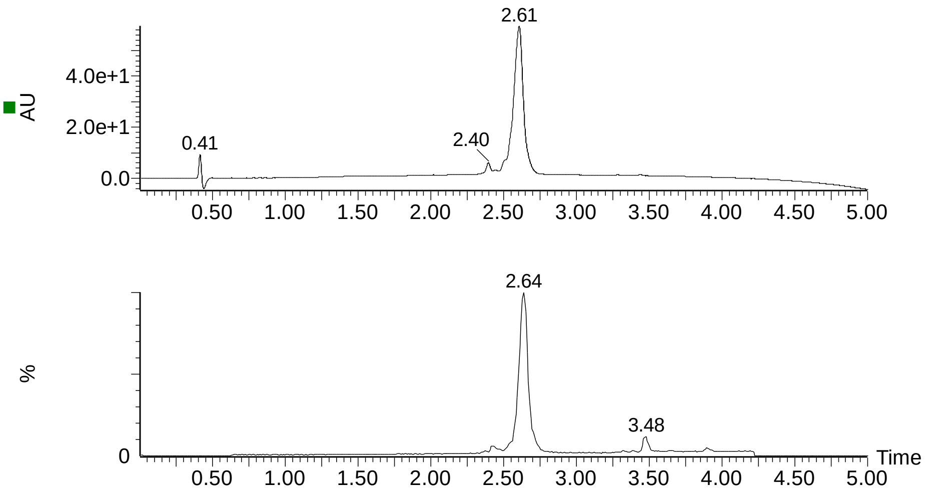

Figure 5.4-4 UV (190-400 nm) and MS (250-3000 m/z) trace from UPLC-MS analysis of **purified 6a** gradient 25-95% CH_3CN/H_2O containing 0.1% TFA over 5 min at a flow rate of 0.4 mL/min.

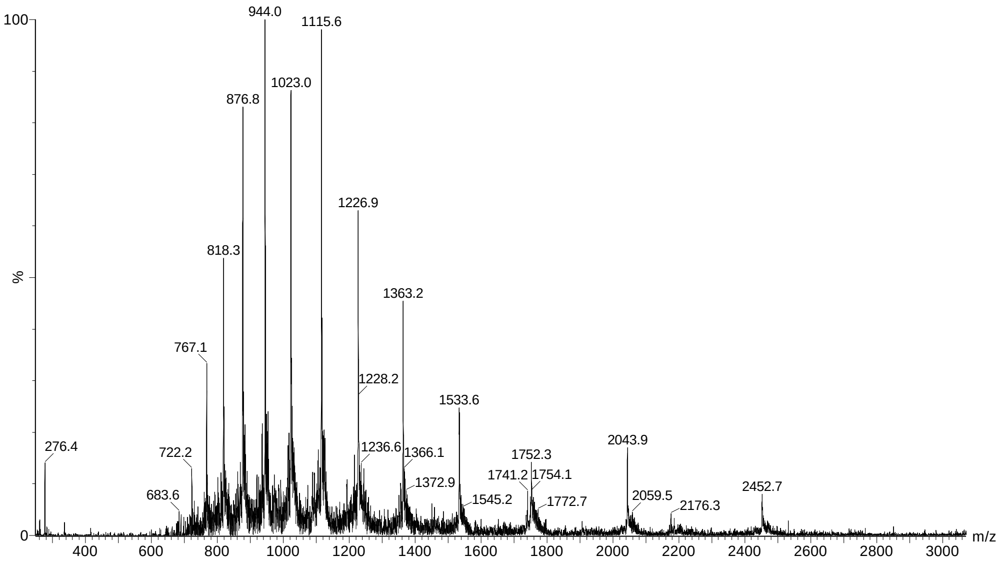

Figure 5.4-5 ESI-MS calcd. for $C_{553}H_{903}N_{143}O_{157}S_6$ $[M+5H]^{5+}$ m/z = 2452.9, found 2452.7; $[M+6H]^{6+}$ m/z = 2044.3, found 2043.9; $[M+7H]^{7+}$ m/z = 1752.7, found 1752.3; $[M+8H]^{8+}$ m/z = 1533.4, found 1533.6; $[M+9H]^{9+}$ m/z = 1363.2, found 1363.2; $[M+10H]^{10+}$ m/z = 1226.9, found 1226.9; $[M+11H]^{11+}$ m/z = 1115.5, found 1115.6; $[M+12H]^{12+}$ m/z = 1022.6, found 1023.0; $[M+13H]^{13+}$ m/z = 944.0, found 944.0; $[M+14H]^{14+}$ m/z = 876.7, found 876.8; $[M+15H]^{15+}$ m/z = 818.3, found 818.3; $[M+16H]^{13+}$ m/z = 767.2, found 767.1; $[M+17H]^{17+}$ m/z = 722.1, found 722.2.

6. The Third Synthetic Attempt of Interleukin-2 (IL-2)

6.1 Synthesis of 3b

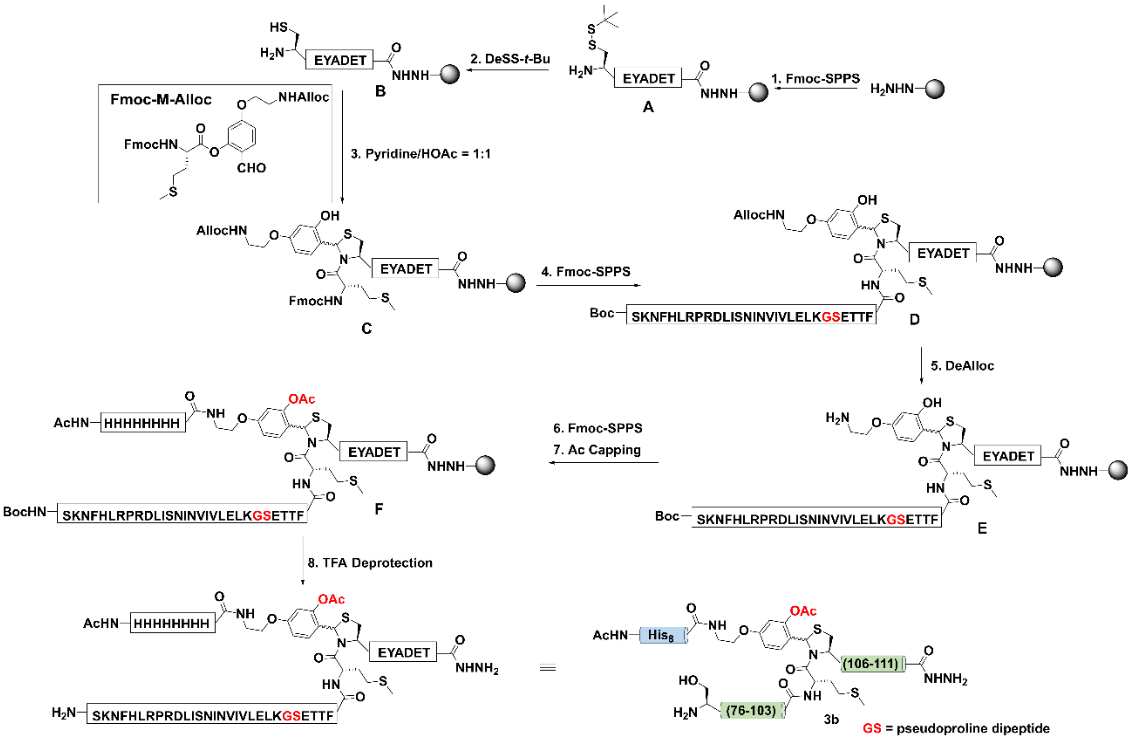

Figure 6.1-1 Synthetic scheme of **3b**

Resin bound peptide **A** was obtained by performing a general Fmoc-SPPS on 0.5 g hydrazine 2-chlorotrityl chloride (prepared by general procedure **2.5** using CS-bio 2-chlorotrityl chloride, loading: ~0.5 mmol/g). Then, the resin was swelled in a deS-*t*-bu reagent (10 mL DMF, 1 mL DODT, 0.2 mL DIPEA) for overnight to provide the desired intermediate **B**. Subsequently, the resin was washed by DMF and DCM, and 25 mL pyridine/HOAc buffer, containing SAL ester "**Fmoc-M-Alloc**" (15 mM), was added to the above resin. The resulting mixture was reacted at r.t. for 6 h, then the resin was washed by DMF and DCM. Another 25 mL pyridine/HOAc buffer, containing SAL ester "**Fmoc-M-Alloc**" (15 mM), was added to the resin and reacted for overnight.

After that, intermediate **C** was subjected to standard Fmoc-SPPS to afford desired intermediate **D**. Sequentially, a deAlloc procedure (10 mL DCM, 150 mg $Pd(PPh_3)_4$, 150 μL $PhSiH_3$, 2h) was applied to give the **E**, which underwent Fmoc-SPPS (using

Fmoc-His(Trt)-OH) and Ac capping (10 mL DCM, 0.2 mL Ac_2O, 0.4 mL TEA, 40 min) to generate the desired product **3b** in its protected form **F**.

Following a TFA deprotection (20 mL TFA/H_2O/TIPS = 95/2.5/2.5), cold ether precipitation, HPLC purification and freeze-drying, the desired product **3b** was obtained as a white powder (127.3 mg, 9%).

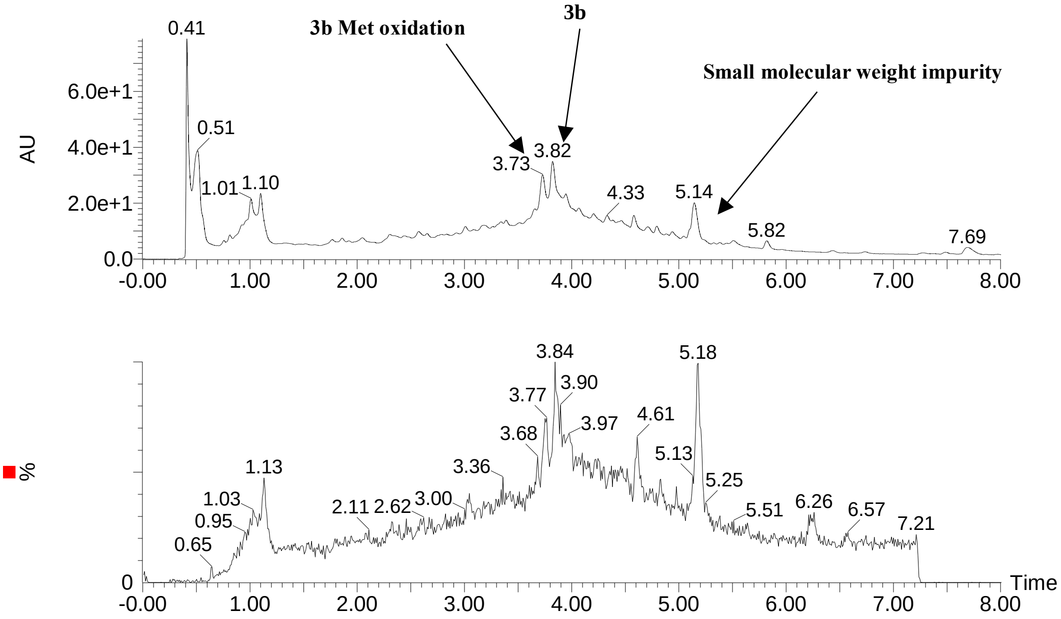

Figure 6.1-2 UV (190-400 nm) and MS (250-3000 m/z) trace from UPLC-MS analysis of **3b SPPS** gradient 15-60% CH_3CN/H_2O containing 0.1% TFA over 8 min at a flow rate of 0.4 mL/min.

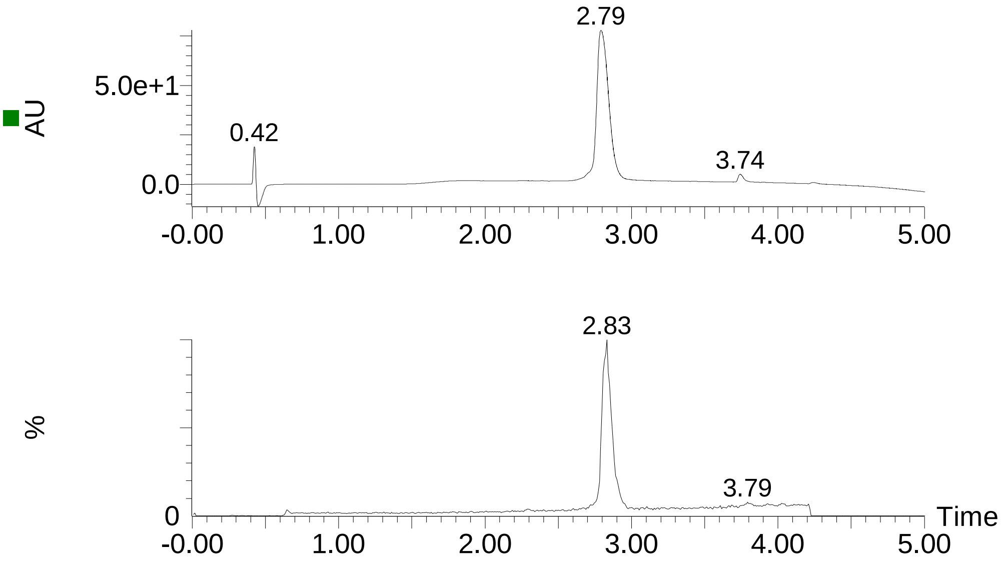

Figure 6.1-4 UV (190-400 nm) and MS (250-3000 m/z) trace from UPLC-MS analysis of **purified 3b** gradient 5-95% CH_3CN/H_2O containing 0.1% TFA over 5 min at a flow rate of 0.4 mL/min.

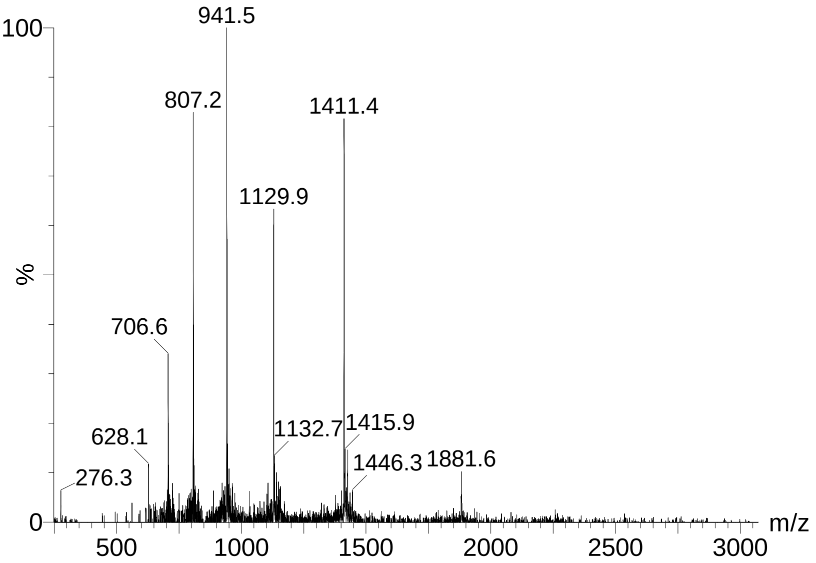

Figure 6.1-5 ESI-MS calcd. for $C_{249}H_{371}N_{77}O_{71}S_2$ $[M+3H]^{3+}$ m/z = 1882.1, found 1881.6; $[M+4H]^{4+}$ m/z = 1411.8, found 1411.4; $[M+5H]^{5+}$ m/z = 1129.7, found 1129.9; $[M+6H]^{6+}$ m/z = 941.6, found 941.5; $[M+7H]^{7+}$ m/z = 807.2, found 807.2; $[M+8H]^{8+}$ m/z = 706.4, found 706.6; $[M+9H]^{9+}$ m/z = 628.0, found 628.1.

6.2 Synthesis of 3c

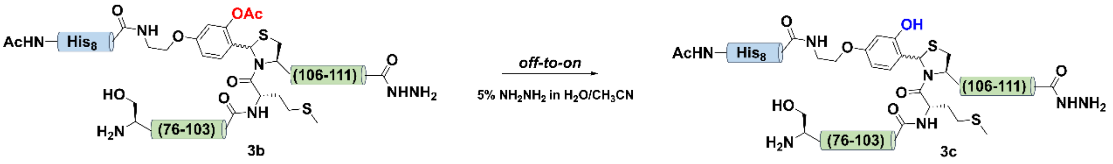

Figure 6.2-1 Synthetic of 3c

Peptide **3b** (37.2 mg) was dissolved in 4 mL 50% ACN/H_2O, then 0.2 mL hydrazine monohydrate was added. The solution was stirred at room temperature for 30 min to remove the Ac group. After that, the solution pH was adjusted to 3 by 10% TFA aqueous solution and subjected to HPLC purification and freeze-drying. The desired product **3c** was obtained as a white powder (24.5 mg, 66%).

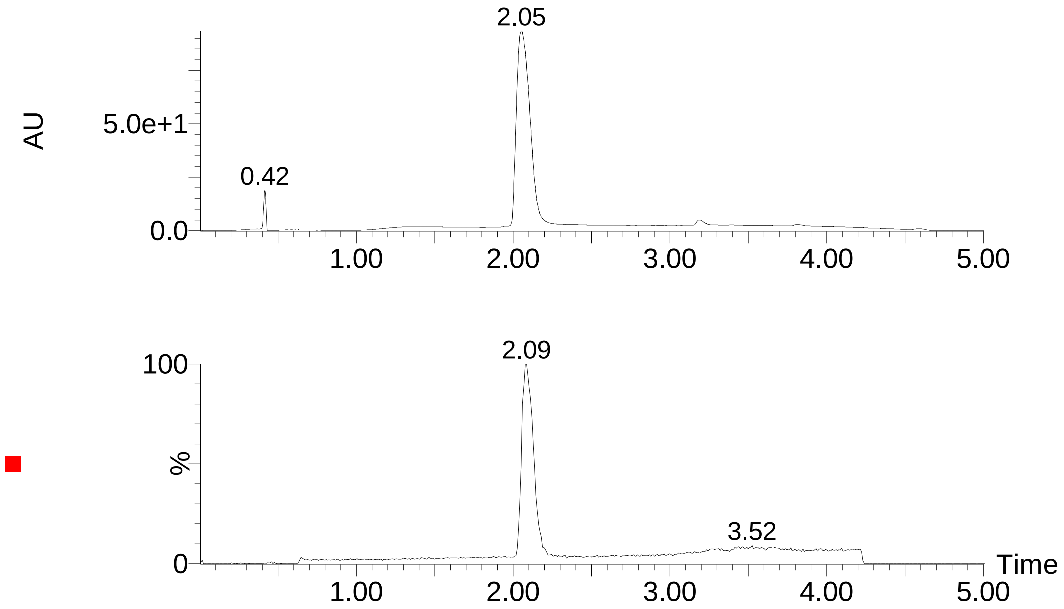

Figure 6.2-2 UV (190-400 nm) and MS (250-3000 m/z) trace from UPLC-MS analysis of **purified 3c** gradient 25-95% CH_3CN/H_2O containing 0.1% TFA over 5 min at a flow rate of 0.4 mL/min.

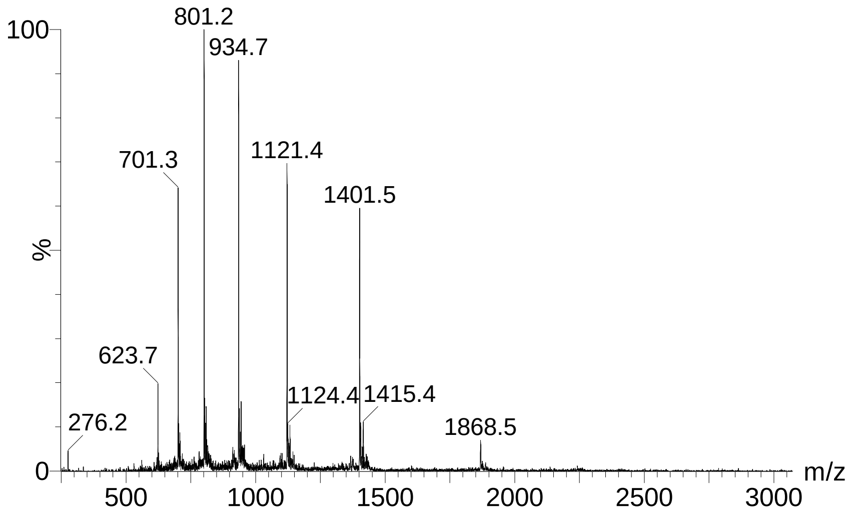

Figure 6.2-3 ESI-MS calcd. for $C_{247}H_{369}N_{77}O_{70}S_2$ $[M+3H]^{3+}$ m/z = 1868.1, found 1297.8; $[M+4H]^{4+}$ m/z = 1401.3, found 1401.5; $[M+5H]^{5+}$ m/z = 1121.3, found 1121.4; $[M+6H]^{6+}$ m/z = 934.5, found 934.7; $[M+7H]^{7+}$ m/z = 801.2, found 801.2; $[M+8H]^{8+}$ m/z = 701.2, found 701.3; $[M+9H]^{9+}$ m/z = 623.4, found 623.7.

6.3 Synthesis of 4n

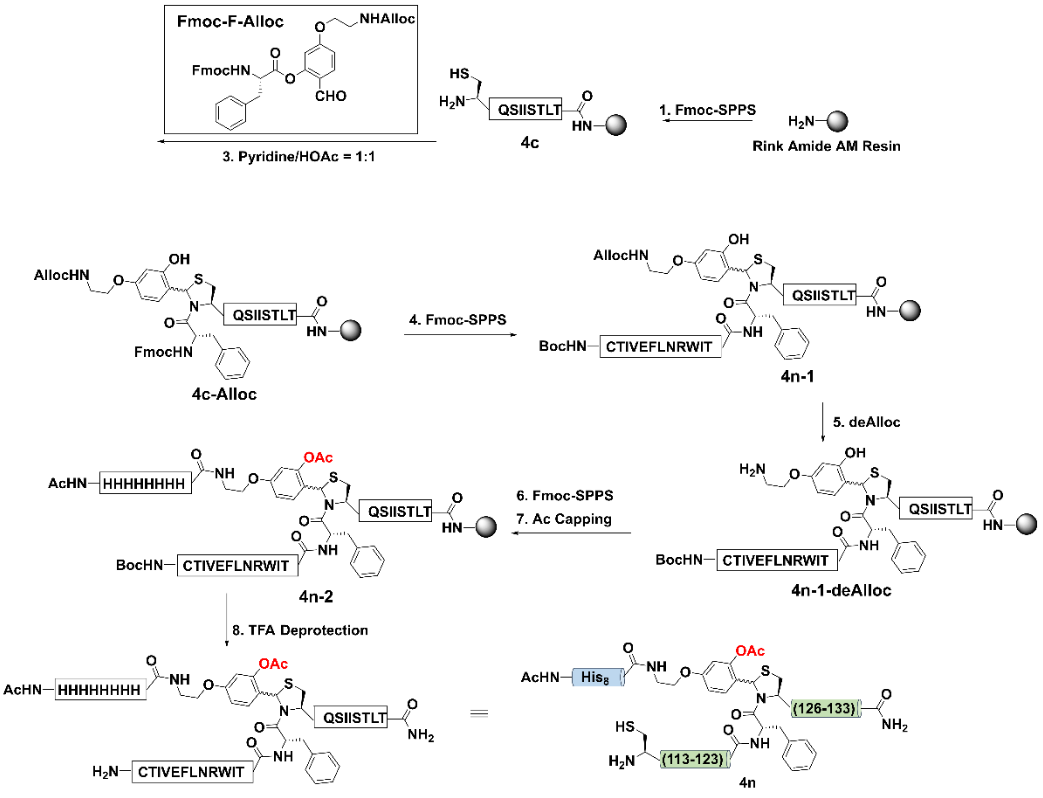

Figure 6.3-1 Synthetic scheme of **4n**

0.5 g Rink amide resin was employed in the synthesis of resin bound peptide **4c** by following procedure **3.1**. Subsequently, the resin was washed by DMF and DCM, and 25 mL pyridine/HOAc buffer, containing SAL ester "**Fmoc-F-Alloc**" (15 mM), was added to the above resin. The resulting mixture was reacted at r.t. for 6 h, then the resin was washed by DMF and DCM. Another 25 mL pyridine/HOAc buffer, containing SAL ester "**Fmoc-F-Alloc**" (15 mM), was added to the resin and reacted for overnight.

After that, intermediate **4c-Alloc** was subjected to standard Fmoc-SPPS to afford desired intermediate **4n-1**. Sequentially, a deAlloc procedure (10 mL DCM, 150 mg $Pd(PPh_3)_4$, 150 μL $PhSiH_3$, 2h) was applied to give the **4n-1-deAlloc**, which underwent Fmoc-SPPS (using Fmoc-His(Trt)-OH) and Ac capping (10 mL DCM, 0.2 mL Ac_2O, 0.4 mL TEA, 40 min) to generate the desired product **4n** in its protected form **4n-2**.

Following a TFA deprotection (20 mL TFA/H_2O/TIPS = 95/2.5/2.5), cold ether precipitation, HPLC purification and freeze-drying, the desired product **4n** was obtained as a white powder (108.2 mg, 11%).

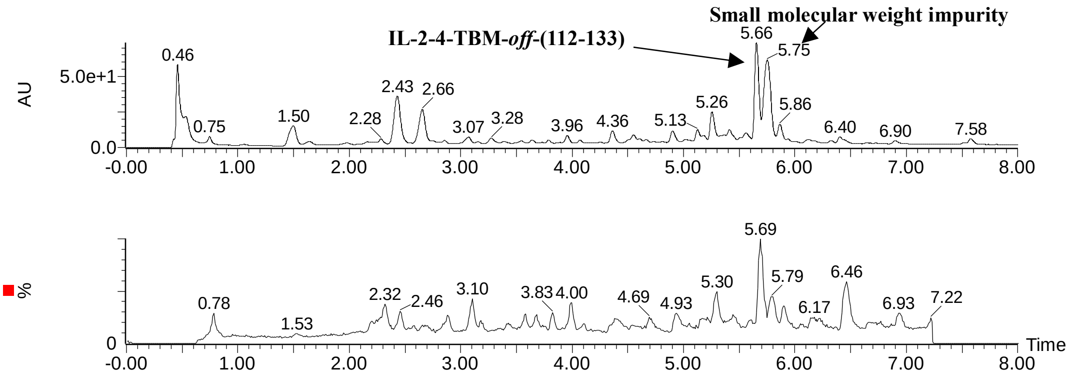

Figure 6.3-2 UV (190-400 nm) and MS (250-3000 m/z) trace from UPLC-MS analysis of **4n** gradient 15-60% CH_3CN/H_2O containing 0.1% TFA over 8 min at a flow rate of 0.4 mL/min.

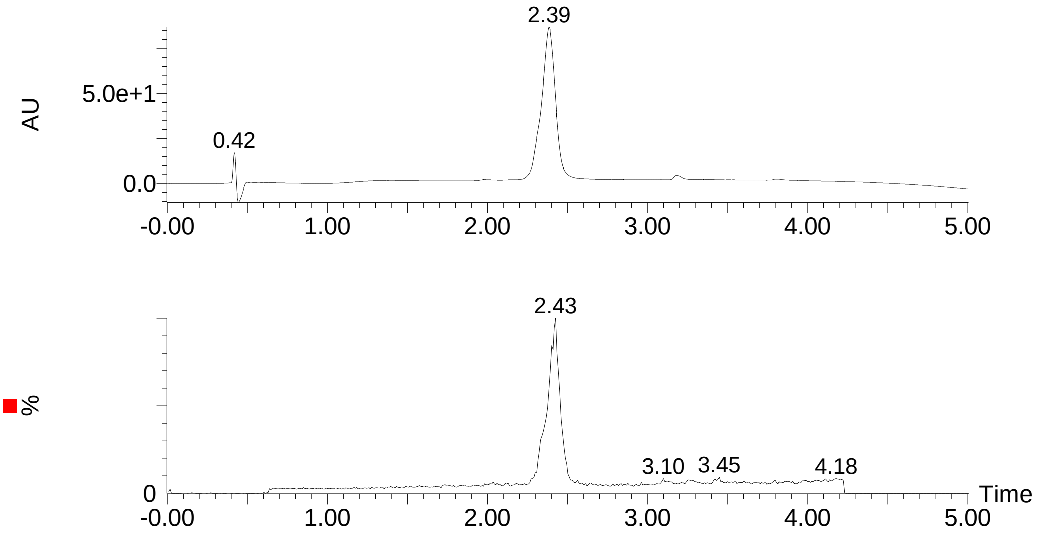

Figure 6.3-3 UV (190-400 nm) and MS (250-3000 m/z) trace from UPLC-MS analysis of **Purified 4n** gradient 5-95% CH_3CN/H_2O containing 0.1% TFA over 5 min at a flow rate of 0.4 mL/min.

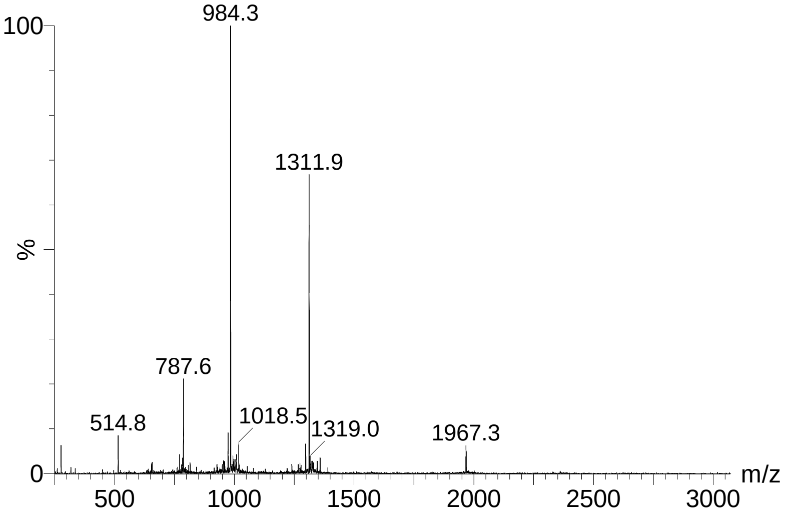

Figure 6.3-4 ESI-MS calcd. for $C_{179}H_{255}N_{53}O_{45}S_2$ $[M+2H]^{2+}$ m/z = 1967.7, found 1967.3; $[M+3H]^{3+}$ m/z = 1312.2, found 1311.9; $[M+4H]^{4+}$ m/z = 984.4, found 984.3; $[M+5H]^{5+}$ m/z = 787.7, found 787.6.

6.4 Synthesis of 4o

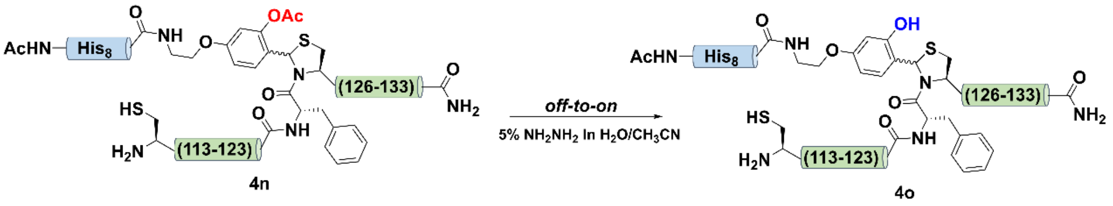

Figure 6.4-1 Synthetic Scheme of 4o

Peptide **4n** (50.3 mg) was dissolved in 5 mL 50% ACN/H_2O containing 5% hydrazine, then the solution was stirred at room temperature for 30 min. After that, the solution pH was adjusted to 3 by 10% TFA aqueous solution and subjected to HPLC purification and freeze-drying. The desired product **4o** was obtained as a white powder (26.6 mg, 53%).

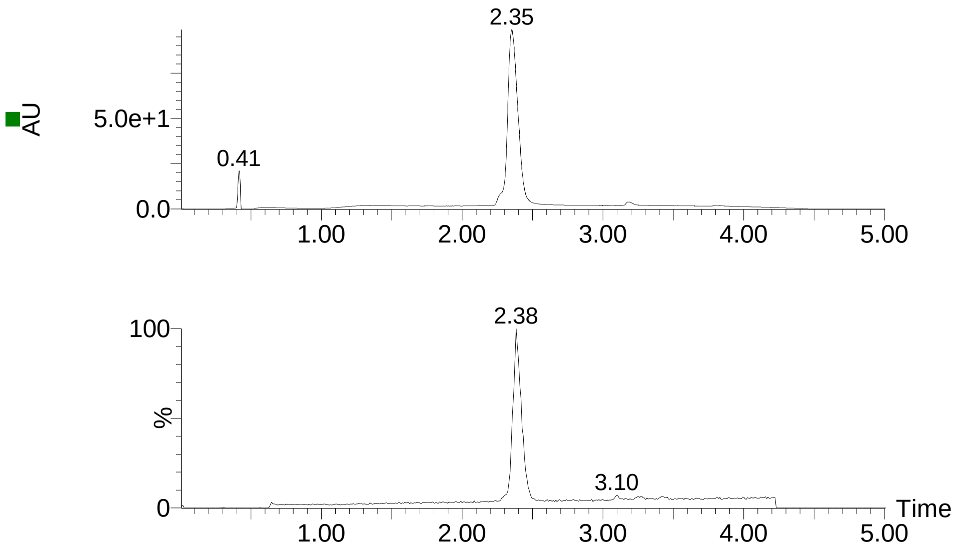

Figure 6.4-2 UV (190-400 nm) and MS (250-3000 m/z) trace from UPLC-MS analysis of **purified 4o** gradient 25-95% CH_3CN/H_2O containing 0.1% TFA over 5 min at a flow rate of 0.4 mL/min.

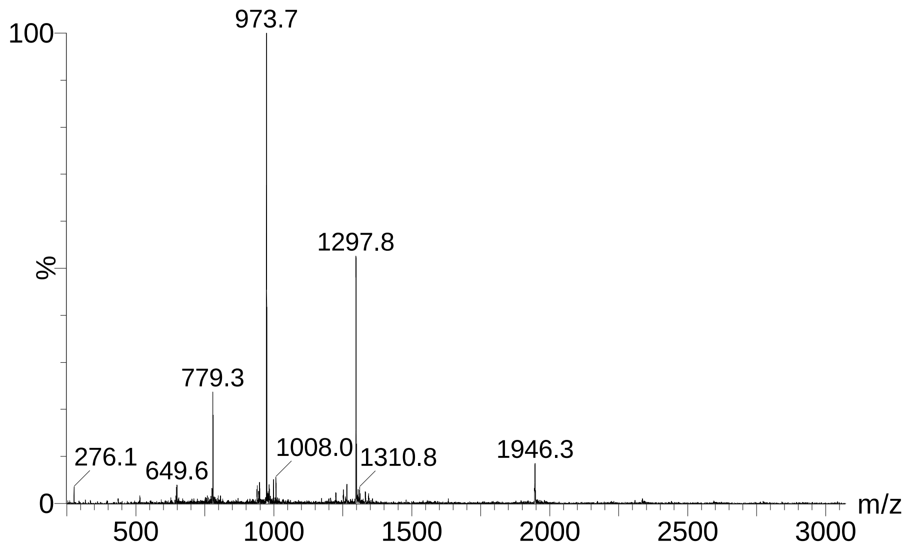

Figure 6.4-3 ESI-MS calcd. for $C_{177}H_{253}N_{53}O_{44}S_2$ $[M+2H]^{2+}$ m/z = 1946.7, found 1946.3; $[M+3H]^{3+}$ m/z = 1298.1, found 1297.8; $[M+4H]^{4+}$ m/z = 973.8, found 973.7; $[M+5H]^{5+}$ m/z = 779.3, found 779.3; $[M+6H]^{6+}$ m/z = 649.6, found 649.6.

6.5 Synthesis of 8

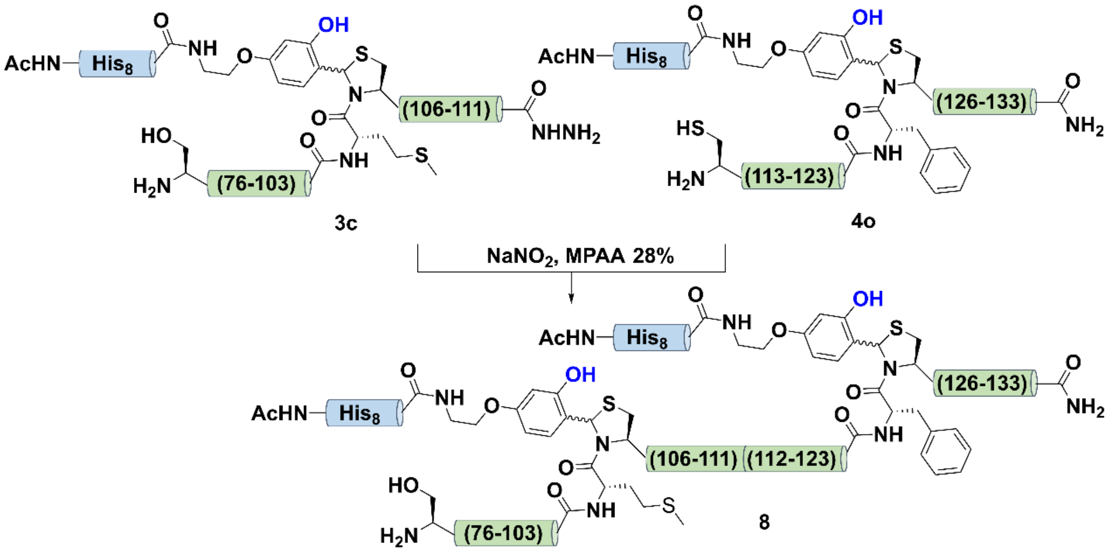

Figure 6.5-1 Synthetic Scheme of 8

Peptide **3c** (24.5 mg, 4.37 μmol) was dissolved in 0.5 mL aqueous buffer containing 6 M GnHCl and 0.2 M NaH_2PO_4 (pH = 3.0), and cooled to approximately -15°C in an

ice-salt bath. 18 μL of 0.5 M $NaNO_2$ was then added to activate the peptide hydrazide and stirred at -15°C for 15 min. After that, 4-mercaptophenylacetic acid (MPAA, 24.6 mg, 146 μmol) was dissolved into 0.5 mL 0.2 M NaH_2PO_4 solution containing 6 M GnHCl (pH 7.0), then this solution was added into the above mixture. The reaction was stirred for 20 min, then taken out of the ice-salt bath. Subsequently, peptide **4o** (20.4 mg, 5.2 μmol) was added into the reaction mixture, and the pH of reaction mixture was then adjusted to 6.5 slowly with aqueous NaOH solution (6 M and 1M) to initiate the NCL at room temperature (peptide final concentration is 3 mM). The reaction process was monitored by analytic RP-HPLC. After 24 h, no improvement of ligation yield, then the reaction mixture was subjected to HPLC purification (15-65% CH_3CN/H_2O over 30 min) and lyophilization to afford 11.6 mg (28 % yield) of peptide **8** as a white powder.

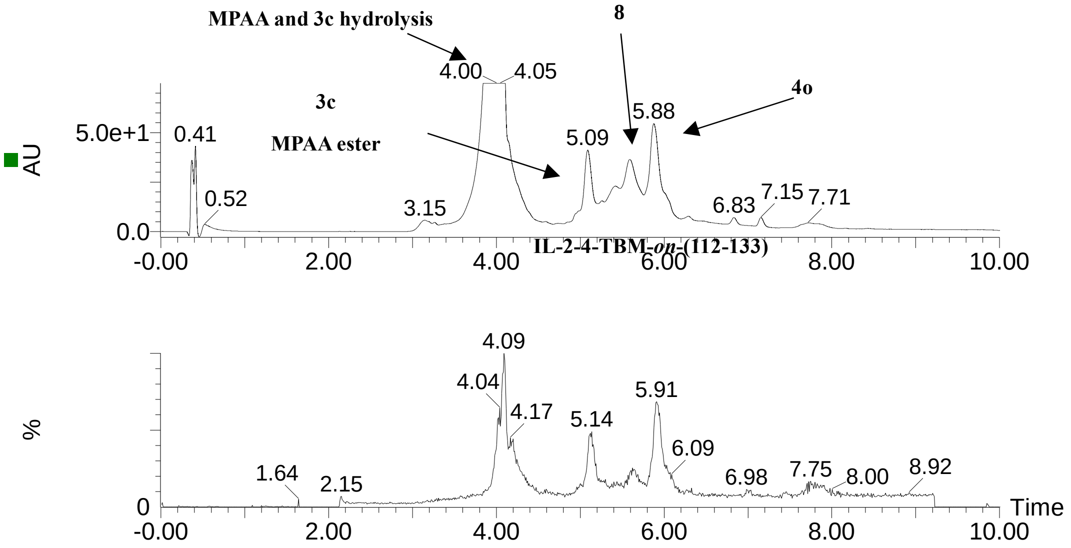

Figure 6.5-2 UV (190-400 nm) and MS (250-3000 m/z) trace from UPLC-MS analysis of NCL between **3c** and **4o** gradient 20-60% CH_3CN/H_2O containing 0.1% TFA over 10 min at a flow rate of 0.4 mL/min.

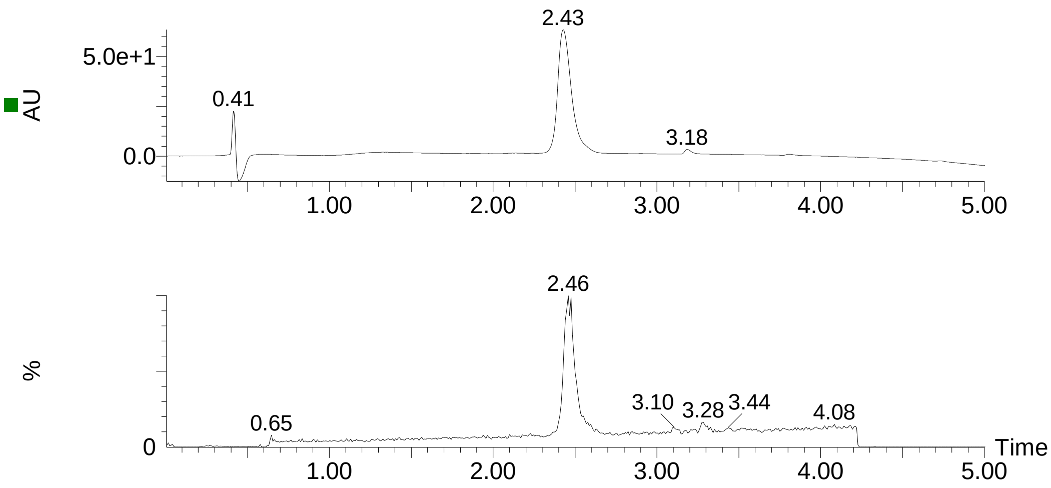

Figure 6.5-3 UV (190-400 nm) and MS (250-3000 m/z) trace from UPLC-MS analysis of purified **8** gradient 5-95% CH_3CN/H_2O containing 0.1% TFA over 5 min at a flow rate of 0.4 mL/min.

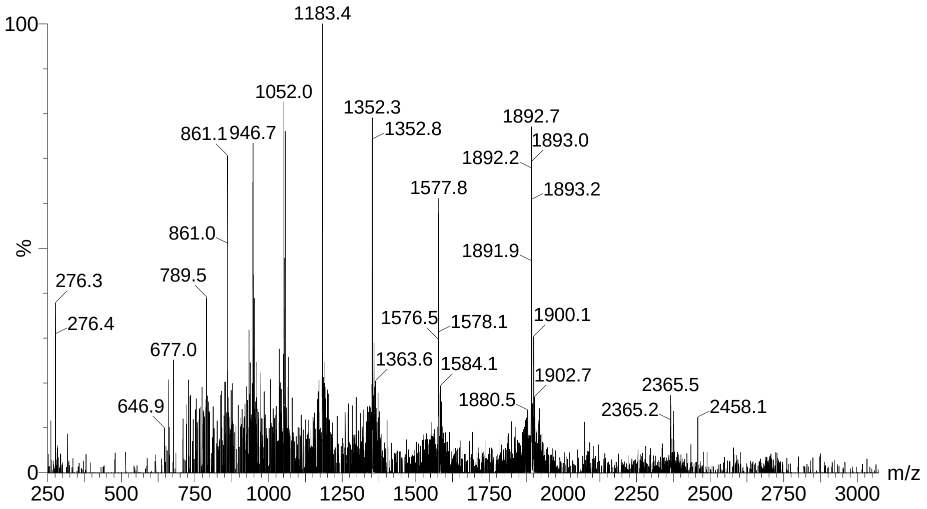

Figure 6.5-4 ESI-MS calcd. for $C_{424}H_{618}N_{128}O_{114}S_4$ $[M+4H]^{4+}$ m/z = 2366.1, found 2365.5; $[M+5H]^{5+}$ m/z = 1893.1, found 1893.0; $[M+6H]^{6+}$ m/z = 1577.8, found 1577.8; $[M+7H]^{7+}$ m/z = 1352.5, found 1352.3; $[M+8H]^{8+}$ m/z = 1183.6, found 1183.4; $[M+9H]^{9+}$ m/z = 1052.2, found 1052.0; $[M+10H]^{10+}$ m/z = 947.0, found 946.7; $[M+11H]^{11+}$ m/z = 861.1, found 861.1; $[M+12H]^{12+}$ m/z = 789.4, found 789.5.

6.6 Synthesis of 8a

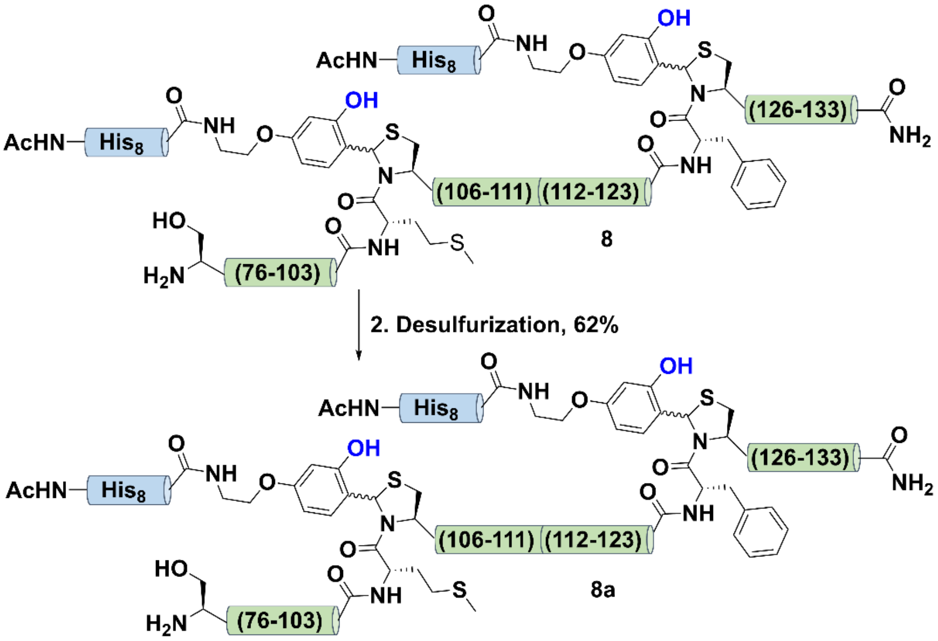

Figure 6.6-1 Synthetic Scheme of **8a**

8 (11.6 mg) was dissolved by 1.5 mL 0.2 M NaH_2PO_4 solution containing 6 M GnHCl and 0.5 M TCEP. After that, the solution pH was adjusted to 6.5 by 6M NaOH (aq) solution. Subsequently, VA044 aqueous solution (64mg/mL) 150 µL and 30 µL *t*-Bu-SH were added into the above peptide solution. This reaction mixture was stirred at 37 °C for 18 h, and UPLC-MS indicated that the reaction was completed. This solution was diluted by water and filtrated by Syringe Filters (PTFE 0.22µm), then it was subjected to preparative HPLC purification (20-60% CH_3CN/H_2O over 35 min, C4 column) and lyophilization to give **8a** (7.2 mg, 62 %) as a white powder.

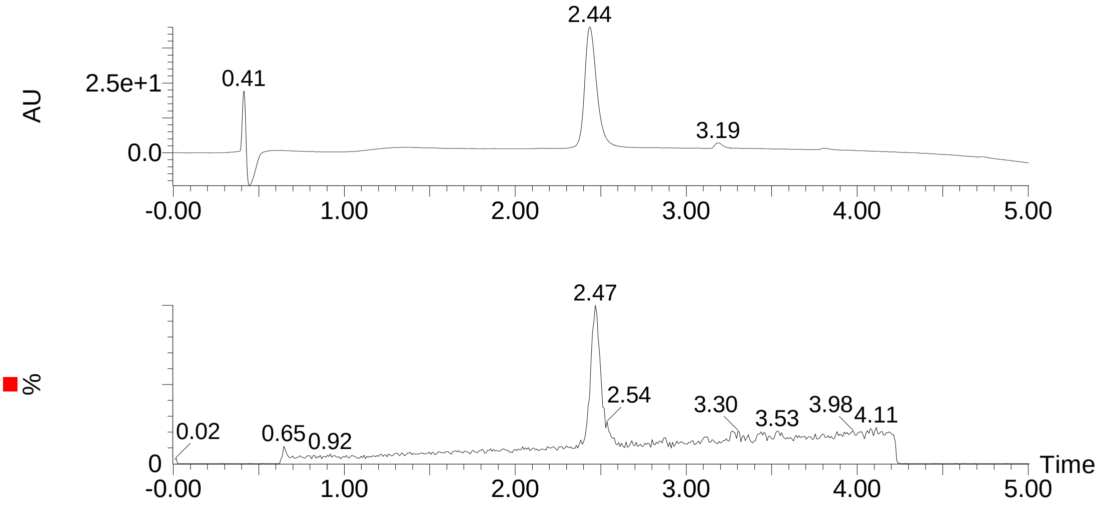

Figure 6.6-2 UV (190-400 nm) and MS (250-3000 m/z) trace from UPLC-MS analysis of purified **8a** gradient 5-95% CH_3CN/H_2O containing 0.1% TFA over 5 min at a flow rate of 0.4 mL/min.

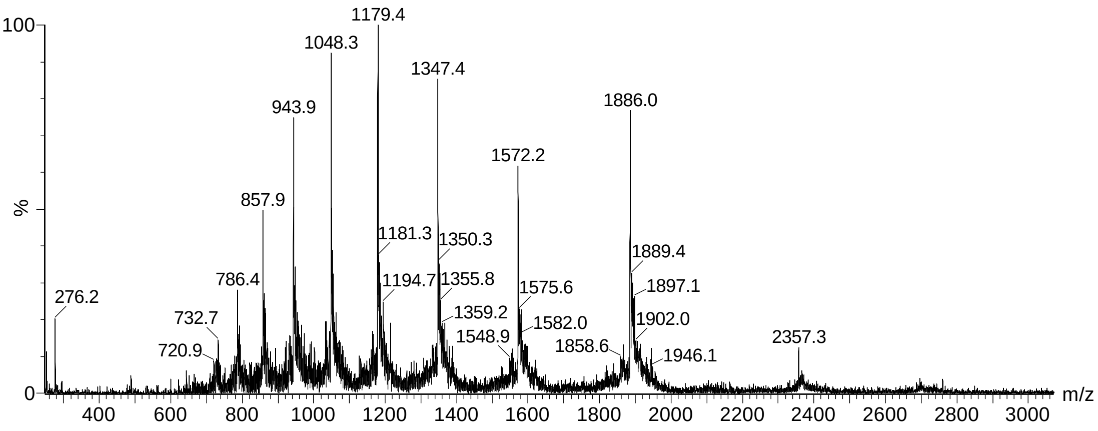

Figure 6.6-3 ESI-MS calcd. for $C_{424}H_{619}N_{129}O_{113}S_3$ $[M+4H]^{4+}$ m/z = 2357.9, found 2357.3; $[M+5H]^{5+}$ m/z = 1886.5, found 1886.0; $[M+6H]^{6+}$ m/z = 1572.3, found 1572.2; $[M+7H]^{7+}$ m/z = 1347.8, found 1347.4; $[M+8H]^{8+}$ m/z = 1179.5, found 1179.4; $[M+9H]^{9+}$ m/z = 1048.5, found 1048.3; $[M+10H]^{10+}$ m/z = 943.8, found 943.9; $[M+11H]^{11+}$ m/z = 858.1, found 857.9; $[M+12H]^{12+}$ m/z = 786.6, found 786.4.

6.7 Synthesis of peptide fragment 7b

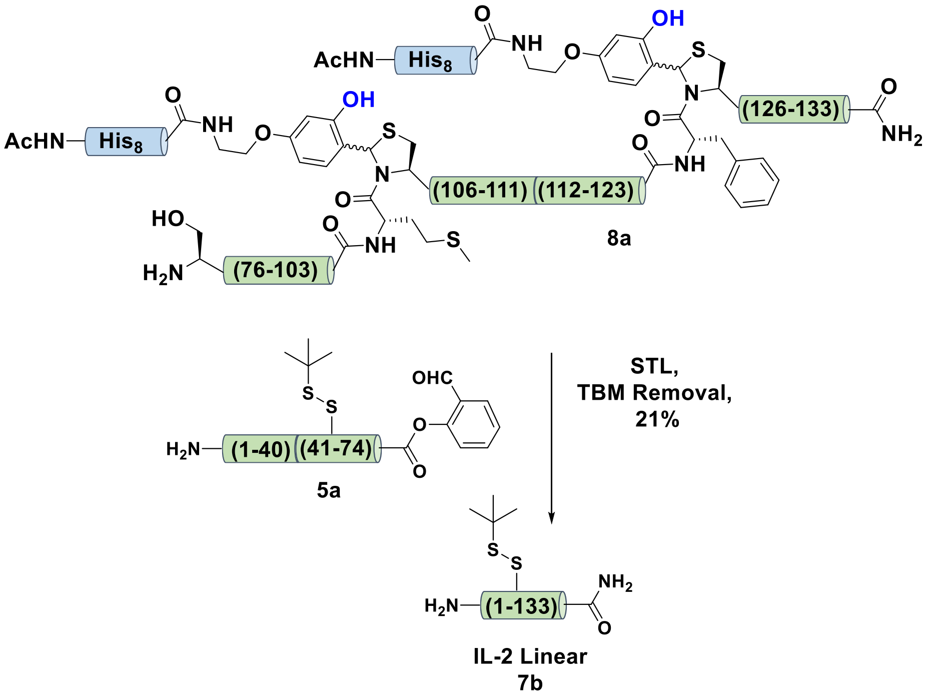

Figure 6.7-1 Synthetic Scheme of **7b**

5a (15.2 mg, 1.7 µmol) and **8a** (16.0 mg, 1.7 µmol) were dissolved in collidine/acetic acid (1/1, v/v) buffer at a concentration of 15 mM under room temperature. The reaction mixture was stirred at room temperature for 6 h. After completion of the reaction, the solvent was blown off under a stream of condensed N_2. The residue was treated with 3.0 mL of TFA/H_2O/ETD/TIPS (92.5/2.5/2.5/2.5, v/v/v) for 1.5 h. After that, the TFA was blown off under a stream of condensed N_2 and the residue was dissolved in 50% ACN/H_2O, then it was subjected to preparative HPLC purification (30-80% CH_3CN/H_2O over 35 min) and lyophilization to give 5.7 mg (21 % yield) of peptide **7b** as a white powder.

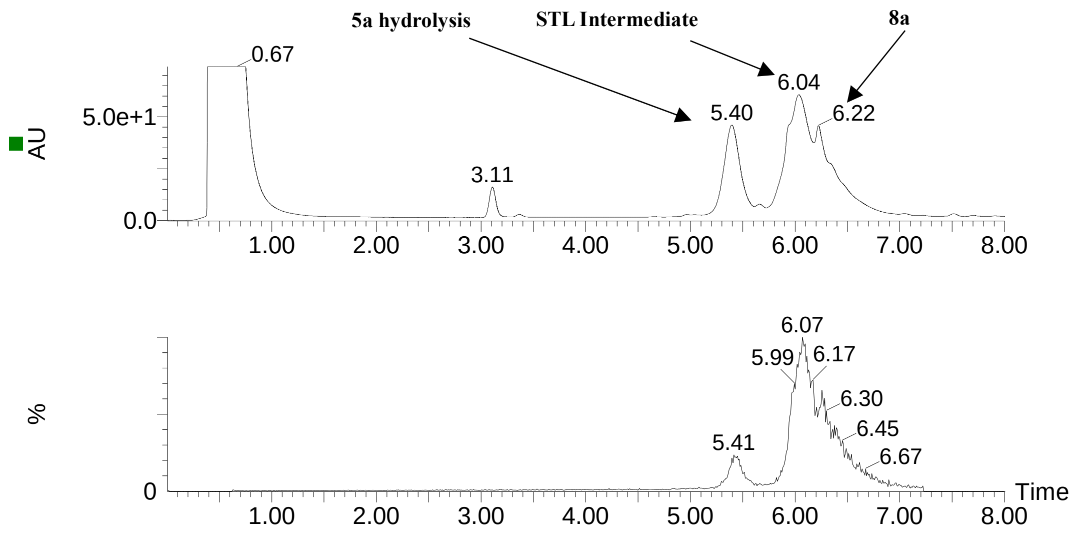

Figure 6.7-2 UV (190-400 nm) and MS (250-3000 m/z) trace from UPLC-MS analysis of **STL** between **5a** and **8a** gradient 20-60% CH_3CN/H_2O containing 0.1% TFA over 8 min at a flow rate of 0.4 mL/min.

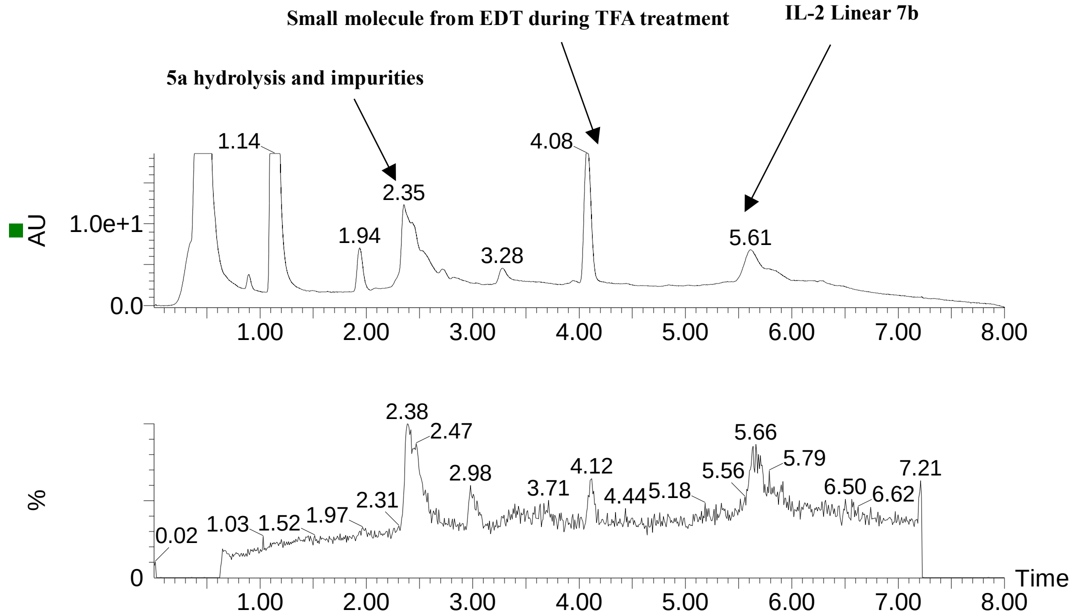

Figure 6.7-3 UV (190-400 nm) and MS (250-3000 m/z) trace from UPLC-MS analysis of **STL Acidolysis and TBM removal** gradient 30-80% CH_3CN/H_2O containing 0.1% TFA over 8 min at a flow rate of 0.4 mL/min

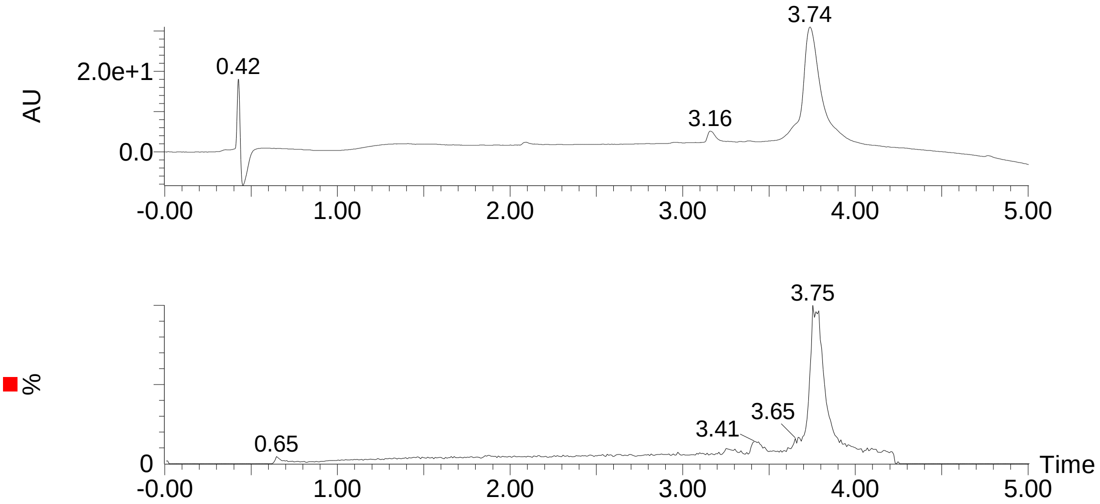

Figure 6.7-4 UV (190-400 nm) and MS (250-3000 m/z) trace from UPLC-MS analysis of **purified IL2 Linear 7b** gradient 25-95% CH_3CN/H_2O containing 0.1% TFA over 5 min at a flow rate of 0.4 mL/min.

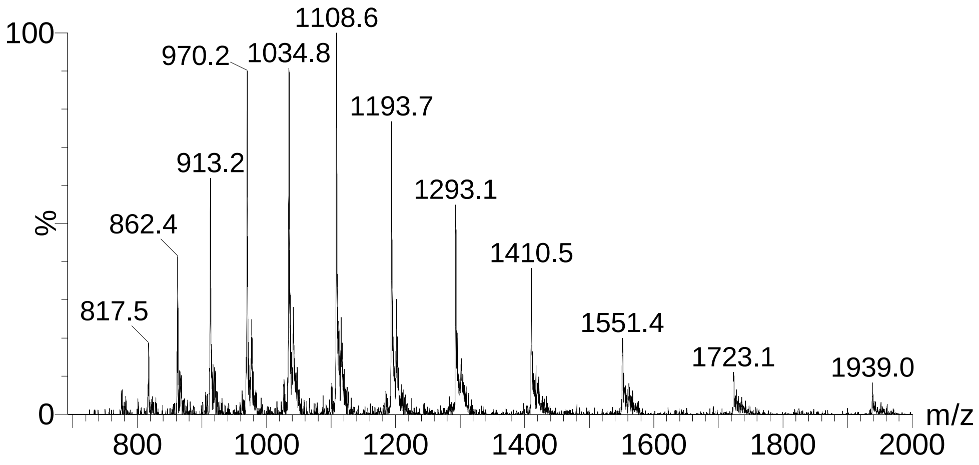

Figure 6.7-5 ESI-MS calcd. for $C_{697}H_{1129}N_{179}O_{202}S_8$ $[M+8H]^{8+}$ m/z = 1939.2, found 1939.0; $[M+9H]^{9+}$ m/z = 1723.8, found 1723.1; $[M+10H]^{10+}$ m/z = 1551.5, found 1551.4; $[M+11H]^{11+}$ m/z = 1410.6, found 1410.5; $[M+12H]^{12+}$ m/z = 1293.1, found 1293.1; $[M+13H]^{13+}$ m/z = 1193.7, found 1193.7; $[M+14H]^{14+}$ m/z = 1108.5, found 1108.6; $[M+15H]^{15+}$ m/z = 1034.7, found 1034.8; $[M+16H]^{16+}$ m/z = 970.1, found 970.2; $[M+17H]^{17+}$ m/z = 913.1, found 913.2; $[M+18H]^{18+}$ m/z = 862.4, found 862.4; $[M+19H]^{19+}$ m/z = 817.1, found 817.5.

6.8 Synthesis of folded IL-2

IL-2 Linear (5.7 mg) was dissolved in 6 M guanidine hydrochloride aqueous (28.5 mL) containing 0.1 M Tris and 30 mM reduced glutathione, which was adjusted to pH 8.0 by 6 M aq. HCl. The mixture was stored for 1 h at 50 ºC. Then the solution was pour into 57 mL refolding buffer (0.1 M Tri, 1.5 mM oxidized glutathione, pH = 8.0) and stored for 24 h. The resulting solution was concentrated by ultrafiltration, then it was subjected to C4 HPLC purification (30-80% ACN/H_2O, 35 min) and freeze-drying to give the desired folded IL-2 (0.6 mg, 10%)

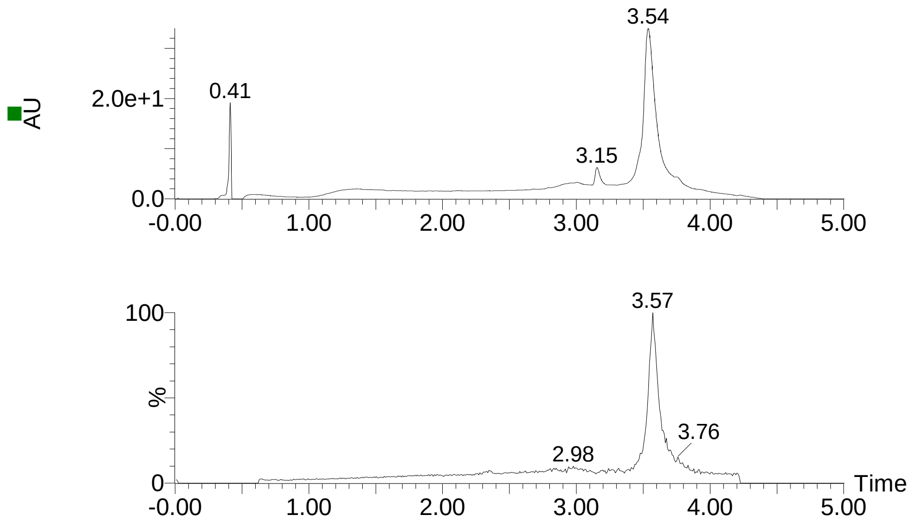

Figure 6.8-1 UV (190-400 nm) and MS (250-3000 m/z) trace from UPLC-MS analysis of **purified folded IL2** gradient 25-95% CH_3CN/H_2O containing 0.1% TFA over 5 min at a flow rate of 0.4 mL/min.

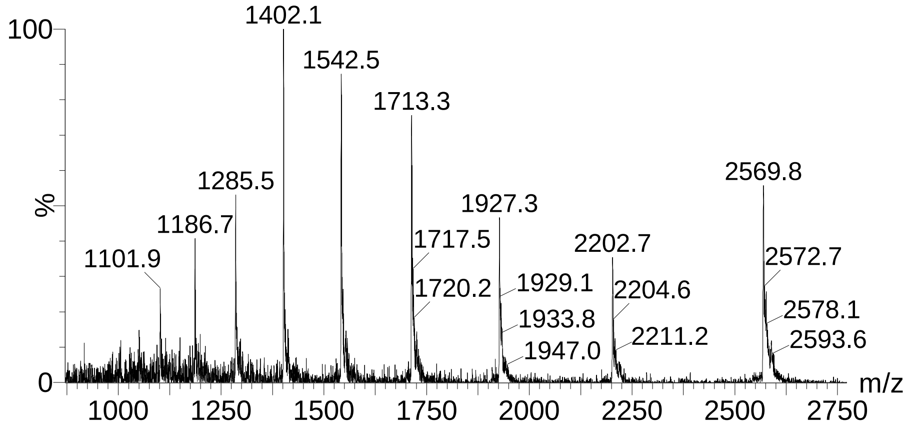

Figure 6.8-2 ESI-MS calcd. for $C_{693}H_{1119}N_{179}O_{202}S_7$ $[M+6H]^{6+}$ m/z = 2570.0, found 2569.8; $[M+7H]^{7+}$ m/z = 2203.0, found 2202.7; $[M+8H]^{8+}$ m/z = 1927.8, found 1927.3; $[M+9H]^{9+}$ m/z = 1713.7, found 1713.3; $[M+10H]^{10+}$ m/z = 1542.4, found 1542.5; $[M+11H]^{11+}$ m/z = 1402.3, found 1402.1; $[M+12H]^{12+}$ m/z = 1285.5, found 1285.5; $[M+13H]^{13+}$ m/z = 1186.7, found 1186.7; $[M+14H]^{14+}$ m/z = 1102.0, found 1101.9.

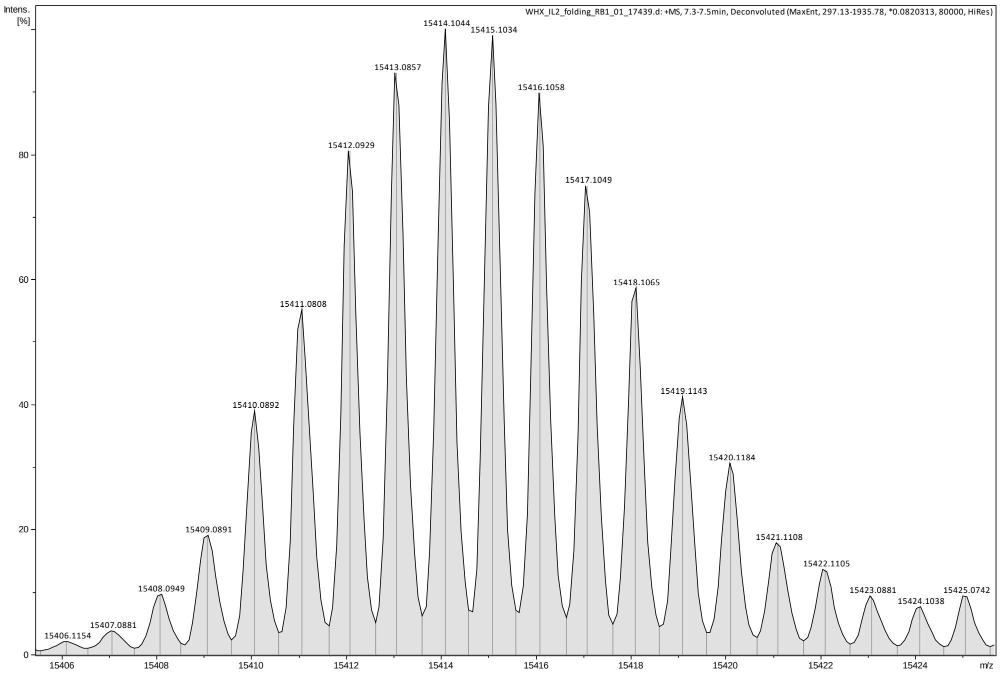

Figure 6.8-3 HRMS of **Folded IL-2 (1-133).** calcd. for $C_{693}H_{1119}N_{179}O_{202}S_7$ m/z = 15414.0566, measured 15414.1044. HRMS indicated the formation of disulfide bond.

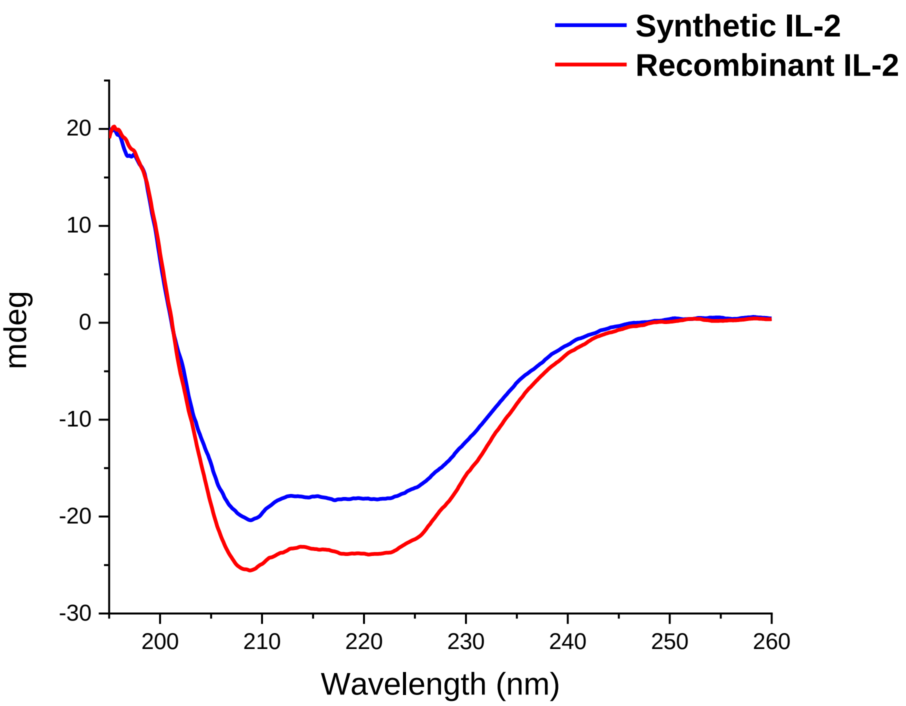

Figure 6.8-4 CD of Synthetic IL-2**.**

The recombinant IL-2 (HEK293) was purchased from MedChemExpress.

7. Synthesis of Salicylaldehyde Ester

7.1 Synthesis of Substituted Salicylaldehyde

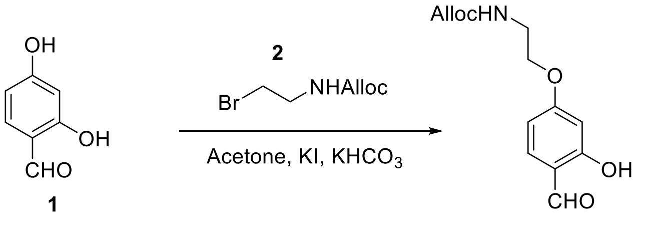

3

Compound **1** (1.38 g, 10.0 mmol), $KHCO_3$ (1.0 g, 10.0 mmol) and KI (0.16 g, 1.0 mmol) were charged into 30 mL acetone, then compound **2** (2.08 g, 10.0 mmol) was added. The mixture was refluxed for overnight. The resulting mixture was diluted with ethyl acetate (150 mL) and washed with H_2O (200 mL) and brine (200 mL). The organic phase was dried over anhydrous Na_2SO_4 and concentrated under vacuum. The residue was purified by silica gel chromatography (n-hexane/EtOAc = 5:1) to give 2-hydroxy-4-(prop-2-yn-1-yloxy)benzaldehyde **3** as a white solid (1.19 g, 45%)[1].

^{1}H NMR (400 MHz, $CDCl_3$) δ = 11.46 (s, 1H), 9.73 (s, 1H), 7.45 (d, *J* = 8.4 Hz, 1H), 6.54 (dd, *J* = 6.4, *J* = 2.4 Hz, 1H), 6.42 (d, *J* = 2.0 Hz, 1H), 5.98-5.88 (m, 1.0), 5.35 (d, *J* = 1.2 Hz, 1H), 5.23 (d, *J* = 10.1 Hz, 2H) 4.60 (d, *J* = 5.5 Hz, 2H), 4.10 (t, *J* = 5.1 Hz, 2H), 3.64 (q, *J* = 5.4 Hz, 2H).

^{13}C NMR (101 MHz, $CDCl_3$) δ = 194.5, 165.6, 164.4, 156.2, 135.4, 132.7, 118.0, 115.5, 108.3, 101.4, 67.4, 65.8, 40.2.

7.2 Synthesis of F-SAL

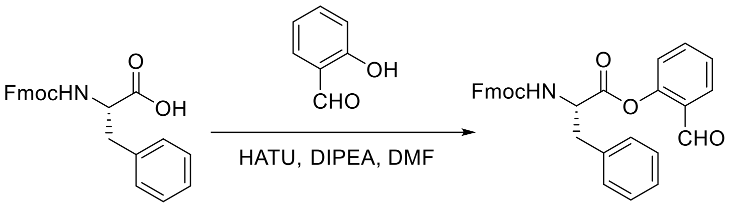

Fmoc-Phe-OH (1.91 g, 5 mmol) and HATU (1.90 g, 5 mmol) were dissolved in 25 mL DMF, then 1.5 mL DIPEA was added. The reaction mixture was stirred at r.t. for 5 min, and Salicylaldehyde (1.22 g, 10 mmol) was charged into the solution. This mixture was reacted for overnight. The resulting mixture was diluted with ethyl acetate (150 mL) and washed with H_2O (200 mL) and brine (200 mL). The organic phase was dried over anhydrous Na_2SO_4 and concentrated under vacuum. The residue was purified by silica gel chromatography (Hexane/EtOAc = 5:1) to give **F-SAL** as a white solid (1.23 g, 51%).

^{1}H NMR (400 MHz, $CDCl_3$) δ = 9.93 (s, 1H), 7.91 (d, *J* = 7.7 Hz, 2H), 7.79 (d, *J* = 7.6 Hz, 2H), 7.64 (t, *J* = 8.9 Hz, 1H), 7.59 (t, *J* = 7.3 Hz, 1H), 7.46-7.28 (m, 10H), 7.11 (d, *J* = 8.1 Hz, 1H), 5.42 (d, *J* = 8.0 Hz, 1H) 4.99 (q, *J* = 7.1 Hz, 1H) 4.50-4.40 (m, 2H), 4.25 (t, *J* = 6.9 Hz, 1H), 3.42-3.28 (m, 2H).

^{13}C NMR (101 MHz, $CDCl_3$) δ = 188.7, 170.2, 143.8, 143.75, 143.70, 141.3, 135.6, 135.3, 131.2, 129.4, 128.9, 127.8, 127.5, 127.1, 126.8, 125.1, 125.0, 123.2, 120.0, 67.2, 55.3, 47.1, 38.0.

HRMS (ESI+) for $C_{31}H_{25}NO_5$ (+) [M+H:]+ calcd 492.1733; found 492.1742.

7.3 Synthesis of F-SAL-Alloc

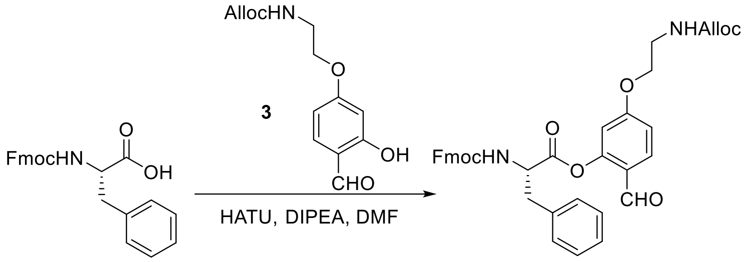

Fmoc-Phe-OH (3.83 g, 10 mmol) and HATU (3.80 g, 10 mmol) were dissolved in 50 mL DMF, then 3.5 mL DIPEA was added. The reaction mixture was stirred at r.t. for 5 min, and compound **3** (2.65 g, 10 mmol) was charged into the solution. This mixture was reacted for overnight. The resulting mixture was diluted with ethyl acetate (150 mL) and washed with H_2O (200 mL) and brine (200 mL). The organic phase was dried over anhydrous Na_2SO_4 and concentrated under vacuum. The residue was purified by silica gel chromatography (Hexane/EtOAc = 3:1) to give **F-SAL-Alloc** as a white solid (4.06g, 64%).

^{1}H NMR (400 MHz, $CDCl_3$) δ = 9.79 (s, 1H), 7.83-7.77 (m, 3H), 7.60-7.56 (m, 2H), 7.43-7.28 (m, 9H), 6.90 (dd, *J* = 8.8, *J* = 2.0 Hz, 1H), 6.55 (d, *J* = 1.2 Hz 1H), 5.95-5.94 (m, 1H), 5.55 (d, *J* = 8.0 Hz, 1H), 5.36 (d, *J* = 1.2 Hz, 1H), 5.25 (m, 2H), 4.97-4.96 (m, 1H), 4.61-4.60 (m, 2H), 4.48-4.47 (m, 2H), 4.40-4.4.30 (m, 1H), 4.25-4.21 (m, 2H), 3.63-3.62 (m, 2H), 3.39-3.32 (m, 2H).

^{13}C NMR (101 MHz, $CDCl_3$) δ = 187.5, 170.1, 163.9, 156.3, 155.8, 152.8, 143.7, 141.3, 135.7, 133.2, 132.7, 129.4, 128.8, 127.7, 127.4, 127.1, 125.1, 121.8, 120.0, 118.0, 112.9, 109.1, 67.7, 67.2, 65.8, 55.3, 47.1, 40.2, 37.9.

HRMS (ESI+) for $C_{37}H_{34}N_2O_8$ (+) [M+H:]+ calcd 635.2388; found 635.2378.

7.4 Synthesis of M-SAL-Alloc

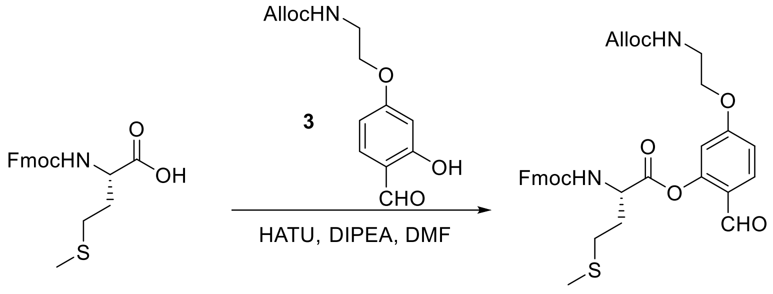

Fmoc-Met-OH (3.71 g, 10 mmol) and HATU (3.80 g, 10 mmol) were dissolved in 50 mL DMF, then 3.5 mL DIPEA was added. The reaction mixture was stirred at r.t. for 5 min, and compound **3** (2.65 g, 10 mmol) was charged into the solution. This mixture was reacted for overnight. The resulting mixture was diluted with ethyl acetate (150 mL) and washed with H_2O (200 mL) and brine (200 mL). The organic phase was dried over anhydrous Na_2SO_4 and concentrated under vacuum. The residue was purified by silica gel chromatography (Hexane/EtOAc = 3:1) to give **Fmoc-Met-Alloc** as a white solid (4.21g, 68%).

^{1}H NMR (400 MHz, $CDCl_3$) δ = 9.92 (s, 1H), 7.82-7.63 (m, 3H), 7.62-7.43 (m, 2H), 7.38-7.32 (m, 2H), 7.30-7.28 (m, 2H), 6.93 (dd, *J* = 8.8, *J* = 2.0 Hz, 1H), 6.71 (s, 1H), 5.94-5.89 (m, 1.0H), 5.72 (d, *J* = 8.4 Hz, 1H), 5.35 (d, *J* = 1.2 Hz, 1H) 5.31 (d, *J* = 1.2 Hz, 1H), 5.25-5.22 (m, 2H) 4.86-4.85 (m, 1H), 4.60-4.59 (m, 2H), 4.48-4.46 (m, 2H), 4.28-4.25 (m, 1H), 4.11-4.09 (m, 2H), 3.63-3.62 (m, 2H), 2.73-2.71 (m, 2H), 2.69-2.67 (m, 1H) 2.47-2.45 (m, 1H), 2.23 (s, 3H)

^{13}C NMR (101 MHz, $CDCl_3$) δ = 187.7, 170.3, 164.0, 156.3, 156.2, 152.4, 143.8, 143.7, 141.3, 134.4, 132.6, 127.8, 127.1, 121.8, 120.0, 118.0, 112.6, 109.6, 67.7, 67.2, 65.8, 53.3, 47.2, 40.2, 31.2, 30.2, 15.5.

HRMS (ESI+) for $C_{33}H_{34}N_2O_8S$ (+) [M+H:]+ calcd 619.2108; found 619.2090.

8. ^{1}H and ^{13}C NMR spectra

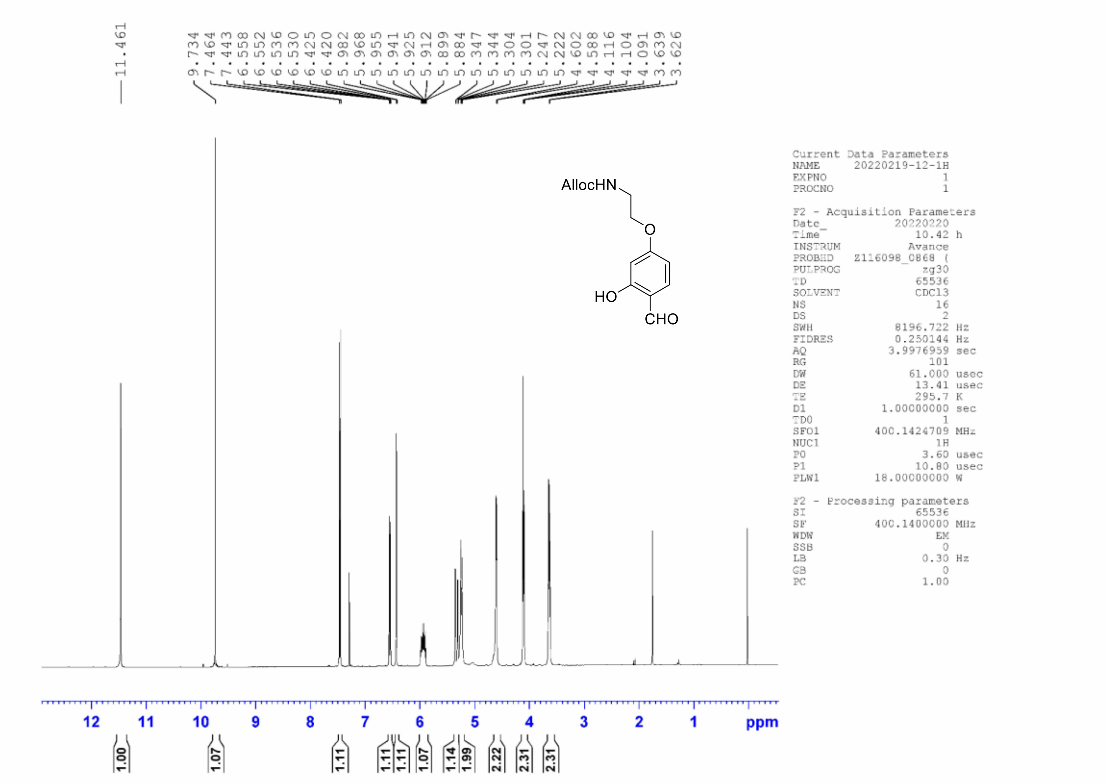

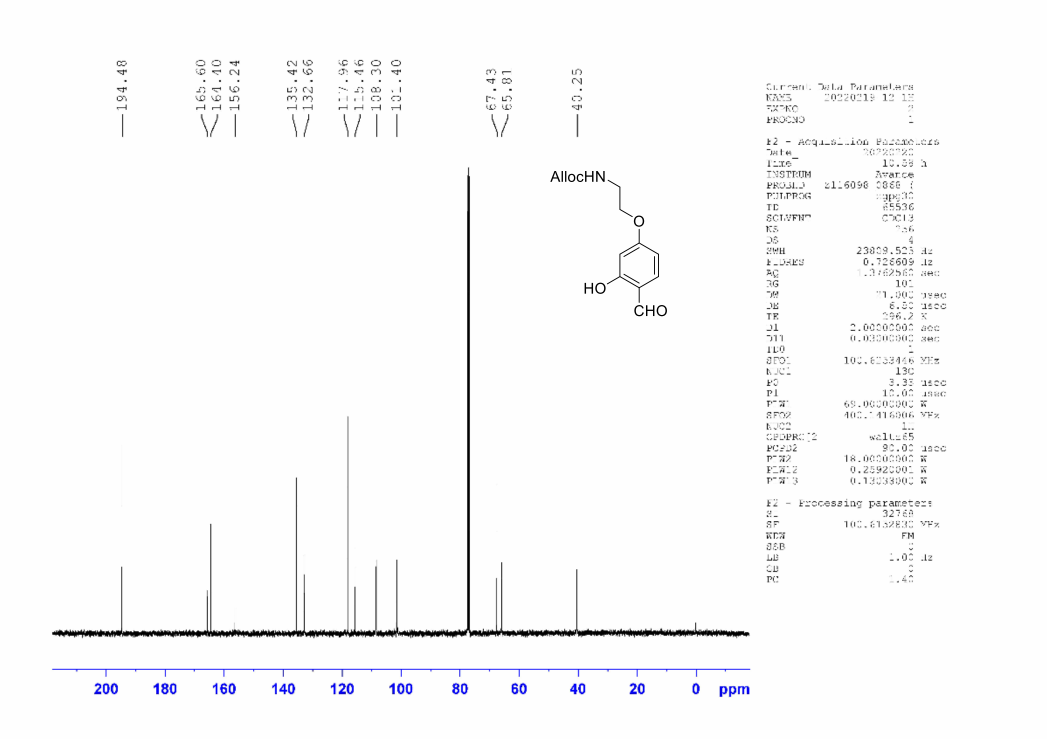

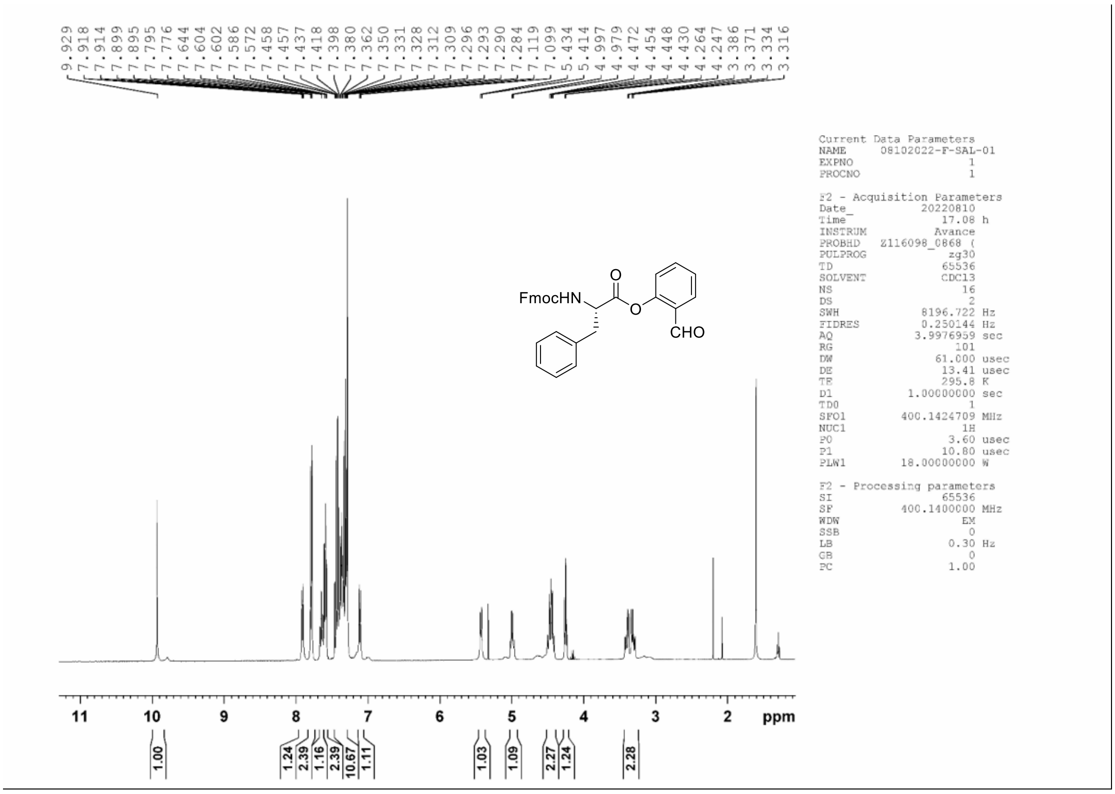
9.929 7.918 7.914 7.899 7.895 7.795 7.776 7.644 7.604 7.602 7.586 7.572 7.458 7.457 7.437 7.418 7.398 7.380 7.362 7.350 7.331 7.328 7.312 7.309 7.296 7.293 7.290 7.284 7.119 7.099 5.434 5.414 4.997 4.979 4.472 4.454 4.448 4.430 4.264 4.247 3.386 3.371 3.334 3.316
FmocHN
O
O
CHO
Current Data Parameters
NAME 08102022-F-SAL-01
EXPNO 1
PROCNO 1
F2 - Acquisition Parameters
Date_ 20220810
Time 17.08 h
INSTRUM Avance
PROBHD Z116098_0868 (
PULPROG zg30
TD 65536
SOLVENT CDCl3
NS 16
DS 2
SWH 8196.722 Hz
FIDRES 0.250144 Hz
AQ 3.9976959 sec
RG 101
DW 61.000 usec
DE 13.41 usec
TE 295.8 K
D1 1.00000000 sec
TD0 1
SFO1 400.1424709 MHz
NUC1 1H
P0 3.60 usec
P1 10.80 usec
PLW1 18.00000000 W
F2 - Processing parameters
SI 65536
SF 400.1400000 MHz
WDW EM
SSB 0
LB 0.30 Hz
GB 0
PC 1.00
11 10 9 8 7 6 5 4 3 2 ppm
1.00 1.24 2.39 1.16 2.39 10.67 1.11 1.03 1.09 2.27 1.24 2.28

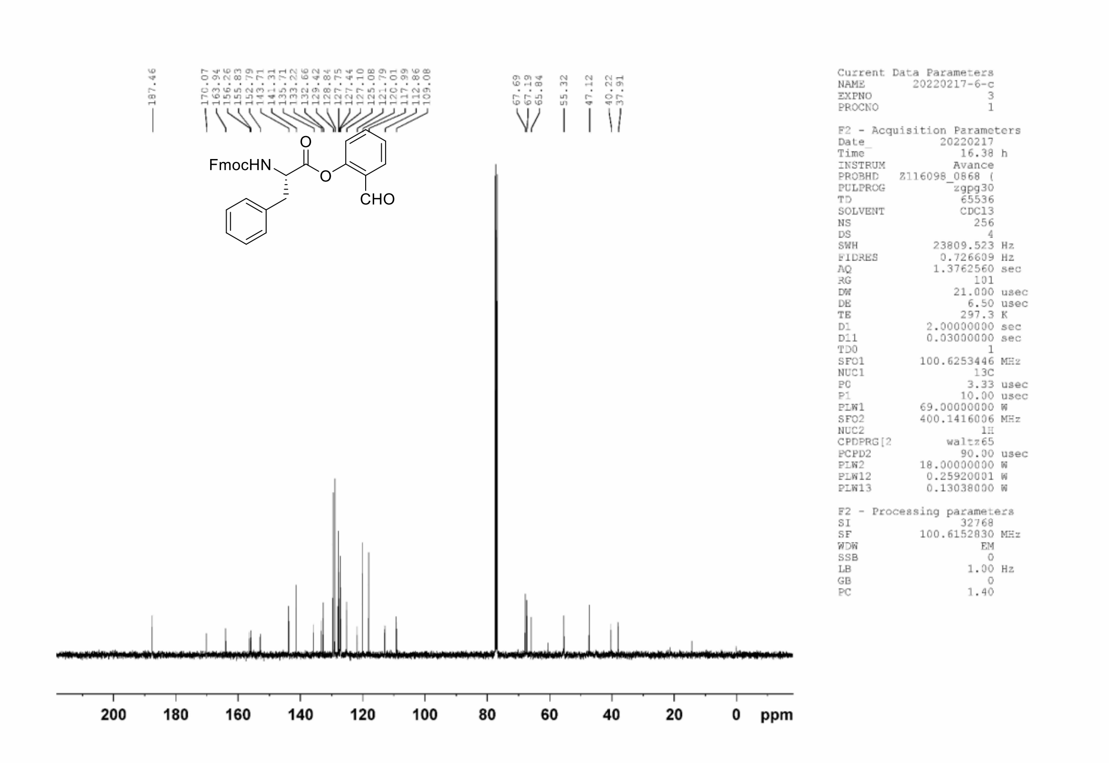
187.46 170.07 163.94 156.26 155.83 152.79 143.71 141.31 135.71 133.22 132.66 129.42 128.84 127.75 127.44 127.10 125.08 121.79 120.01 117.99 112.86 109.08 67.69 67.19 65.84 55.32 47.12 40.22 37.91
FmocHN
O
O
CHO
Current Data Parameters
NAME 20220217-6-c
EXPNO 3
PROCNO 1
F2 - Acquisition Parameters
Date_ 20220217
Time 16.38 h
INSTRUM Avance
PROBHD Z116098_0868 (
PULPROG zgpg30
TD 65536
SOLVENT CDCl3
NS 256
DS 4
SWH 23809.523 Hz
FIDRES 0.726609 Hz
AQ 1.3762560 sec
RG 101
DW 21.000 usec
DE 6.50 usec
TE 297.3 K
D1 2.00000000 sec
D11 0.03000000 sec
TD0 1
SFO1 100.6253446 MHz
NUC1 13C
P0 3.33 usec
P1 10.00 usec
PLW1 69.00000000 W
SFO2 400.1416006 MHz
NUC2 1H
CPDPRG[2 waltz65
PCPD2 90.00 usec
PLW2 18.00000000 W
PLW12 0.25920001 W
PLW13 0.13038000 W
F2 - Processing parameters
SI 32768
SF 100.6152830 MHz
WDW EM
SSB 0
LB 1.00 Hz
GB 0
PC 1.40
200 180 160 140 120 100 80 60 40 20 0 ppm

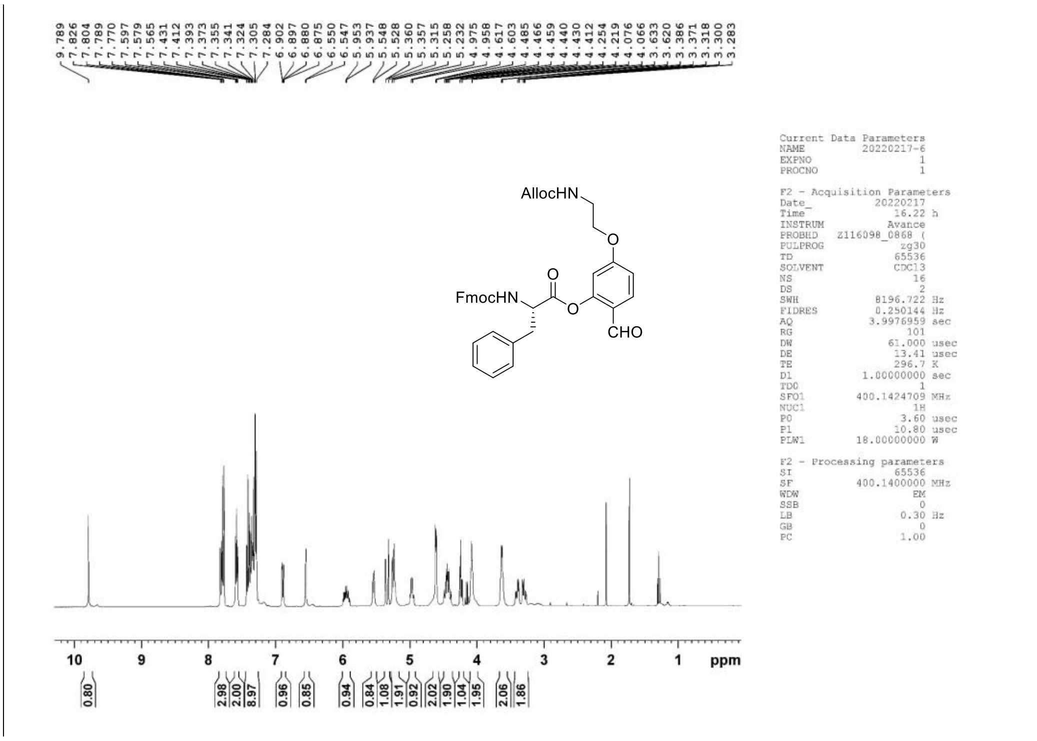
AllocHN
FmocHN
CHO
ppm

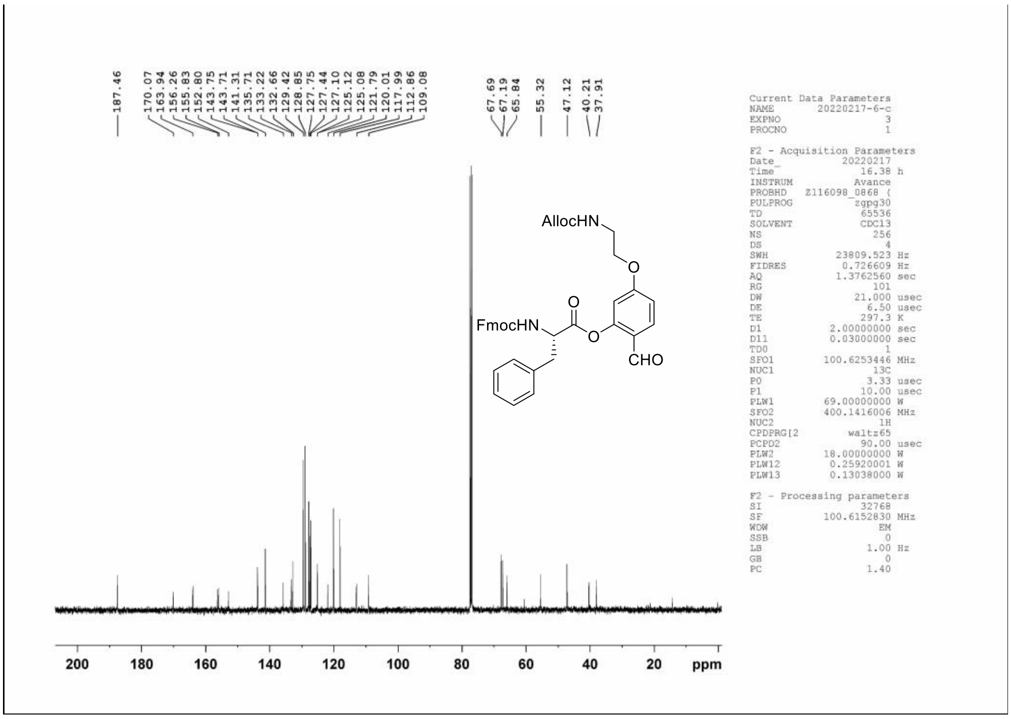
AllocHN
FmocHN
CHO
ppm

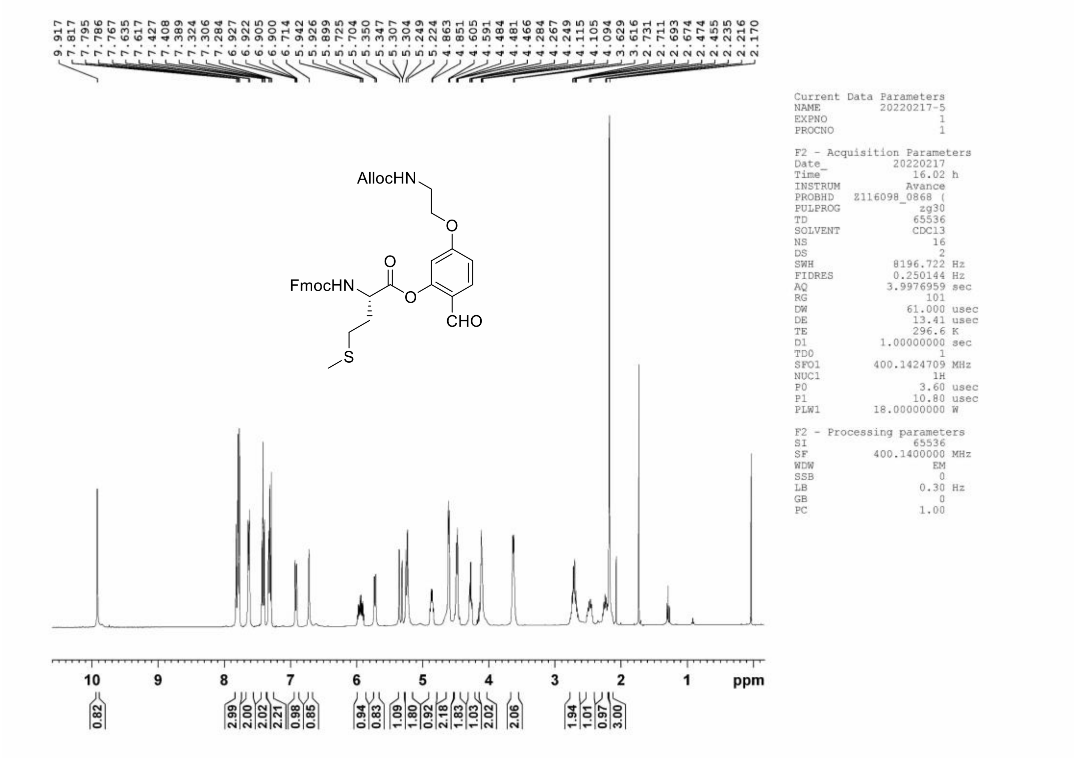
9.917
7.817
7.795
7.786
7.767
7.635
7.617
7.427
7.408
7.389
7.324
7.306
7.284
6.927
6.922
6.905
6.900
6.714
5.942
5.926
5.899
5.725
5.704
5.350
5.347
5.307
5.304
5.249
5.224
4.863
4.851
4.605
4.591
4.484
4.481
4.466
4.284
4.267
4.249
4.115
4.094
3.629
3.616
2.731
2.711
2.693
2.674
2.474
2.455
2.235
2.216
2.170
AllocHN
O
O
FmocHN
O
CHO
S
0.82
2.99
2.00
2.02
2.21
0.98
0.85
0.94
0.83
1.09
1.80
0.92
2.18
1.83
1.03
2.02
2.06
1.94
1.01
0.97
3.00
ppm
Current Data Parameters
NAME 20220217-5
EXPNO 1
PROCNO 1
F2 - Acquisition Parameters
Date_ 20220217
Time 16.02 h
INSTRUM Avance
PROBHD Z116098_0868 (
PULPROG zg30
TD 65536
SOLVENT CDCl3
NS 16
DS 2
SWH 8196.722 Hz
FIDRES 0.250144 Hz
AQ 3.9976959 sec
RG 101
DW 61.000 usec
DE 13.41 usec
TE 296.6 K
D1 1.00000000 sec
TD0 1
SFO1 400.1424709 MHz
NUC1 1H
P0 3.60 usec
P1 10.80 usec
PLW1 18.00000000 W
F2 - Processing parameters
SI 65536
SF 400.1400000 MHz
WDW EM
SSB 0
LB 0.30 Hz
GB 0
PC 1.00

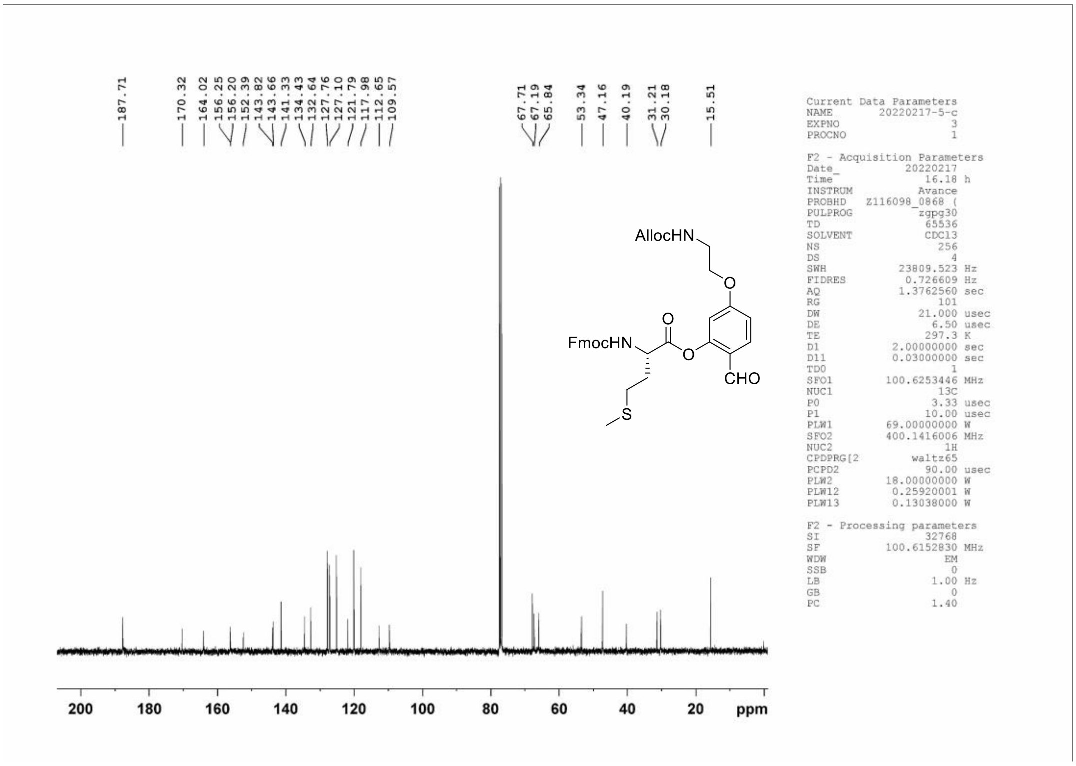
187.71
170.32
164.02
156.25
156.20
152.39
143.82
143.66
141.33
134.43
132.64
127.76
127.10
121.79
117.98
112.65
109.57
67.71
67.19
65.84
53.34
47.16
40.19
31.21
30.18
15.51
AllocHN
O
O
FmocHN
O
CHO
S
ppm
Current Data Parameters
NAME 20220217-5-c
EXPNO 3
PROCNO 1
F2 - Acquisition Parameters
Date_ 20220217
Time 16.18 h
INSTRUM Avance
PROBHD Z116098_0868 (
PULPROG zgpg30
TD 65536
SOLVENT CDCl3
NS 256
DS 4
SWH 23809.523 Hz
FIDRES 0.726609 Hz
AQ 1.3762560 sec
RG 101
DW 21.000 usec
DE 6.50 usec
TE 297.3 K
D1 2.00000000 sec
D11 0.03000000 sec
TD0 1
SFO1 100.6253446 MHz
NUC1 13C
P0 3.33 usec
P1 10.00 usec
PLW1 69.00000000 W
SFO2 400.1416006 MHz
NUC2 1H
CPDPRG[2 waltz65
PCPD2 90.00 usec
PLW2 18.00000000 W
PLW12 0.25920001 W
PLW13 0.13038000 W
F2 - Processing parameters
SI 32768
SF 100.6152830 MHz
WDW EM
SSB 0
LB 1.00 Hz
GB 0
PC 1.40

Reference

1. J. Zheng, M. Yu, Yun, Qi, S. Tang, F. Shen, Z. Wang, L. Xiao, L. Zhang, C. Tian, L. Lei, *J. Am. Chem. Soc.*, 2014, **136**, 3695–3704.
